# The developmental trajectory of fronto‐temporoparietal connectivity as a proxy of the default mode network: a longitudinal fNIRS investigation

**DOI:** 10.1002/hbm.24974

**Published:** 2020-03-04

**Authors:** Chiara Bulgarelli, Carina C. J. M. de Klerk, John E. Richards, Victoria Southgate, Antonia Hamilton, Anna Blasi

**Affiliations:** ^1^ Department of Medical Physics and Biomedical Engineering University College London London UK; ^2^ Centre for Brain and Cognitive Development, Birkbeck College University of London London UK; ^3^ Department of Psychology University of Essex Colchester UK; ^4^ Institute for Mind and Brain, Department of Psychology University of South Carolina Columbia South Carolina; ^5^ Department of Psychology University of Copenhagen Copenhagen Denmark; ^6^ Institute of Cognitive Neuroscience University College London London UK

**Keywords:** default mode network, developmental trajectory, fNIRS, fronto‐temporoparietal connectivity, functional connectivity, infants, resting‐state

## Abstract

The default mode network (DMN) is a network of brain regions that is activated while we are not engaged in any particular task. While there is a large volume of research documenting functional connectivity within the DMN in adults, knowledge of the development of this network is still limited. There is some evidence for a gradual increase in the functional connections within the DMN during the first 2 years of life, in contrast to other functional resting‐state networks that support primary sensorimotor functions, which are online from very early in life. Previous studies that investigated the development of the DMN acquired data from sleeping infants using fMRI. However, sleep stages are known to affect functional connectivity. In the current longitudinal study, fNIRS was used to measure spontaneous fluctuations in connectivity within fronto‐temporoparietal areas—as a proxy for the DMN—in awake participants every 6 months from 11 months till 36 months. This study validates a method for recording resting‐state data from awake infants, and presents a data analysis pipeline for the investigation of functional connections with infant fNIRS data, which will be beneficial for researchers in this field. A gradual development of fronto‐temporoparietal connectivity was found, supporting the idea that the DMN develops over the first years of life. Functional connectivity reached its maximum peak at about 24 months, which is consistent with previous findings showing that, by 2 years of age, DMN connectivity is similar to that observed in adults.

## 1 INTRODUCTION

Many studies have shown that our brain displays correlations between spontaneous fluctuations in activation in the low‐frequency range (<0.1 Hz) while we are not engaged in any specific task (for a recent review see Raichle, 2015). Resting‐state recording refers to the acquisition of this intrinsic brain activity during quiet wakefulness, in the absence of any cognitive, sensory or social stimulation (Biswal, Zerrin Yetkin, Haughton, & Hyde, 1995; Damoiseaux et al., 2006; van den Heuvel & Pol, 2010). The default mode network (DMN) is one of the most well‐known and most studied resting state networks (Raichle, 2015; Sporns, 2010). The DMN is composed of the medial prefrontal cortex (mPFC), the precuneus, the posterior and anterior cingulate cortex, the inferior parietal lobe (IPL), the medial temporal lobe and the temporoparietal junction (TPJ; Davey, Pujol, & Harrison, 2016; Greicius, Krasnow, Reiss, & Menon, 2003; Harrison et al., 2008; Mars et al., 2012; Molnar‐Szakacs & Uddin, 2013; Raichle, 2015; Schilbach, Eickhoff, Rotarska‐Jagiela, Fink, & Vogeley, 2008; Sporns, 2010). The importance of the DMN is underlined by several recent studies that have found that changes in the connectivity strength in this network are related to many psychopathologies (Broyd et al., 2009) and Alzheimer disease (Greicius, Srivastava, Reiss, & Menon, 2004). Furthermore, adult studies on the DMN suggest that this network is an “intrinsic system” that deals with self‐related signals and self‐processing (Golland, Golland, Bentin, & Malach, 2008). In fact, areas that are activated during self‐processing tasks show extensive overlap with the regions belonging to the DMN (Buckner & Carroll, 2007), and neuroimaging studies have shown that the DMN activity is positively correlated with participant reports of mind wandering and self‐related thoughts (Mason et al., 2007; McKiernan, D'Angelo, Kaufman, & Binder, 2006). Given the crucial role of the DMN is thought to play in self‐processing, it has been suggested that the gradual development of this functional network also supports the emergence of self‐awareness in the first years of life (Gao, Lin, Grewen, & Gilmore, 2016). Consistent with this view, it has been shown that the mPFC, a core region of the DMN, is more activated in response to self‐focused stimuli rather than externally‐focused stimuli (Xu et al., 2017) and to hearing one's own name rather than another's names (Imafuku, Hakuno, Uchida‐Ota, Yamamoto, & Minagawa, 2014) even before the first year of life.

The first study that explored resting‐state networks in the infant brain used fMRI with sleeping infants between 4 and 9 months (Fransson et al., 2007). This work showed evidence for the presence of visual and primary sensorimotor networks from birth, results that have since then been replicated several times (Gao, Alcauter, Smith, Gilmore, & Lin, 2015; Lin et al., 2008; Liu, Flax, Guise, Sukul, & Benasich, 2008). However, Fransson et al. did not find evidence for temporal synchronisation in core regions of the DMN before the first year of life (Fransson, Åden, Blennow, & Lagercrantz, 2011; Fransson et al., 2007, 2009). The early maturation of the primary sensory networks is thought to indicate that primary sensory functions, such as vision and touch, are in place from very early in life (even though functional networks also undergo significant change over the course of development, and adapt to the acquisition of new skills [for example see Marrus et al., 2018]). In comparison, the slow integration of regions belonging to the DMN in a unique network during the first years of life might be consistent with the gradual emergence of more advanced social cognitive abilities (Gao et al., 2016). More recent studies have discovered precursors of the DMN even before the first year of life. For example, functional correlations between core regions of the DMN were found in 4‐month‐old infants (for instance between the posterior cingulate cortex and the TPJ), but at this age, there was no significant correlation between the time series of the posterior and the anterior components (Damaraju, Caprihan, Lowe, et al., 2014). While short‐separation connectivity decreases with age, functional connectivity between more distant areas tends to increases with age, consistent with the idea of a gradual long‐range integration within the DMN (Damaraju, Caprihan, Lowe, et al., 2014). Gao et al. showed precursors of a primitive DMN even at 2 weeks of life, and they demonstrated that by 2 years of age the DMN is functionally similar to that observed in adults (Gao et al., 2009).

All the infant studies mentioned above acquired resting‐state data with fMRI in sleeping participants, while resting‐state studies on adults usually acquire data on awake participants, who are typically asked not to think about anything in particular. However, connectivity measured during sleep does not display the same patterns of connectivity measured during wakefulness (Horovitz et al., 2009). Additionally, sleep stages have an effect on estimates of functional connectivity (Mitra et al., 2017; Tagliazucchi & Laufs, 2014). Therefore, the occasional falling asleep of adult participants in the scanner has been a problem for resting‐state studies. To solve this issue, recent studies have shown that the use of non‐social movies or videos helps to keep participants awake, increases compliance, and helps prevent social or emotional thoughts during mind‐wandering (Anderson, Ferguson, Lopez‐Larson, & Yurgelun‐Todd, 2011; Cantlon & Li, 2013; Conroy, Singer, Guntupalli, Ramadge, & Haxby, 2013; Sabuncu et al., 2010). Likewise, previous studies have used non‐social videos to acquire resting‐state with fMRI in awake children (Müller, Kühn‐Popp, Meinhardt, Sodian, & Paulus, 2015; Vanderwal, Kelly, Eilbott, Mayes, & Castellanos, 2015; Xiao, Friederici, Margulies, & Brauer, 2016). Furthermore, in adults, consistency within participants has been found between resting‐state data acquired in a stimulus‐free context and data acquired during observation of non‐social videos, suggesting that observing such videos does not influence estimates of resting state connectivity significantly (Finn et al., 2017; Vanderwal et al., 2015).

As our knowledge of the development of the DMN thus far relies on data acquired from sleeping infants, it may possibly be incomplete. To compare infant and adult findings properly, resting‐state data needs to be collected in awake infants. The current study aimed to fill this gap by investigating the developmental trajectory of connectivity within the DMN in awake infants. For this purpose, functional near‐infrared spectroscopy (fNIRS) is a suitable neuroimaging method, as it is a non‐invasive technique that measures changes in concentration in oxy‐haemoglobin (HbO_2_) and deoxy‐haemoglobin (HHb) to index brain activation that can be used with awake infants (Elwell, 1995; Ferrari & Quaresima, 2012; Hoshi, 2016; Lloyd‐Fox, Blasi, & Elwell, 2010; Wilcox & Biondi, 2015). These characteristics, together with the fact that fNIRS is more robust to movement than other neuroimaging techniques, make this method highly suitable for acquiring resting‐state recordings in infants under conditions similar to those typically used in studies with adults.

To our knowledge, only a few infant studies have measured spontaneous fluctuations in blood oxygenation during resting‐state using fNIRS, but on sleeping participants (Homae et al., 2010; Konishi, Taga, Yamada, & Hirasawa, 2002; Taga et al., 2000). In particular, Homae et al. (2010) recorded resting‐state in a longitudinal sample of sleeping infants at 4 days, at 3 and 6 months. An increase in functional connectivity was shown between the frontal, temporal, parietal and occipital regions. Additionally, while in the neonates connections were detected mainly within the same hemisphere, a more bilateral organisation of spontaneous networks emerged around the third month of life, when clusters of connections started to form across the midline (Homae et al., 2010). Recent adult studies have also used fNIRS to assess resting‐state functional connectivity, suggesting it is a promising tool for this purpose (Lu et al., 2010; Mesquita, Franceschini, & Boas, 2010; Sasai et al., 2012). However, due to the fact that the near‐infrared light can only penetrate a couple of centimetres into the scalp, its use is limited to the outer layers of the cortex. Therefore, in this study we measured connectivity between frontal, temporal, and parietal brain areas, which we will refer to as fronto‐temporoparietal connectivity, as a proxy for the DMN. The approach of studying portions of the DMN as a proxy for this network has been recently adopted by adult studies, focusing in particular on the mPFC (Durantin, Dehais, & Delorme, 2015; Liang, Chen, Shewokis, & Getchell, 2016; Sasai et al., 2012) and the parietal lobes (Rosenbaum et al., 2017; Sasai et al., 2012).

To assess the developmental trajectory of fronto‐temporoparietal connectivity, resting‐state data were acquired with fNIRS in a longitudinal study at five time points. Participants were tested with the same resting‐state procedure every 6 months, from 11 to 36 months. Regular intervals of data acquisition throughout the first 3 years of life allowed to capture the rapid neural development that takes place during this time (Johnson, 2001; Yamada et al., 1997). We hypothesised that we could find a gradual increase of fronto‐temporoparietal connectivity over the first 3 years of life.

## 2 METHODS

### 2.1 Participants

fNIRS resting‐state data were acquired longitudinally when infants were 11, 18, 24, 30 and 36 months old.^1^ Refer Table 1 for demographic information of the participants at each visit. All included infants were born full‐ term, healthy and with normal birth weight. Written informed consent was obtained from the infant's caregiver prior to the start of the experiment.

**Table 1:** Characteristics of included and excluded participants at each time point

		11 months	18 months	24 months	30 months	36 months
Included participants	*N*	11	21	25	28	32
	Age in days (mean ± *SD*)	342.72 ± 8.10	554.73 ± 9.19	737.61 ± 14.10	918.75 ± 8.68	1,101.13 ± 16.03
	Sex (M, F)	6, 5	10, 11	11, 14	18, 10	23,9
	RS in seconds considered for the analysis (mean ± *SD*, range)	134.25 ± 57.15, 100.28–292.96	196.64 ± 60.69, 112.92–323.08	183.75 ± 58.60, 100.76–304.96	200.44 ± 47.95, 115–277.72	177.02 ± 45.49, 100.08–268.36
Excluded participants	The dataset did not reach the minimum length of 100 s of recording after behavioural coding	21	7	6	3	8
	Refused to wear the fNIRS hat or poor positioning of the hat	9	15	6	6	/
	More than 30% of the channels had to be excluded (poor light intensity readings)	5	5	6	9	8

Infants were excluded from the analysis if (a) their dataset did not reach the minimum length of 100 s of recording after behavioural coding (see section 2.5 for more details); (b) they refused to wear the NIRS hat or poor positioning of the NIRS hat; (c) more than 30% of the channels had to be excluded due to poor light intensity readings. Refer Table 1 for details on the included and excluded participants at each visit. Supporting Information reports the distribution of the bins lengths considered for the analysis at each age.

### 2.2 fNIRS recording and arrays configurations

fNIRS data were recorded using the UCL‐NIRS topography system, which uses two continuous wavelengths of near‐infrared light (770 and 850 nm) to detect changes in HbO_2_ and HHb concentrations (Everdell et al., 2005). Sampling rate of data acquisition was 10 Hz, and the mean power emitted by each laser diode was approximately 2 mW (Everdell et al., 2005).

**Figure 1:**
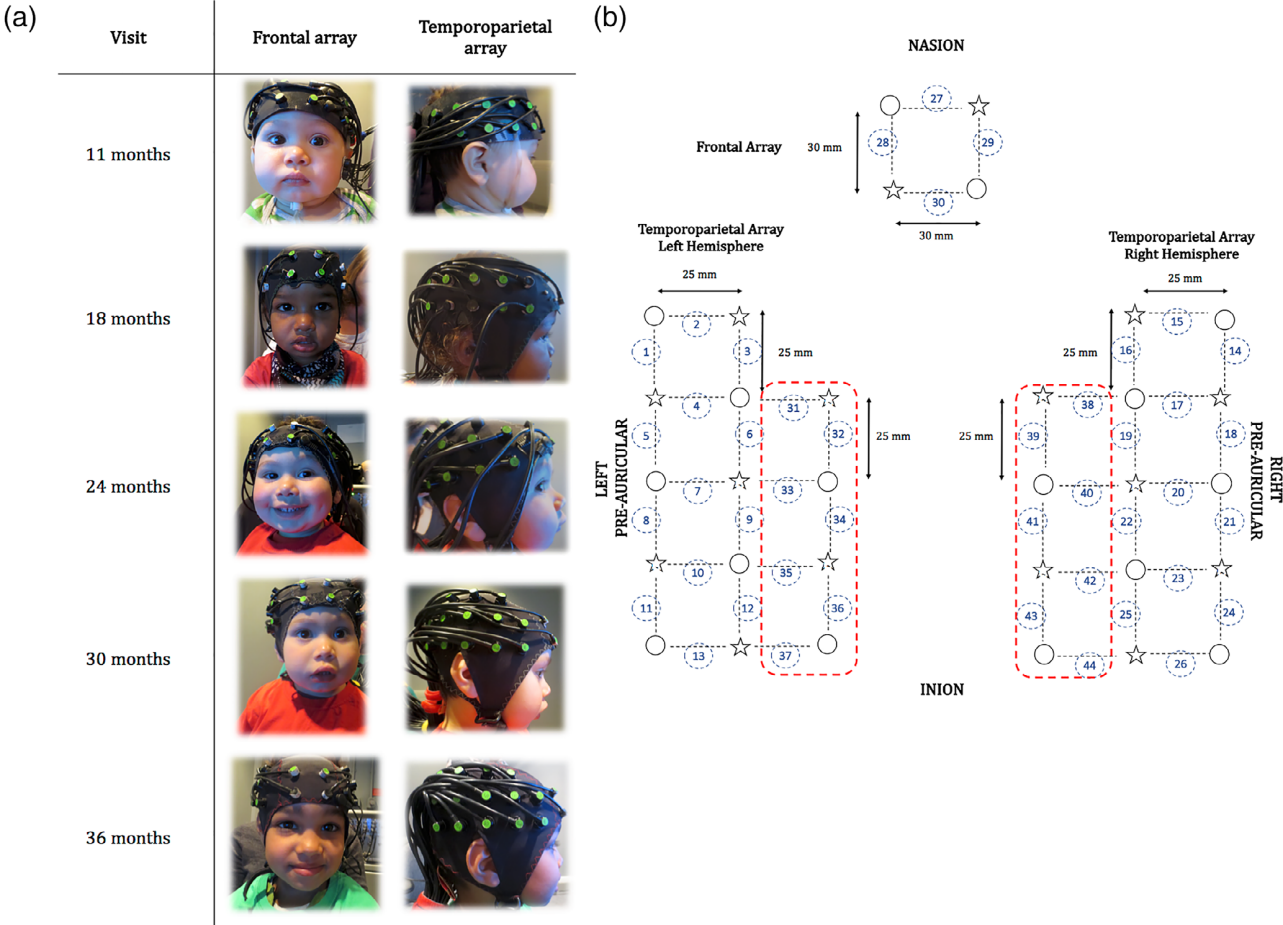
(a) Pictures of the participants wearing the fNIRS silicon band/Easycap at every visit. (b) Representation of the fNIRS arrays. Sources are marked with stars, detectors are marked with circles, channels are marked with black dotted lines and numbered with circles. The red dotted lines highlight the additional rows of optodes that added 14 channels. Figure 1b has been reproduced from Bulgarelli, et al. 2019

At the 11th month visit, infants wore a custom‐built headgear with a total of 30 channels. Data acquired at the other visits were collected using Easy Cap, caps made of soft black fabric, which provided a better fit on the participant's head, considering the increasing amount of hair (Figure 1a). At every visit, the custom‐made NIRS array covered the temporal, parietal, and frontal areas bilaterally and two very similar designs were used to acquire data. The first array design included 12 sources and 12 detectors to create a total of 30 channels and it was used at the 11th and the 18th month visit; the second array design included 16 sources and 16 detectors that made up a total of 44 channels. The 44‐channel configuration was an extension of the 30‐channel configuration and included two additional rows of optodes that added seven channels per hemisphere, in a superior location to the two existing lateral arrays. This allowed us to improve detection of the spontaneous fluctuation over the temporoparietal region, a core area of interest for this study. The 44‐channel configuration was used at the 24th, 30th and 36th month visit. Both configurations shared the design and the location of the channels covering frontal, inferior frontal and temporal regions (30 channels out of 44; Figure 1b).

The silicon band and the Easy Cap were placed so that the third lower optode of the temporal array was centred above the pre‐auricular point and that the two lower optodes of the frontal array centred over the nasion. Three differently sized EasyCap (48, 50 and 52 cm of circumference) were used to take into account variations in the infants’ head circumferences.^2^ Source‐detector (S‐D) separation was approximately 30 mm over the frontal lobe and 25 mm over the temporoparietal lobe. Given that the cortex is approximately 0.75 cm from the skin surface (Glenn, 2010) and based on studies on the transportation of near‐infrared light through brain tissue, these S‐D separations were predicted to penetrate up to a depth of approximately 12.5–15 mm from the skin surface, allowing measurement of both the gyri and parts of the sulci near the surface of the cortex (Lloyd‐Fox et al., 2010). S‐D separation increased slightly due to the stretch of the cap on the head and also due to re‐scaling based on the cap size.

Table 2 summarises information about the array design used at each visit, the number of participants tested with each cap size, and the S‐D separation for each cap size.

**Table 2:** Summary of the array design used at each visit, number of participants tested with each cap size, and S‐D separation

	11 months	18 months	24 months	30 months	36 months
	Silicone band	Easy cap	Easy cap	Easy cap	Easy cap
	30‐channel	30‐channel	44‐channel	44‐channel	44‐channel
Silicone band 25 mm S‐D temporal lobe 30 mm S‐D frontal lobe	11	/	/	/	/
EasyCap 48 cm 25 mm S‐D temporal lobe 30 mm S‐D frontal lobe	/	17	5	5	3
EasyCap 50 cm 26 mm S‐D temporal lobe 31 mm S‐D frontal lobe	/	4	18	16	18
EasyCap 52 cm 27 mm S‐D temporal lobe 32 mm S‐D frontal lobe	/	/	2	7	12
Total participants tested	11	21	25	28	32

### 2.3 Resting‐state data acquisition

**Figure 2:**
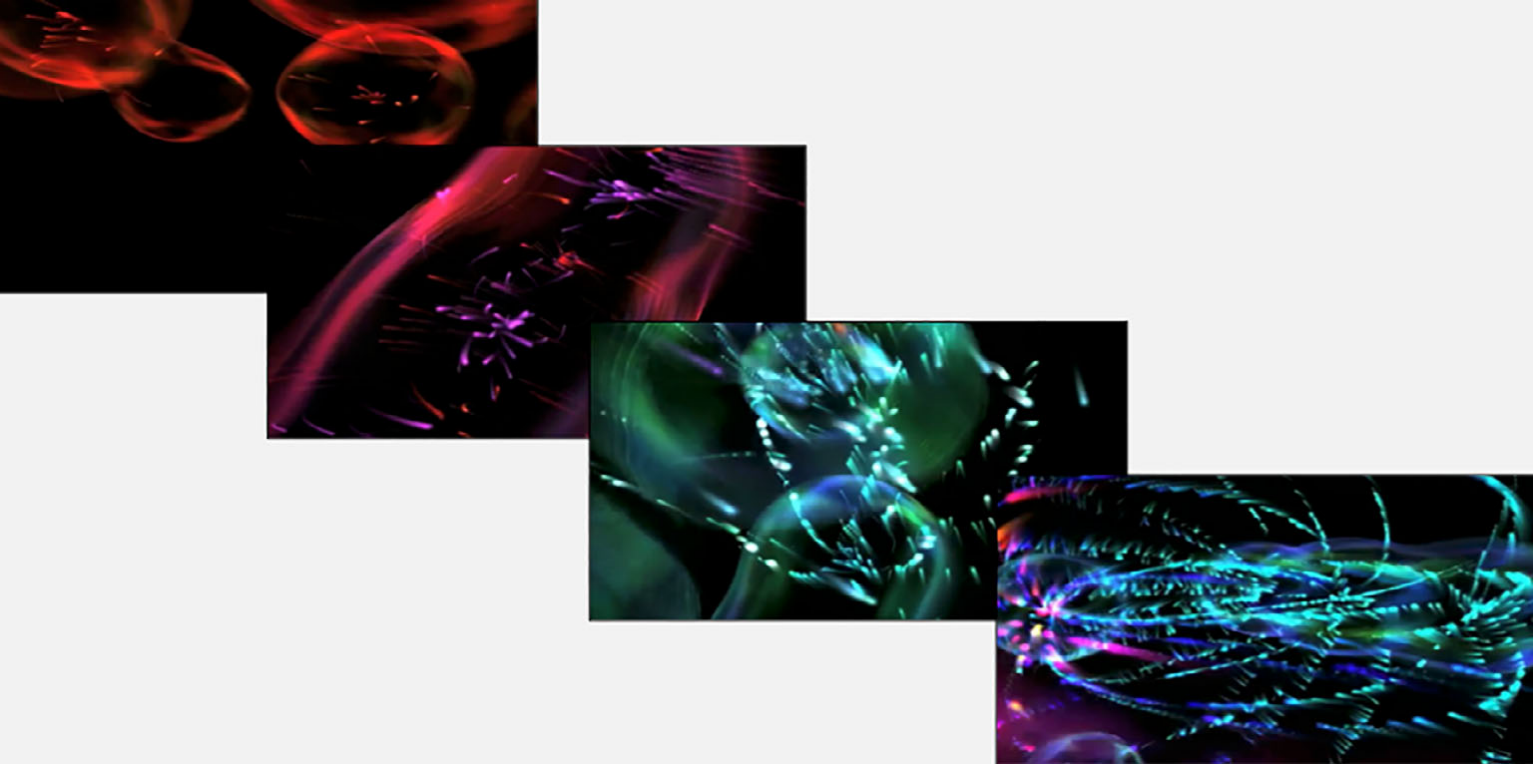
Still frames of the screensaver‐like video shown during the resting‐state acquisition. Figure 2 has been reproduced from Bulgarelli, et al. 2019

The resting‐state acquisition took place in a dimly lit and sound attenuated room, with the infant sitting on their parent's lap at approximately 90 cm from a 117 cm plasma screen. The resting‐state acquisition lasted until the participant became fussy, or until 6 min of data was recorded. To keep the infants awake and as still as possible, we showed them a screensaver‐like video with colourful bubbles accompanied by relaxing music (Figure 2). The parent was asked not to talk during the experiment to avoid brain activation in areas of interest. If the parent talked to redirect the infant's attention to the screen or in case of fussiness or distraction, we excluded this chunk of data from the recording (see section 2.5 for more details).

### 2.4 Co‐registration of the fNIRS array

After the acquisition of the resting‐state data, we logged the location of fNIRS array using the Polhemus Digitising System (http://polhemus.com/scanning-digitizing/digitizing-products/) if the participant was still compliant, to allow us to co‐register the fNIRS array on MRI structural scans. We registered five reference points (nasion, inion, right ear, left ear, Cz^3^) and the location of the fNIRS optodes. In order to log the reference points and the optodes location as precise as possible, it was fundamental to keep the infants quiet and to limit their movements during the recording. Therefore, during the Polhemus recording, we showed them infant‐friendly videos (e.g. clips from “In the Night Garden”). A marker placed on the back of the participant's cap allowed us to correct for head motion during the recording.

**Figure 3:**
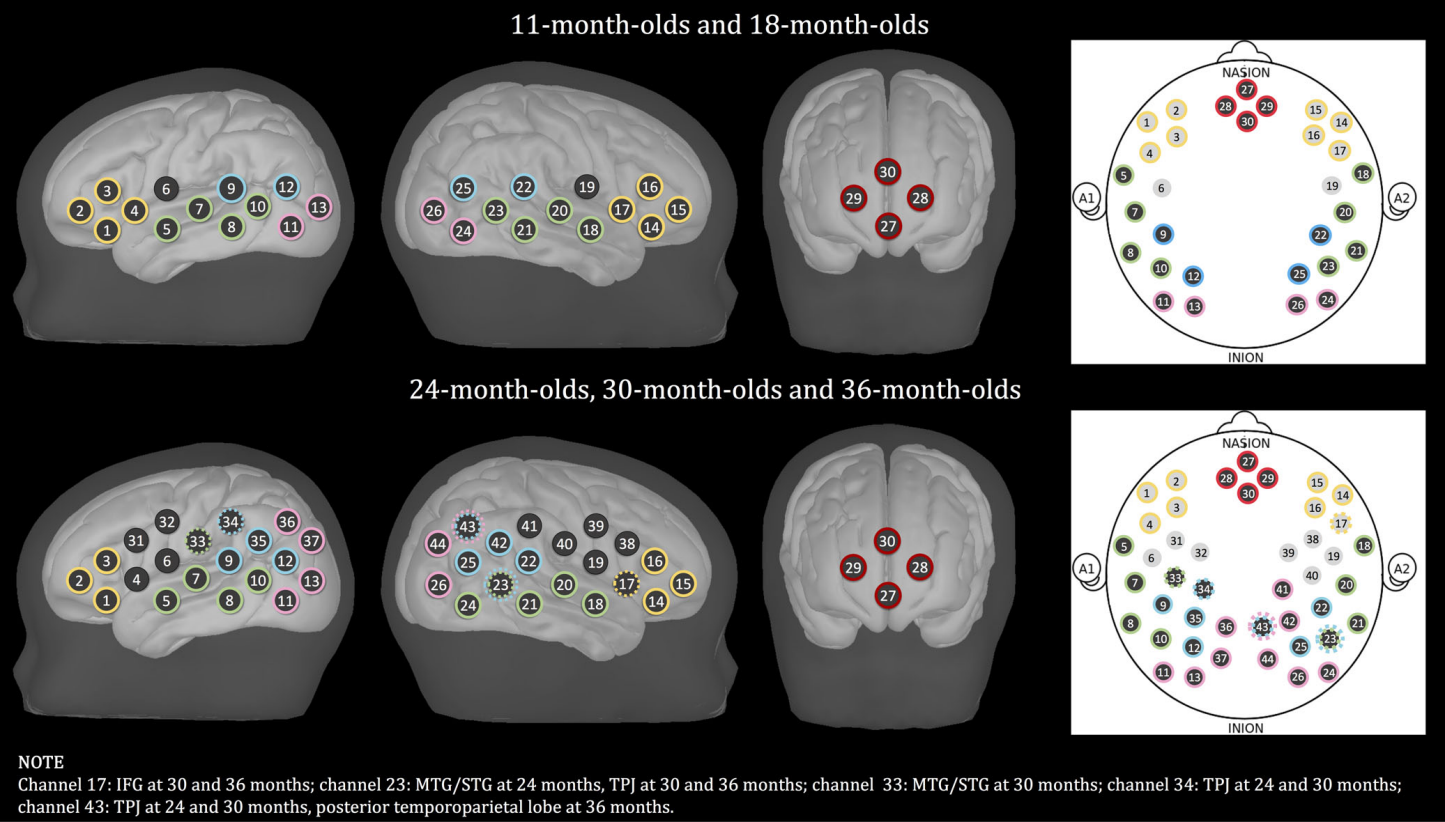
Representation of the channels on a MRI template both for the 30‐channel and the 44‐channel configuration. ROIs are colour‐coded: Red represents mPFC; yellow represents IFG; green represents MTG/STG; blue represents TPJ; pink represents posterior temporoparietal lobe. Dotted circles mark channels belong to different ROIs at different ages

**Figure 4:**
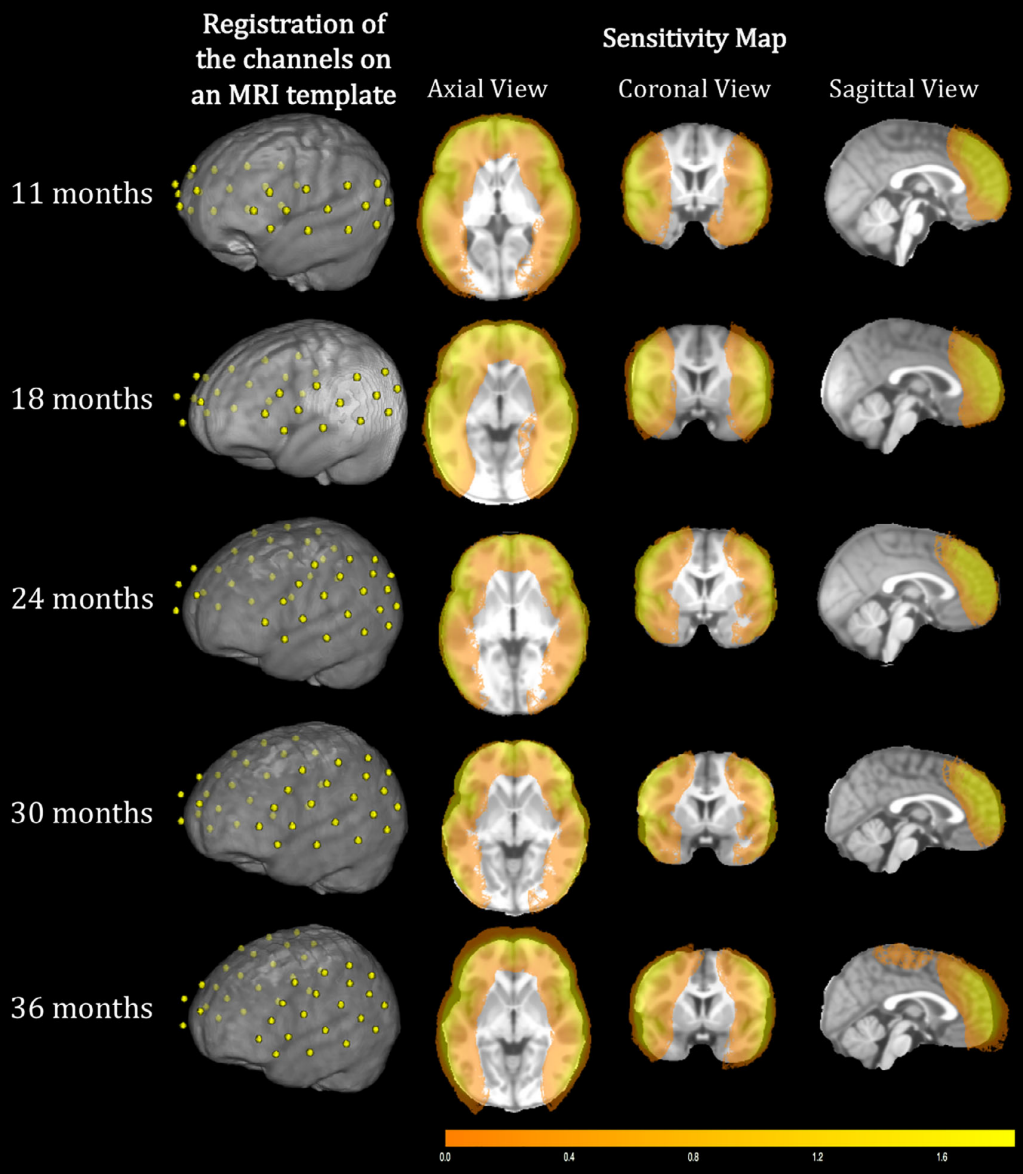
Registration of the channels on MRI templates and light attenuation changes in brain regions covered by the fNIRS array at every age

At every visit, we selected the best digitised recordings, based on the accuracy of the points marked in space compared to the optode locations in the pictures of the participant wearing the fNIRS cap that were taken after the recording (one from the front and two from the sides). Only participants who had a maximum of two mismatched points between the pictures and the digitised optode locations were included in the coregistration (11‐month‐olds [23], 18‐month‐olds [24], 24‐month‐olds [13], 30‐month‐olds [13], 36 month‐olds [26]). For each of these recordings, a structural MRI of an infant close in age with a similar head shape and size—based on head measurements collected before the testing session—was selected from the Neurodevelopmental MRI Database of the University of South Carolina (http://jerlab.psych.sc.edu/NeurodevelopmentalMRIDatabase/). For each channel, a spatial projection from the scalp to the cortex was estimated, and a 1.5‐cm radius sphere was calculated around the projected channel. The photon migration simulation was calculated for each channel using MCX (Fang & Boas, 2009), which estimates the paths of the photons from the source to the detector through the cortex. A cut‐off of 25% of the voxels surrounding the spatial projection point was used to determine the anatomical label for each channel. Table 3 lists the anatomical labels (LPBA40 atlas) associated with each channel at each age belonging to the array design described in section 2.2. Table 4 describes channels belonging to each ROI at each age. Figure 3 provides a graphical representation of the brain areas covered by the fNIRS array used at every age, where the ROIs are colours coded. Figure 4 is a sensitivity map, showing brain regions that are sensitive to light attenuation changes given the fNIRS array we used in this work.

**Table 3:** Co‐registration of each channel of the fNIRS array per each age, based on LPBA40 atlas

Channel	11 months	18 months	24 months	30 months	36 months
1	Inferior frontal gyrus	Inferior frontal gyrus	Inferior frontal gyrus	Inferior frontal gyrus	Inferior frontal gyrus, superior temporal gyrus
2	Inferior frontal gyrus	Inferior frontal gyrus	Inferior frontal gyrus	Inferior frontal gyrus	Inferior frontal gyrus
3	Inferior frontal gyrus	Inferior frontal gyrus, middle frontal gyrus	Inferior frontal gyrus	Inferior frontal gyrus	Inferior frontal gyrus
4	Inferior frontal gyrus	Inferior frontal gyrus	Precentral gyrus, superior temporal gyrus	Inferior frontal gyrus, precentral gyrus	Inferior frontal gyrus, precentral gyrus
5	Middle temporal gyrus, Superior temporal gyrus	Middle temporal gyrus, superior temporal gyrus	Middle temporal gyrus, superior temporal gyrus	Middle temporal gyrus	Middle temporal gyrus, superior temporal gyrus
6	Precentral gyrus	Postcentral gyrus, precentral gyrus	Postcentral gyrus	Inferior temporal gyrus, precentral gyrus	Postcentral gyrus, Precentral gyrus
7	Middle temporal gyrus, superior temporal gyrus	Postcentral gyrus, superior temporal gyrus	Middle temporal gyrus, superior temporal gyrus	Middle temporal gyrus, superior temporal gyrus	Superior temporal gyrus
8	Middle temporal gyrus	Middle temporal gyrus, superior temporal gyrus	Inferior temporal gyrus, middle temporal gyrus	Middle temporal gyrus	Middle temporal gyrus, superior temporal gyrus
9	Superior temporal gyrus, angular gyrus	Supramarginal gyrus	Supramarginal gyrus	Supramarginal gyrus	Superior temporal gyrus, supramarginal gyrus
10	Middle temporal gyrus, superior temporal gyrus	Middle temporal gyrus, superior temporal gyrus	Middle temporal gyrus	Middle temporal gyrus	Middle temporal gyrus
11	Inferior temporal gyrus, middle temporal gyrus	Middle temporal gyrus	Inferior temporal gyrus	Inferior temporal gyrus, middle temporal gyrus	Middle temporal gyrus
12	Angular gyrus, middle temporal gyrus	Angular gyrus	Angular gyrus, middle occipital gyrus	Angular gyrus	Angular gyrus
13	Inferior temporal gyrus, middle temporal gyrus	Angular gyrus	Middle occipital gyrus	Angular gyrus, middle occipital gyrus	Angular gyrus
14	Inferior frontal gyrus	Inferior frontal gyrus	Inferior frontal gyrus	Inferior frontal gyrus, middle frontal gyrus	Inferior frontal gyrus, middle frontal gyrus
15	Inferior frontal gyrus, middle frontal gyrus	Inferior frontal gyrus	Inferior frontal gyrus	Inferior frontal gyrus	Inferior frontal gyrus
16	Inferior frontal gyrus, middle frontal gyrus	Inferior frontal gyrus, middle frontal gyrus	Inferior frontal gyrus	Inferior frontal gyrus	Inferior frontal gyrus
17	Inferior frontal gyrus	Inferior frontal gyrus	Precentral gyrus	Inferior frontal gyrus	Inferior frontal gyrus
18	Medial temporal gyrus, superior temporal gyrus	Medial temporal gyrus, superior temporal gyrus	Middle temporal gyrus, superior temporal gyrus	Middle temporal gyrus	Middle temporal gyrus, superior temporal gyrus
19	Inferior frontal gyrus, precentral gyrus	Inferior frontal gyrus, precentral gyrus	Postcentral gyrus, supramarginal gyrus	Postcentral gyrus, precentral gyrus	Precentral gyrus
20	Postcentral gyrus, superior temporal gyrus	Postcentral gyrus, superior temporal gyrus	Middle temporal gyrus, superior temporal gyrus	Postcentral gyrus, superior temporal gyrus	Postcentral gyrus, superior temporal gyrus
21	Middle temporal gyrus	Middle temporal gyrus, superior temporal gyrus	Middle temporal gyrus	Middle temporal gyrus	Middle temporal gyrus, superior temporal gyrus
22	Postcentral gyrus, angular gyrus	Postcentral gyrus, angular gyrus	Supramarginal gyrus	Supramarginal gyrus	Postcentral gyrus, supramarginal gyrus
23	Middle temporal gyrus, superior temporal gyrus	Middle temporal gyrus, superior temporal gyrus	Middle temporal gyrus	Superior temporal gyrus, supramarginal gyrus	Superior temporal gyrus, supramarginal gyrus
24	Inferior temporal gyrus, middle temporal gyrus	Middle temporal gyrus	Middle temporal gyrus	Middle temporal gyrus	Middle temporal gyrus
25	Superior temporal gyrus, angular gyrus	Superior temporal gyrus, angular gyrus	Angular gyrus	Angular gyrus	Angular gyrus, Supramarginal gyrus
26	Middle temporal gyrus	Middle temporal gyrus, angular gyrus	Angular gyrus, middle occipital gyrus	Angular gyrus	Angular gyrus
27	Superior frontal gyrus	Superior frontal gyrus	Middle frontal gyrus, superior frontal gyrus	Middle frontal gyrus, superior frontal gyrus	Middle frontal gyrus, superior frontal gyrus
28	Middle frontal gyrus, superior frontal gyrus	Middle frontal gyrus, superior frontal gyrus	Middle frontal gyrus	Middle frontal gyrus, superior frontal gyrus	Superior frontal gyrus
29	Middle frontal gyrus, superior frontal gyrus	Middle frontal gyrus, superior frontal gyrus	Middle frontal gyrus, superior frontal gyrus	Superior frontal gyrus	Superior frontal gyrus
30	Superior frontal gyrus	Superior frontal gyrus	Superior frontal gyrus	Middle frontal gyrus	Middle frontal gyrus
31	/	/	Precentral gyrus	Inferior frontal gyrus, middle frontal gyrus	Inferior frontal gyrus, precentral gyrus
32	/	/	Postcentral gyrus, supramarginal gyrus	Middle frontal gyrus, middle temporal gyrus	Middle frontal gyrus, precentral gyrus
33	/	/	Supramarginal gyrus	Middle temporal gyrus	Postcentral gyrus
34	/	/	Angular gyrus, supramarginal gyrus	Angular gyrus, postcentral gyrus	Postcentral gyrus, supramarginal gyrus
35	/	/	Angular gyrus	Supramarginal gyrus	Supramarginal gyrus
36	/	/	Angular gyrus	Angular gyrus, supramarginal gyrus	Angular gyrus supramarginal gyrus
37	/	/	Angular gyrus, middle occipital gyrus	Angular gyrus	Angular gyrus
38	/	/	Precentral gyrus	Middle frontal gyrus	Middle frontal gyrus
39	/	/	Postcentral gyrus, precentral gyrus	Middle frontal gyrus, precentral gyrus	Middle frontal gyrus
40	/	/	Postcentral gyrus, supramarginal gyrus	Postcentral gyrus	Postcentral gyrus, precentral gyrus
41	/	/	supramarginal gyrus	Postcentral gyrus	Postcentral gyrus, precentral gyrus
42	/	/	Angular gyrus, supramarginal gyrus	Supramarginal gyrus	Supramarginal gyrus
43	/	/	Angular gyrus	Angular gyrus, supramarginal gyrus	Postcentral gyrus, supramarginal gyrus
44	/	/	Angular gyrus	Angular gyrus	Angular gyrus

**Table 4:** Channels belonging to the ROIs at every age

	Channels				
ROI	11 months	18 months	24 months	30 months	36 months
Left IFG	1, 2, 3, 4	1, 2, 3, 4	1, 2, 3	1, 2, 3	1, 2, 3
Right IFG	14, 15, 16, 17	14, 15, 16, 17	14, 15, 16, 17	14, 15, 16, 17	14, 15, 16, 17
Left MTG/STG	5, 7, 8, 10	5, 7, 8, 10	5, 7, 8, 10	5, 7, 8, 10	5, 7, 8, 10
Right MTG/STG	18, 20, 21, 23	18, 20, 21, 23	18, 20, 21, 23, 24	18, 20, 21, 24, 33	18, 20, 21, 24
Left posterior temporoparietal lobe	11, 13	11,13	11,13, 36, 37	11, 13, 36, 37	11, 13, 36, 37
Right posterior temporoparietal lobe	24, 26	24, 26	26, 44	26, 44	26, 43, 44
Left TPJ	9, 12	9, 12	9, 12, 34, 35	9, 12, 34, 35	9, 12, 35
Right TPJ	22,25	22, 25	22, 25, 42, 43	22, 23, 25, 42, 43	22, 23, 25, 42
mPFC	27, 28, 29, 30	27, 28, 29, 30	27, 28, 29, 30	27, 28, 29, 30	27, 28, 29, 30

In this study, the connections between the frontal region and the temporoparietal region were defined as the connections between channels belonging to the mPFC and the left and right MTG/STG, left and right posterior temporoparietal lobe, left and right TPJ.^4^ Our array covers also the IFG, but we have decided not to include this region in the DMN analyses of this study. In fact, most of the foundational papers on the DMN did not include the IFG as part of this network (Davey et al., 2016; Fox & Raichle, 2007; Horn, Ostwald, Reisert, & Blankenburg, 2014; Molnar‐Szakacs & Uddin, 2013; Raichle, 2015; Raichle et al., 2001; Sharaev, Zavyalova, Ushakov, Kartashov, & Velichkovsky, 2016). There is only one study that acknowledged the IFG as part of the DMN (Yeo et al., 2011), but in relation to a more rostral portion of this region, which we did not cover with the array used in this study.

### 2.5 Resting‐state data pre‐processing and analysis

**Figure 5:**
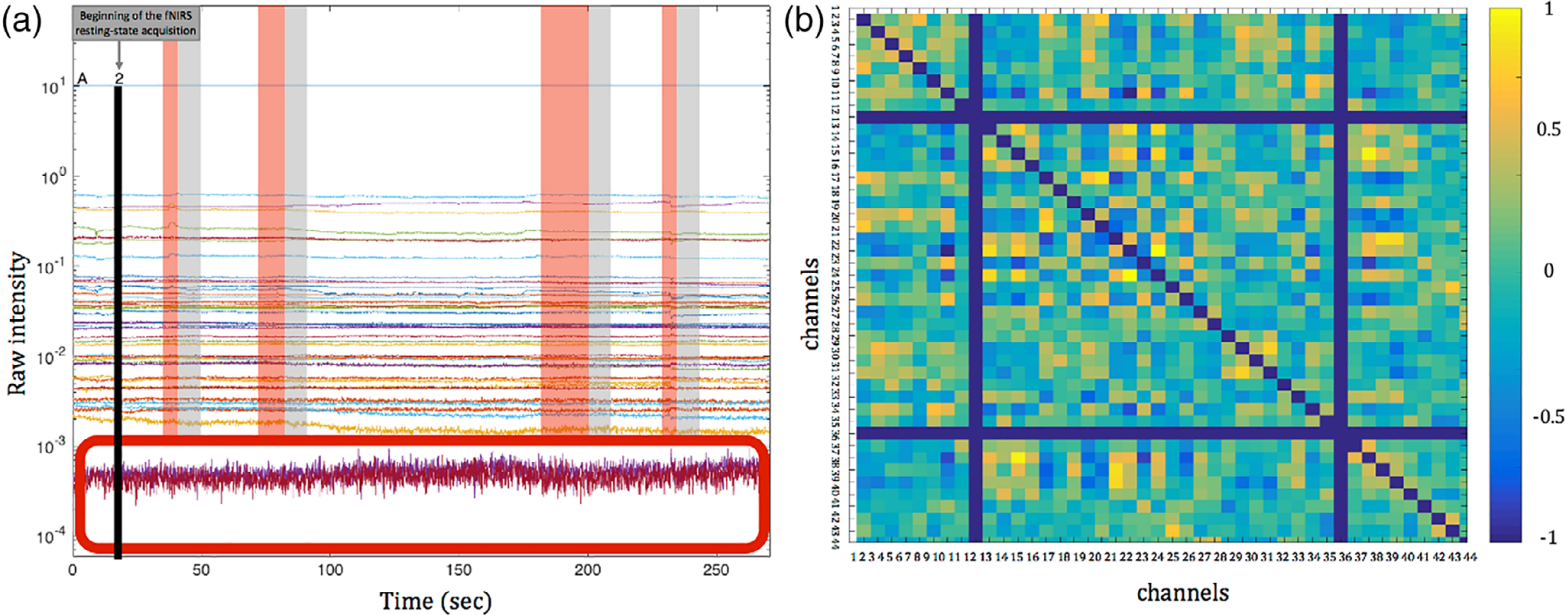
(a) Representative segment of the resting‐state raw data acquired. In the lower part of the figure, a red box marks channels that were excluded from the analysis because the mean intensity was lower than 10^−3^. On the remaining channels, red windows mark chunks of excluded data based on the behavioural coding. The grey windows represent the 8 s of additional data that was excluded after each invalid section. (b) Correlation matrix of 44 × 44 channels (Fisher z‐transformed rho values). Blue lines indicate channels that were excluded because of the pre‐processing (the diagonal blue line indicates the correlations of the channels with themselves). Figure 5 has been reproduced with modification from Bulgarelli, et al. 2019

Data analysis were carried out in MATLAB (MathWorks, Natick, MA). fNIRS resting‐state data were extracted for each participant from all the channels and channels with mean intensity lower than 10^−3^ μmol were excluded as such low intensity values indicate bad optode‐scalp coupling (Figure 5a). The global mean removal is a step which might be implemented in resting‐state adult analysis. One of the most common method to perform it is the implementation of short‐separation channels on the fNIRS cap (Brigadoi & Cooper, 2015; Gagnon et al., 2012). However, a recent infant study showed no differences between channels activations detected with and without the short‐separation channels on 6‐month‐olds (Emberson, Crosswhite, Goodwin, Berger, & Aslin, 2016). Consistent with this, we have decided not to include this step in our pre‐processing of the data.

Videos of the testing session were coded offline and periods where the infant moved, cried, or looked at something socially engaging (e.g., the mum or the experimenter) were marked as invalid, as well as periods during which the mum or experimenter were talking. To assess inter‐coder reliability, 20% of the videos at every visit were blindly double‐coded by another researcher. We found high reliability between the two coders (11 months, *k* = 0.78; 18 months, *k* = 0.84, 24 months, *k* = 0.85; 30 months, *k* = 0.89, 36 months, *k* = 0.80).

As it takes at least 8 s for the infant HRF to return to baseline levels (Lloyd‐Fox et al., 2010; Taga, Watanabe, & Homae, 2011), 8 s of consecutive data across all the channels were excluded after each invalid section, to ensure that we were only including periods of resting state (Figure 5a). Sections of good data were included only if they were at least 5 s long (uninterrupted). After behavioural coding, time series for each fNIRS channel were extracted for each participant and only participants who had at least 100 s of clean data^4^ in total, and less than 30% of the channels excluded were considered for further analysis. The light attenuation values were band‐pass filtered (0.01–0.08) and converted to relative concentrations of haemoglobin using the modified Beer–Lambert law (Villringer & Chance, 1997). Differential path length factors were adapted to ages: 11 months = 5.13; 18 months = 5.20; 24 months = 5.25; 30 months = 5.27; 36 months = 5.30 (calculated based on Scholkmann & Wolf, 2013). For each participant, the correlation matrix between all the channels that survived pre‐processing was calculated for both HbO_2_ and HHb, resulting in a 30 × 30 or 44 × 44 matrix of channels. We then applied a Fisher z‐score transformation on the correlation matrix for further statistical analyses (Figure 5b).

Each functional connection between channels belonging to the frontal region and the temporoparietal region was inserted as a dependent variable in a linear mixed model (Verbeke & Molenberghs, 2000). Like previous infants studies (Grossmann, Cross, Ticini, & Daum, 2013; Lloyd‐Fox et al., 2010; Lloyd‐Fox, Széplaki‐Köllod, Yin, & Csibra, 2015; Southgate, Begus, Lloyd‐Fox, di Gangi, & Hamilton, 2014), we focused this analysis on the HbO_2_ signal. Compared to repeated measures ANOVA, linear mixed models account for within person dependence and allow for there to be missing data by using only information from the individual at the other visits (Field, Miles, & Field, 2012; Gad & Youssif, 2004). The linear mixed model for a dependent variable ‘*y*’, of the participant ‘*p*’, at a specific time point ‘*t*’, is:

$$y_{\mathrm{pt}}={\text{Intercept}}_{p}+d_{p}+{\text{βAge}}_{\mathrm{pt}}+\varepsilon_{\mathrm{pt}}$$

in which Age_pt_ is the age of the *p*th participant at the *t*th time point (visit). The dependent variable, that is, the functional connectivity, was modelled here as a function age (βAge) with a random participant effect (*d*_p_) and errors (ε_pt_). Intercept and age were fixed effects, while within participant dependence (*d*_p_) was modelled as a random effect. This same procedure was used in other longitudinal studies that explored brain connectivity changes over time (Wierenga et al., 2018). The linear mixed model included the 32 participants who had valid data for at least two visits. The type of covariance between the observations was specified as Autoregression (AR) as two measures close in time of the same participant are likely to be correlated (Selig & Little, 2012). To ensure statistical reliability, significant results from the linear mixed model were corrected for multiple comparisons using the False Discovery Rate (FDR) method (Benjamini & Hochberg, 1995; Singh & Dan, 2006).

Supporting Information show supplementary analyses between age groups, performed with paired *t*‐tests. Pairs of functional connections were included in the analysis only if at least half of the sample contributed data to the statistical tests (Tables S2–S11).

## 3 RESULTS

### 3.1 Functional connectivity per age group

**Figure 6:**
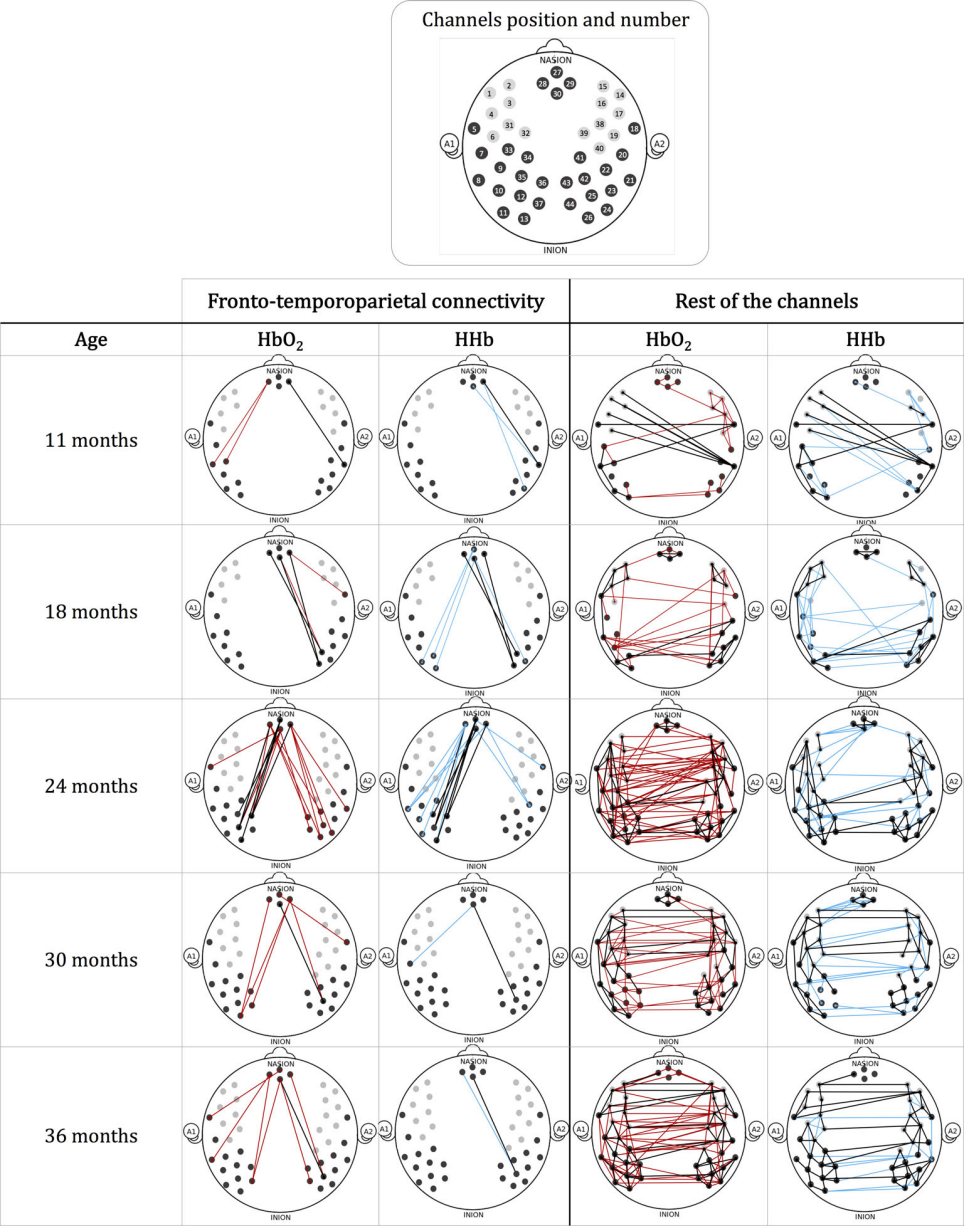
Graphical representations of the functional connections that are significantly different from zero, both in the frontotemporoparietal regions and in the rest of the channels. HbO_2_ is plotted in red and HHb is plotted in blue. Connections that are significantly different from zero both in the HbO_2_ and the HHb signals are plotted in black. *N* indicates the number of included participants at each visit

Previous research with adults has explored the relationship between HbO_2_ and HHb in fNIRS resting‐state data, and has revealed a comparable pattern of spontaneous fluctuations of the two signals (Lu et al., 2010; Sasai, Homae, Watanabe, & Taga, 2011; White et al., 2009). Therefore, prior to any further analyses, we investigated the consistency of the connectivity patterns between the HbO_2_ and HHb signals, with one sample *t*‐tests on the Fisher‐transformed correlation coefficients on both signals for each age group. Significant functional connections within the rest of channels were also plotted to assess whether differences in the connectivity between ages were limited to the fronto‐temporoparietal areas or whether they were also present over the other channels. Figure 6 shows functional connections that are significantly different from zero in the HbO_2_ signal (red lines), in the HHb signal (blue lines) and in both signals (black lines) for each age group, revealing similar patterns in terms of location and number of connections between the two chromophores. At 11 months, 1 out of 3 connections in the HHb signal overlap with those in the HbO2 signal within the fronto‐temporoparietal regions, and 8 out of 21 connections in the HHb signal overlap with those in the HbO2 signal within the rest of the channels. At 18 months, 6 out of 17 connections in the HHb signal overlap with those in the HbO2 signal within the fronto‐temporoparietal regions, and 23 out of 53 connections in the HHb signal overlap with those in the HbO2 signal within the rest of the channels. At 24 months, 3 out of 7 connections in the HHb signal overlap with those in the HbO2 signal within the fronto‐temporoparietal regions, and 51 out of 89 connections in the HHb signal overlap with those in the HbO2 signal within the rest of the channels. At 30 months, 1 out of 2 connections in the HHb signal overlap with those in the HbO2 signal within the fronto‐temporoparietal regions, and 51 out of 86 connections in the HHb signal overlap with those in the HbO2 signal within the rest of the channels. At 36 months, 1 out of 2 connections in the HHb signal overlap with those in the HbO2 signal within the fronto‐temporoparietal regions, and 62 out of 85 connections in the HHb signal overlap with those in the HbO2 signal within the rest of the channels. The overlap between connections significant in both chromophores increased with age.

**Figure 7:**
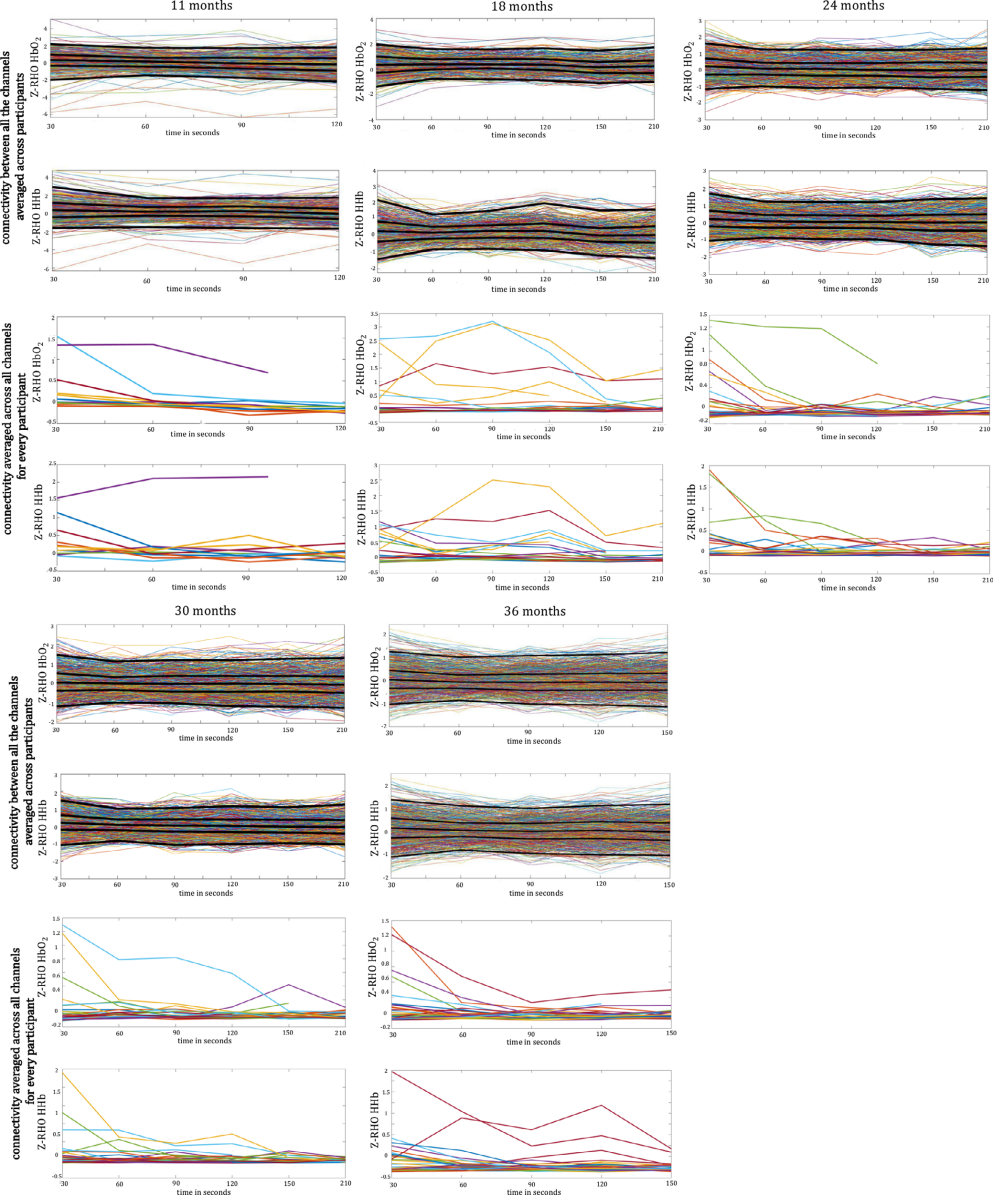
Connectivity estimation over time. For every age, the first two lines of the plot show connectivity between all the channels averaged across participants in HbO_2_ and HHb. Black lines indicate different percentiles (2.5th, 25th, 50th 75th, 97.5th from bottom to top). The second two lines of plot show connectivity averaged across all channels per participant in HbO_2_ and HHb. In every plot, *x*‐axes indicate time of recording included in the analysis, and the *y*‐axes indicate RHO values of connectivity fisher z‐transformed

In order to assess whether the data included in the analyses per each age group are sufficient for a reliable and stable estimation of functional connectivity, we assessed whether connectivity values between all the channels of our matrix reach stability over the time included in the analysis (Gordon et al., 2017; Figure 7). As expected, there seems to be a lot of variability at the beginning of the recording, while connectivity reaches a more robust estimate with longer recordings. At every visit, connectivity shows stable pattern after about 60–90 s of data.

### 3.2 Linear mixed model

To estimate how fronto‐temporoparietal functional connectivity changes over the five visits, we analysed the fronto‐temporoparietal connections in the HbO_2_ signal using a linear mixed model.

**Figure 8:**
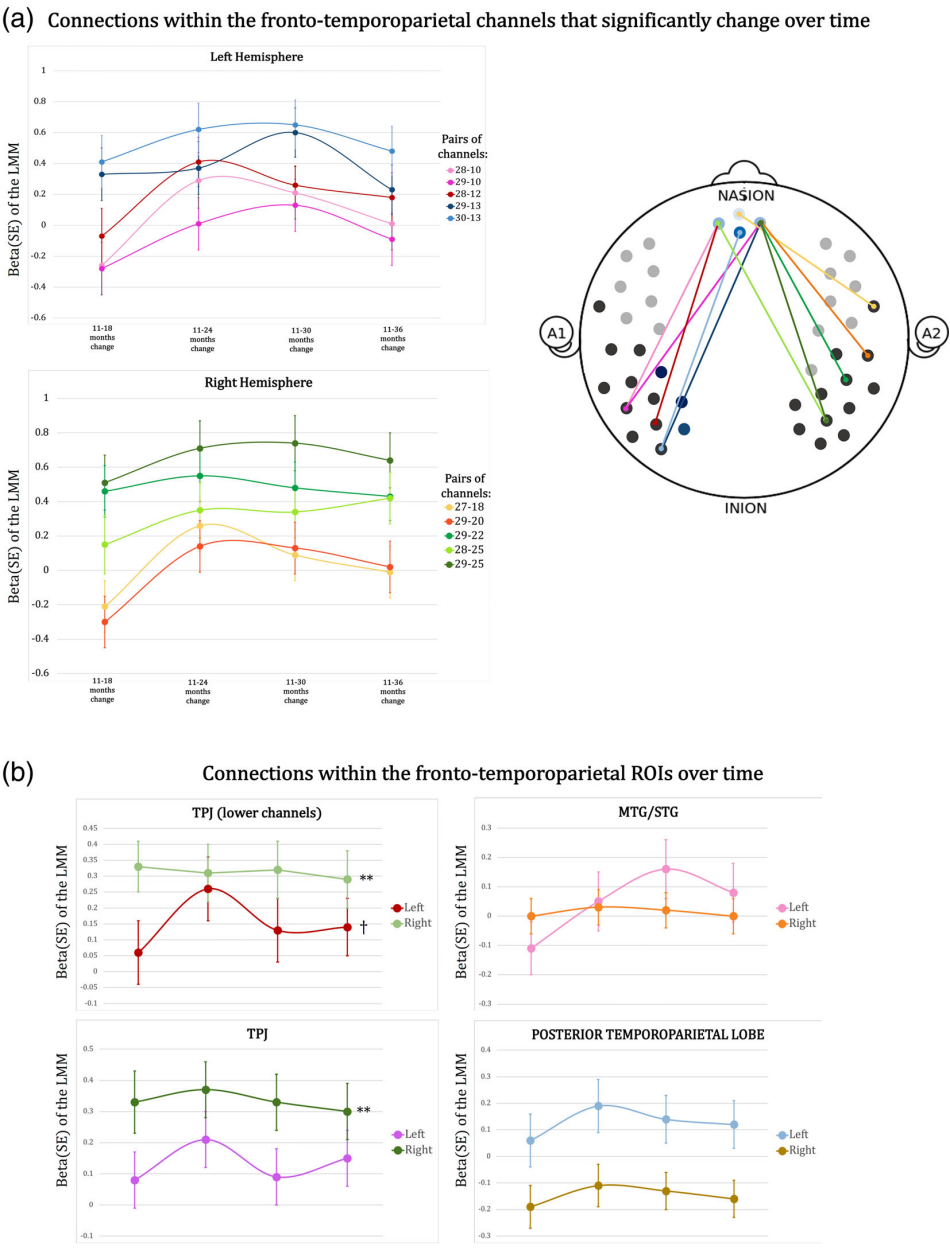
(a) Functional connections that showed a significant change over time within the fronto‐temporoparietal channels. (b) Graphical representation of the changes over time of the connections within the fronto‐temporoparietal ROIs. **p < .05 that survived the FDR correction for multiple comparisons, *p < .05; ^†^ *p* < .065

Table 5 shows the connections within fronto‐temporoparietal channels and ROIs that showed statistically significant changes over time. Results in the left part of the table show whether there is a significant change in functional connectivity within the five visits. The beta values for the 11th month time point represent the connectivity values estimated by the random effects and the p‐value at this age point shows the significance of this beta compared to zero. Betas at the following visits represent changes in connectivity compared with the 11th month visit (right part of the table). Figure 8 shows a graphical representation of the functional connections that showed a significant change over time within the fronto‐temporoparietal channels (Figure 8a) and within the fronto‐temporoparietal ROIs (Figure 8b).

**Table 5:** Significant and marginally significant changes of the connections within the fronto‐temporoparietal channels and changes of the connections between all the ROIs over time

Linear mixed model between channels									
Temporoparietal ROI	Temporoparietal channels	mPFC channels	*F*	*p*	Baseline (11 months)	11–18 months change	11–24 months change	11–30 months change	11–36 months change
					Beta (SE), *p*	Beta (SE), *p*	Beta (SE), *p*	Beta (SE), *p*	Beta (SE), *p*
Left/middle temporal gyrus	10	28	4.18	.004**	−0.06 (0.15), .672	−0.26 (0.19), .180	0.29 (0.18), .116	0.21 (0.17), .220	0.01 (0.17), .965
		29	2.74	.035*	−0.05 (0.14), .733	−0.28 (0.17), .111	0.01 (0.17), .052***	0.13 (0.17), .451	−0.09 (0.17), .593
	12	28	3.24	.016**	−0.12 (0.14), .391	−0.07 (0.18), .679	0.41 (0.16), .018*	0.26 (0.16), .124	0.18 (0.16), .258
	13	29	3.97	.006**	−0.36 (0.15), .024*	0.33 (0.18), .083	0.37 (0.18), .045*	0.60 (0.18), .001*	0.23 (0.17), .184
	13	30	4.33	.004**	−0.55 (0.14), .001*	0.41 (0.17), .020*	0.62 (0.17), .001*	0.65 (0.16), .001*	0.48 (0.16), .004*
Right STG	18	27	3.09	.021*	0.15 (0.13), .276	−0.21 (0.17), .221	0.26 (0.16), .119	0.09 (0.16), .540	−0.01 (0.15), .973
	20	29	4.04	.005**	−0.03 (0.12), .788	−0.30 (0.15), .052***	0.14 (0.15), .363	0.13 (0.15), .391	0.02 (0.15), .873
Right TPJ	22	29	3.46	.012**	−0.50 (0.12), 0.001*	0.46 (0.15), .003*	0.55 (0.15), .001*	0.48 (0.15), .002*	0.43 (0.14), .005*
	25	28	2.98	.023**	−0.31 (0.13), .023*	0.15 (0.17), .381	0.35 (0.16), .028*	0.34 (0.15), .029*	0.42 (0.15), .007*
		29	5.50	.004**	−0.46 (0.14), .002*	0.51 (0.16), .003*	0.71 (0.16), .001*	0.74 (0.16), .001*	0.64 (0.16), .001*

Linear mixed model between ROIs							
Temporoparietal ROI	*F*	*p*	Baseline (11 months)	11–18 months change	11–24 months change	11–30 months change	11–36 months change
			Beta (SE), *p*	Beta (SE), *p*	Beta (SE), *p*	Beta (SE), *p*	Beta (SE), *p*
Left MTG/STG	1.37	.249	−0.02 (0.08), .755	−0.11 (0.10), .312	−0.05 (0.10), .595	0.16 (0.10), .114	0.08 (0.09), .408
Right MTG/STG	0.25	.909	−0.07 (0.05), .219	−0.01 (0.06), .963	0.03 (0.06), .561	0.02 (0.06), .731	−0.01 (0.06), .985
Left posterior temporoparietal lobe	1.19	.319	−0.14 (0.08), .097	0.06 (0.10), .524	0.19 (0.10), .060***	0.14 (0.09), .145	0.12 (0.09), .186
Right posterior temporoparietal lobe	1.39	.244	0.14 (0.06), .037*	−0.19 (0.08), .031*	−0.11 (0.08), .148	−0.13 (0.07), .101	−0.16 (0.07), .041*
Left TPJ (only channels 9, 12)	2.35	.062***	−0.21 (0.08), .015*	0.06 (0.10), .538	0.26 (0.10), .011*	0.13 (0.10), .174	0.014 (0.09), .137
Right TPJ (only channels 22, 25)	3.86	.006**	−0.34 (0.07), .001*	0.33 (0.09), .001*	0.31 (0.09), .001*	0.32 (0.09), .001*	0.29 (0.08), .011*
Left TPJ	1.46	.222	−0.24 (0.08), .004*	0.08 (0.10), .459	0.21 (0.09), .033*	0.09 (0.09), .313	0.15 (0.09), .096
Right TPJ	1.31	.004**	−0.34 (0.07), .001	0.33 (0.09), .001*	0.37 (0.09), .001*	0.33 (0.05), .001*	0.30 (0.90), .001*

*Note*: Results are displayed in terms of estimated betas, standard errors (SE), and *p* values. *p < .05; **p < .05 that survived the FDR correction for multiple comparisons; ***p < .065.

To increase the power of our analysis and reduce the number of multiple comparisons, we performed the linear mixed model on the ROIs as well. To do this, Fisher‐transformed correlation coefficients were averaged across the channels of the ROIs defined in section 2.4. As the TPJ is covered by channels of the 44‐channel configuration which were not used to acquire data at 11 and 18 months, connectivity between the frontal cortex and the TPJ was estimated by considering two definitions of this region: (a) the TPJ channels used at every visit (channels 9 and 12 for the left TPJ and channels 22 and 25 for the right TPJ); (b) all the channels belonging to the TPJ region.

Channels belonging to the temporoparietal regions that showed statistically significant functional connections with the frontal cortex can be clustered into two main regions, the left posterior temporal lobe (channels 13), the left and right STG/MTG (channels 10, 18, 20), the left and right TPJ (channels 12, 22, 25) (see coregistration in Table 4). Among the 10 functional connections that showed a statistically significant change over time, only two did not survive the FDR correction for multiple comparisons (channel 27–channel 18 and channel 29–channel 10). As can be seen in Table 5 and in Figure 8a, the greatest increase in fronto‐temporoparietal connectivity was at 24 months for 5 out of the 10 connections that significantly changed with time (pairs of channels: 28–10, 28–12, 27–18, 29–20, 29–22), at 30 months for 4 out of the 10 connections that significantly changed with time (pairs of channels: 29–10, 29–13, 30–13, 29–25), while only one fronto‐temporoparietal connection reached the maximum increase at 36 months (pair of channels 28–25).

Regarding the linear mixed model performed on the ROIs, there was a statistically significant change with time in the mPFC‐right TPJ functional connection—both when considering only channels 22 and 25 and also when considering the additional channels added from 24 months—with a maximum peak respectively at 18 and 24 months (Table 5 and Figure 8b). Both of these survived the FDR correction for multiple comparisons. There was also a marginal significant change in time in the mPFC‐left TPJ connection (only channels 9 and 12), with a maximum peak at 24 months.

### 3.3 Changes in connectivity outside the DMN over time

**Figure 9:**
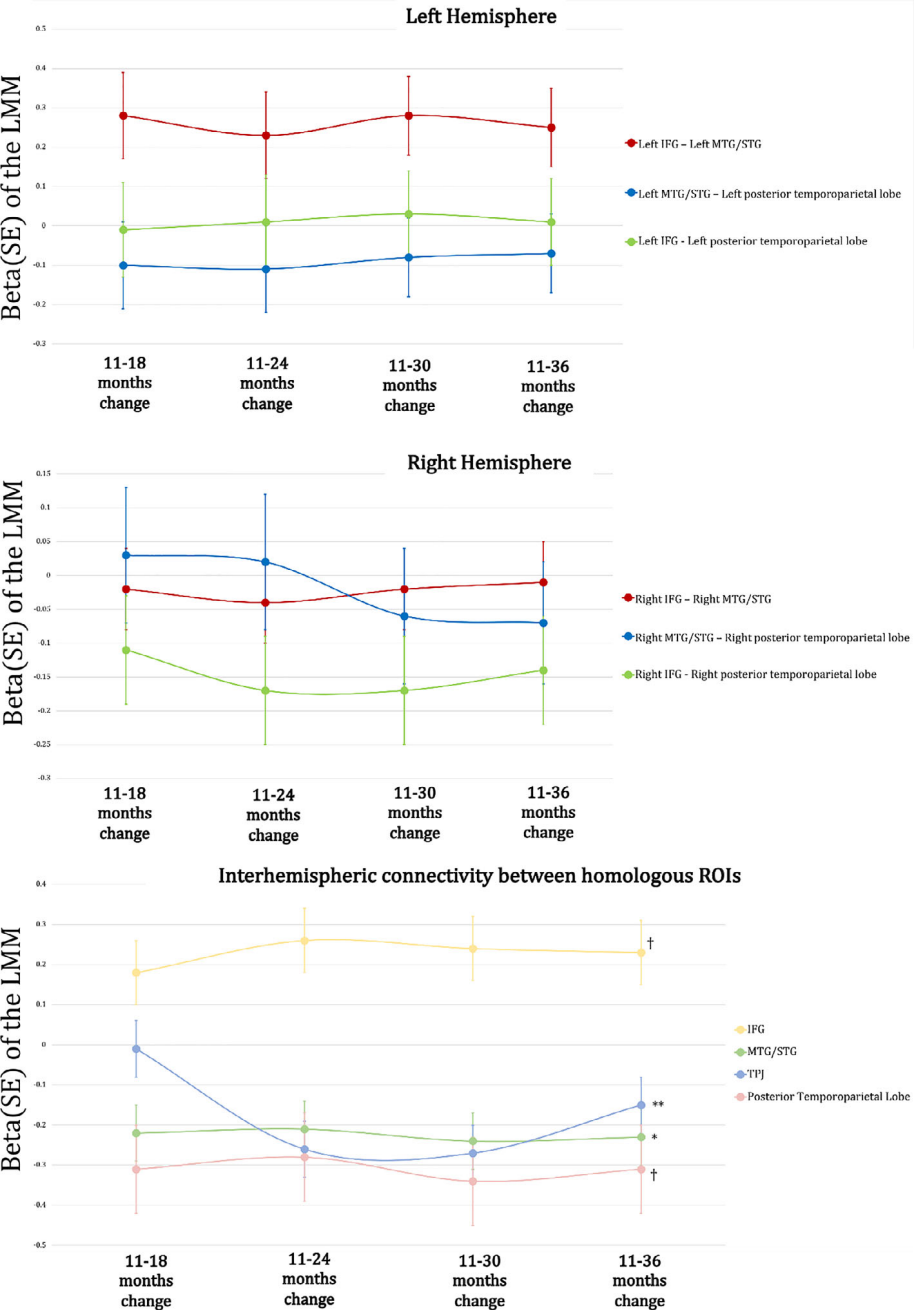
Functional connections outside the DMN that showed a significant change over time (left and right hemisphere) and interhemispheric connections between homologous regions. **p < .05 that survived the FDR correction for multiple comparisons, *p < .05; ^†^ *p* < .065

In order to assess whether the longitudinal variations that we observed in regions belonging to the DMN characterised this network only or were related to the rest of cortex as well, we assessed longitudinal changes intrahemispherically between IFG, MTG/STG and posterior temporoparietal lobe and interhemispherically between homologous regions (left and right IFG, left and right MTG/STG, left and right TPJ, left and right posterior temporoparietal lobe) using the linear mixed model. Figure 9 and Table 6 show the results.

**Table 6:** Changes of the connections outside the DMN and interhemispheric connectivity over time

	*F*	*p*	Baseline (11 months)	11–18 months change	11–24 months change	11–30 months change	11–36 months change
			Beta (SE), *p*	Beta (SE), *p*	Beta (SE), *p*	Beta (SE), *p*	Beta (SE), *p*
*Linear mixed model—Connectivity outside the DMN*							
Temporoparietal ROI							
Left IFG–Left MTG/STG	1.94	.112	−0.18 (0.09), .060***	0.28 (0.11), .013*	0.23 (0.11), .039*	0.28 (0.10), .011*	0.25 (0.10), .018*
Left MTG/STG–Left posterior temporoparietal lobe	0.32	.862	0.12 (0.09), .219	−0.10 (0.11), 0.329	−0.11 (0.11), .311	−0.08 (0.10), .421	−0.07 (0.10), .507
Left IFG–left posterior temporoparietal lobe	0.10	.980	−0.04 (0.10), .683	−0.01 (0.12), .894	0.01 (0.12), .007*	0.03 (0.11), .305	0.01 (0.11), .881
Right IFG–Right MTG/STG	0.176	.950	0.06 (0.05), .249	−0.02 (0.06), .687	−0.04 (0.06), .543	−0.02 (0.06), .675	−0.01 (0.06), .906
Right MTG/STG–Right posterior temporoparietal lobe	1.14	.343	−0.07 (0.09), .385	0.03 (0.10), .760	0.02 (0.10), .797	−0.06 (0.10), .544	−0.07 (0.09), .472
Right IFG–right posterior temporoparietal lobe	1.06	.380	0.07 (0.08), .336	−0.11 (0.08), .183	−0.17 (0.08), .060***	−0.17 (0.08), .056***	−0.14 (0.08), .093
*Linear mixed model—Interhemispheric connectivity*							
ROI							
IFG	2.36	.062***	−0.18 (0.07), .017*	0.18 (0.08), .043*	0.26 (0.09), .005*	0.24 (0.08), .006*	0.23 (0.08), .008*
MTG/STG	3.09	.020**	0.20 (0.06), .002*	−0.22 (0.07), .006*	−0.21 (0.07), .007*	−0.24 (0.07), .001*	−0.23 (0.07), .002*
TPJ	7.30	.001**	0.23 (0.06), .001*	−0.01 (0.08), .918	−0.26 (0.08), .002*	−0.27 (0.07), .001*	−0.15 (0.07), .055***
Posterior temporoparietal lobe	2.32	.066***	0.39 (0.10), .001*	−0.31 (0.11), .008*	−0.28 (0. 11), .017*	−0.34 (0. 11), .004*	−0.31 (0. 11), .008*

*Note*: Results are displayed in terms of estimated betas, standard errors (SE), and *p* values. *p < .05; **p < .05 that survived the FDR correction for multiple comparisons; ***p < .065.

None of the intrahemispheric connections showed a significant main effect of time, even though connectivity between IFG and left MTG/STG in the left hemisphere showed positive significant changes at 18, 24, 30, 36 months compared to 11 months. Interhemispheric connectivity showed a significant change over time, but mainly due to changes between 11 months and later visits, as beta values indicating changes between 18, 24, 30, 36 months and 11 months are very similar. After the first year of life interhemispheric connectivity seems to be relatively stable without significant changes.

## 4 DISCUSSION

The DMN is a resting‐state network that has been extensively studied in adults, but knowledge of its development is limited. In contrast to other primary sensory resting‐state networks that are present from birth to support basic sensorimotor functions, recent studies suggest that the DMN develops gradually over the first years of life. However, most of the information on the development of the DMN comes from fMRI studies with sleeping infants. This might limit our understanding of the developmental trajectory of this network, as sleep stages can affect functional connectivity estimates (Horovitz et al., 2009; Mitra et al., 2017; Tagliazucchi & Laufs, 2014). Therefore, the use of fNIRS in awake infants has the potential of contributing to this investigation. In the current study, we used fNIRS to explore the developmental trajectory of fronto‐temporoparietal connectivity—as a proxy of the DMN—in awake infants at five time points. We were able to acquire resting‐state data in awake infants and toddlers, under conditions that are more similar to those typically used in adult studies, and we validated an analysis pipeline that can be applied in future studies. At every visit, we coregistered the fNIRS optodes on an MRI template of the same age. This allows us to more precisely estimate channels‐brain correspondence and to adjust the channels‐ROIs correspondence at every age, accounting for brain growth.

In line with previous resting state fNIRS studies (Lu et al., 2010; Sasai et al., 2011; White et al., 2009), the one sample *t*‐tests at each visit on the HbO_2_ and the HHb signals showed some consistency of the connectivity patterns that were measured in the two chromophores, suggesting that the data was reliable. Additionally, at every age connectivity reached stability in most of the infants after about 60–90 s of data included, which is consistent with a recent study on children showed that as little as 1 min of resting‐state fNIRS recording is sufficient to obtain accurate functional connectivity estimation (Wang, Dong, & Niu, 2017).

### 4.1 Changes in the fronto‐temporoparietal functional connectivity over time

Results from the linear mixed model analysis between the fronto‐temporoparietal channels and between fronto‐temporoparietal ROIs showed stronger fronto‐temporoparietal connections at older ages compared to younger ages, consistent with previous studies that have found a gradual increase of DMN connectivity over the first years of life (Damaraju, Caprihan, Lowe, et al., 2014; Gao et al., 2009). Results showed a maximum increase of the functional connections at 24 and 30 months compared to the 11th month visit. One may think that the maximum peak of the functional connectivity change at 24 and 30 months could be related to methodological aspects, such as a higher level of noise in the data at these ages, as a high level of movement during the resting‐state acquisition would have most likely led to a spurious increase in functional connectivity (Deen & Pelphrey, 2012; Power, Barnes, Snyder, Schlaggar, & Petersen, 2012; van Dijk, Sabuncu, & Buckner, 2012). However, we took great care to remove sections of the data affected by motion artefacts during the pre‐processing. When the infants were 11 months, they provided the noisiest dataset, and the quality of the resting‐state recordings increased with age. More likely, the maximum increase of the functional connections at 24 and 30 months compared to the 11 month visit indicates a stability in the strength of the connectivity in DMN regions, which is consistent with a previous study by Gao et al. (2009), that showed that by 2 years, the DMN is functionally similar to the DMN observed in adults, with long‐range connections between the frontal cortex and the posterior regions of the DMN (Gao et al., 2009, 2016). In this study, we reported major changes in DMN connectivity between the first and the second year of life, which is consistent with the remarkable increments in white matter tracts connecting core hubs of the DMN that have been documented within this period (Fan et al., 2011). However, Gao et al. (2009, 2015), showed limited variations in the DMN functional connectivity within this age when using fMRI with sleeping infants. The DMN connectivity estimated at 24 months similar to the network observed in adults is mainly driven by changes up to the first year of life, followed by minor increases between the first and the second year (Gao et al., 2009, 2015). One may wonder whether these dissimilarities are due to differences in arousal states of the participants. It is difficult to draw clear comparisons here, as we have not collected resting‐state data from infants younger than 1 year of age. Future research could assess functional connectivity between DMN regions in very young awake infants using fNIRS, as we have done in this study. This would allow us to better understand whether the major variations in DMN connectivity reported in different periods in the current work and previous studies (Gao et al., 2009, 2015) can be explained by the effect of sleep on functional connectivity or there are additional reasons.

The peak in fronto‐temporoparietal connectivity at 24–30 months seems to be followed by a decrease. This nonlinear development could be explained by pruning processes, that is, the removal of redundant connections (Huttenlocher, Vasilyeva, & Shimpi, 2004), resulting in a more efficient set of connections (Thompson et al., 2005) and enabling the reorganisation of functional networks (Gao et al., 2016; Levitt, 2003). Pruning is known to be a region‐specific process, affecting different brain regions at different stages of the development (Casey, Tottenham, Liston, & Durston, 2005). The increase in connections and the subsequent pruning happens last in the frontal lobe, while this process affects other regions such as the auditory, the visual and the sensorimotor cortex at an earlier age (Huttenlocher & Dabholkar, 1997). Consistent with our findings, it has been shown that a peak in synaptic density in the frontal cortex is achieved only after the first year of life (Huttenlocher & Dabholkar, 1997; Tierney & Nelson, 2009) with pruning of frontal connections starting at around 2 years (Casey et al., 2005; Kolb & Gibb, 2011). Patterns of decreases following increases in the maturity of the brain have been observed not only in relation to functional connectivity. Other works documented the same non‐linear growth in cortical thickness (Shaw et al., 2008), in some white matter tracts (Mukherjee et al., 2001), and in grey matter density (Sowell, Thompson, & Toga, 2004; Toga, Thompson, & Sowell, 2006), findings which are consistent with a reorganisation process in the brain after its growth for a more efficient activity. However, why we observed this non‐linear growth particularly in the fronto‐temporoparietal network rather than in the connections outside the DMN is unclear. In this respect, it is important to point out that most of the previous longitudinal studies acquired resting‐state data up to 2 years, or with intervals not as frequent as 6 months (Damaraju, Caprihan, & Lowe, 2014; Gao et al., 2009; Homae et al., 2010), or from 6 to 7 years of age to adulthood (Jolles, Van Buchem, Crone, & Rombouts, 2011; Marusak et al., 2017; Supekar et al., 2010; Supekar, Musen, & Menon, 2009) where fMRI is suitable method for awake participants. There still seems to be a lack of investigation of changes in the DMN (and in functional networks in general) after the second year of life until childhood. For this purpose, we showed in this work that fNIRS is a valid method to be used with awake toddlers and more studies are needed to investigate networks development using fNIRS. This is the first study that explored the development of fronto‐temporoparietal connections up to 3 years of age at frequent intervals, and it would be beneficial if these results would be replicated in an independent sample and extended till childhood.

Consistent with results from the linear mixed model, the paired *t*‐tests presented in the Supporting Information showed stronger fronto‐temporoparietal connections at older ages compared to younger ages, and a peak of fronto‐temporoparietal connections at 24 months in the HbO_2_ signal, as the participants showed the same number of fronto‐temporoparietal connections that increased and that decreased between 24, 30 and 36 months in the HbO_2_ signal. In the HHb signal, the fronto‐temporoparietal connections increased up to 30 months and, while there were no differences between 24 and 30 months, these connections increased again between 30 and 36 months. Among the paired sample *t*‐tests presented in the Supporting Information, the limited number of the participants that contributed to the paired sample *t*‐tests represents the main limitation. It is likely that the comparisons between two time points were underpowered and results should be interpreted with caution. Nevertheless, most of the linear mixed models survived the FDR correction for multiple comparison, which allows more confidence in interpreting the results.

A gradual increase in fronto‐temporoparietal connectivity until to 24 months of age, particularly significant in the right hemisphere, is consistent with the hypothesised relationship between the DMN and self‐processing (Buckner & Carroll, 2007; Golland et al., 2008; Qin & Northoff, 2011). The study of the development of the sense of self has recently been a topic of much interest in developmental psychology and whether we currently have appropriate measurements for its assessment is under debate. The sense of self is thought to develop between 18 and 24 months of age (Amsterdam, 1972; Rochat, 2003), and it is typically assessed using the mirror self‐recognition task (Amsterdam, 1972). However, there is no general consensus on the significance of mirror self‐recognition (for some criticisms, see Heyes & Swartz, 1997; Mitchell, 1993). In another study, we showed that connectivity between regions belonging to the DMN is greater in 18‐month‐olds who show evidence of mirror self‐recognition compared to those who do not (Bulgarelli et al., 2019). Interestingly, the mPFC‐right TPJ connectivity change over time with a peak at 18 and 24 months found in the current work is in line with our previous findings.

Previous studies have demonstrated an overlap between certain regions of the DMN and two other networks, the fronto‐parietal network and the dorsolateral attention network, both related to cognitive control (Spreng, Stevens, Chamberlain, Gilmore, & Schacter, 2010; Tomasi & Volkow, 2011), therefore some of the results could reflect developmental changes also in these two networks. However, these interpretations are limited by the fact that in the current work we did not record from the dorsolateral prefrontal cortex, which is a core hub of the fronto‐parietal network and the dorsolateral attention network. Although in some infants, the lateral channels of the frontal array may have detected activity from a more lateral portion of the frontal cortex than the mPFC, future studies in which the fNIRS array is specifically designed to measure from dorsolateral prefrontal cortex are needed to investigate the development of connectivity in these cognitive control networks more accurately.

### 4.2 Changes in the functional connectivity outside the DMN over time

To verify whether the significant longitudinal changes detected within the DMN regions were specific to this network or whether similar changes can be observed in the whole cortex, we assessed connectivity outside the DMN and interhemispherically between homologous regions. Results seem to be consistent with the first hypothesis, suggesting that the significant increase in connectivity until 24/30 months characterises regions belonging to the DMN only, as connections outside this network seem to not show any significant changes over time. The only exception to this is the significant increment of the left IFG‐left STG/MTG connectivity between 11 months and the following ages. This might be consistent with previous preliminary findings showing that the left IFG might be a core hub in resting‐state connectivity development over the first years of life (Homae, Watanabe, & Taga, 2016).

Interhemispheric connectivity between homologous regions seems to significantly change after the first year of life, and then reaches stability. These results seem to suggest that the pattern of longitudinal changes observed in the DMN is specific to this network. However, as the fNIRS method only allows us to measure the cortical surface, and our optodes did not cover the entire head, we acknowledge that these additional analyses do not allow us to completely rule out potential changes in general brain maturation accounting for some of the developmental change we observed in the DMN. The only way to correctly assess change in connectivity in the entire brain would be to acquire MRI images. One might think that the presence of interhemispheric connectivity as early as 11 months is in contrast with the literature about the protracted development of the corpus collosum (Pujol, Vendrell, Junqué, Martí‐Vilalta, & Capdevila, 1993). However, some white matter tracts in the corpus callosum are present right after birth, and interhemispheric connectivity before the first year of life has been documented elsewhere (Gao et al., 2009; Homae et al., 2010; Keehn, Wagner, Tager‐Flusberg, & Nelson, 2013; Perani et al., 2011; Smyser, Snyder, & Neil, 2011; Taga et al., 2011). The corpus collosum undergoes a slow continuous development from infancy until early adulthood (for example see Chavarria, Sánchez, Chou, Thompson, & Luders, 2014; Giedd et al., 1996, 1999; Giorgio et al., 2010; Hinkley et al., 2012; Keshavan et al., 2002; Luders, Thompson, & Toga, 2010), but this does not mean that interhemispheric connectivity is not present before the corpus callosum is completely mature.

### 4.3 Methodological limitations and further considerations

It may be interesting to notice that the main reason for exclusion from the analysis changed over time. While participants at younger ages were mainly excluded because their artefact‐free resting‐state data did not reach the minimum required length or because they refused to wear the fNIRS cap, at older ages the main reason for exclusion was the high number of channels with poor light intensity. The difficulty for young infants to reach a quiet state (especially after having already been presented with several other experiments) significantly reduced amount of data available for analysis at the 11th month visit compared with the ones, limiting the longitudinal comparisons.

In addition, testing participants at different visits with different fNIRS array configurations restricted the comparisons of some of the fronto‐temporoparietal connections between all the visits. Although the 44‐channel configuration was an extension of the 30‐channel configuration, comparisons at 11 and 18 months were limited by the absence of the additional channels. The reason for adding the additional channels to the 30‐channel configuration was to improve the detection of TPJ spontaneous fluctuations, one of the core regions for understanding developmental changes in the fronto‐temporoparietal connections. However, it is important to highlight that changes over time between the mPFC—right TPJ connectivity showed the same significant pattern when considering the TPJ channels belonging to 30‐channel configuration only, and when we consider those added in the 44‐channel configuration, suggesting that this result is not driven by the extension of the spatial coverage the right TPJ region.

At every visit, we have co‐registered the NIRS array of a subset of participants with MRI scans, but we do acknowledge that we have not accounted for inter‐individual differences of each participant at each age point. However, we have excluded every infant with poor position of the NIRS headband/cap, based on the pictures taken during the testing sessions. Without individual MRI scans, it is difficult to estimate for each participant whether we measure from exactly the same area, which is one of the main limitations of fNIRS. We believe that by taking a representative sample of infants and doing the co‐registration based on MRI templates that closely matched their head shape and size, the ROIs at every visit were likely to be accurate for the majority of participants.

In the current study, we measured the development of fronto‐temporoparietal connectivity while the participants watched a screen‐saver like video. Although the use of non‐social videos to measure resting state connectivity has been previously validated with children and adults (Müller et al., 2015; Vanderwal et al., 2015; Xiao et al., 2016), and consistency in functional connectivity estimated during non‐social videos and rest has been documented (Finn et al., 2017; Vanderwal et al., 2015), we are aware that the presence of audio‐visual stimuli does not entirely equate the testing conditions of resting‐state studies historically performed with adults. However, the use of this screensaver video was the only feasible way to measure resting state connectivity with infants and toddlers while they were awake. In fact, given that sleep stages (Mitra et al., 2017; Tagliazucchi & Laufs, 2014) and movement (Power et al., 2012; van Dijk et al., 2012) affect connectivity estimation, our main priority was that the participants remained calm and awake during the recording, and the videos helped with this. On the other side, we would like to highlight how the absence of background noise and the limited physical constraints which characterise a fNIRS lab setting rather than a fMRI one could be ideal for resting‐state studies, where it is important to interfere as little as possible with participants’ mind‐wandering. In fact, because there is no requirement for participants to lay or sit perfectly still as in fMRI studies, fNIRS allows for the recording of resting state data in a more naturalistic setting. In this respect, researchers are currently working towards making this technology wireless and improving the flexibility and the comfort of the caps even further (Pinti et al., 2015; Zhao et al., 2019), aspects that future resting‐state studies can benefit from.

## 5 CONCLUSIONS

This is the first time that functional connectivity was estimated longitudinally in awake infants with fNIRS. Our results suggest a gradual increase of fronto‐temporoparietal connectivity over the first years of life, with a peak at 24 and 30 months which might indicate that by this age the DMN is fully developed. There seems to be a slight decrease after this point, which might be consistent with the process of connections pruning, starting at 2 years of age. From a methodological point of view, this study proposes a novel method of resting‐state data acquisition with awake infants, and provides a data analysis pipeline for the investigation of functional connectivity, which will facilitate the advancement of research in this field. We hope that fNIRS researchers interested in exploring functional connectivity in awake infants can benefit from this work.

## CONFLICT OF INTEREST

None declared.

## Supporting information


**Appendix S1** Supplementary Materials

## Data Availability

Research data are not shared.

## Endnotes

1 This study was part of a longitudinal project investigating the development of mimicry. Infants were also tested at 4 months but no resting state recordings had been taken at that visit.

2 At each visit, participants’ head measurements (circumference, the distance between ears over the forehead, distance between nasion to inion, distance between ears measured over the top of the head) were taken to select the correct cap size.

3 Based on the International 10–20 EEG placement system.

4 The TPJ was considered as the intersection of the STG, angular gyrus and supramarginal gyrus. Therefore, a channel is included in the TPJ ROI if it covers regions belonging to both the temporal and the parietal lobe. Only the more rostral channels labelled as angular gyrus were considered as part of the TPJ, while the others have been considered as posterior temporoparietal lobe.
