# Supplementary material for: The developmental trajectory of fronto‐temporoparietal connectivity as a proxy of the default mode network: a longitudinal fNIRS investigation
Source: Hum Brain Mapp. 2020 Mar 4;41(10):2717–40. doi: 10.1002/hbm.24974 (PMC7294062; doi:10.1002/hbm.24974)
Supplement: Supplementary file 1 — Appendix S1 Supplementary Materials [file HBM-41-2717-s001.docx]

**Supplementary Materials**

### Distribution of the signal duration included in the analysis

Supplementary Figure 1 shows the distribution of the of the bins length included in the analysis at each time point, which are positively skewed at every age (p<0.001).

**Supplementary Figure 1** Distribution of the bin lengths for each age group included in the analysis.

As we wanted to make sure that the number of short bins was not significantly higher in any of the age group, we have compared the percentage of the short bins (5-9.99 secs) with the percentage of the other bins between all the time points using chi-squares. Results show that there is no significant difference in the percentage of the short bins between ages. FDR corrected p-values are reported in the table below.

| ages | 11 months | 18 months | 24 months | 30 months | 36 months |
| --- | --- | --- | --- | --- | --- |
| 11 months |  | 0.314 | 0.075 | 0.075 | 0.678 |
| 18 months |  |  | 0.375 | 0.375 | 0.375 |
| 24 months |  |  |  | 0.81 | 0.132 |
| 30 months |  |  |  |  | 0.1 |
| 36 months |  |  |  |  |  |

**Supplementary Table 1.** FDR corrected p-values of the difference between the short bins and the other bins between all the time points.

### One sample t-test on reduced samples

Samples different per number of participants at every age. To assess whether difference patterns of connectivity are not driven by different in sample size, we randomly selected 15 participants and we tested again significant connectivity in the fronto-temporoparietal network and in the rest of the channels.

### Supplementary Figure 2 Graphical representations of the functional connections that are significantly different from zero, both in the fronto-temporoparietal regions and in the rest of the channels in N=15. HbO_2_ is plotted in red and HHb is plotted in blue. Connections that are significantly different from zero both in the HbO_2_ and the HHb signals are plotted in black.

### Similar patterns of connections that the ones observed in figure 7 are shown here, suggesting that differences in connectivity are not driven by difference in sample sizes.

### Differences in the fronto-temporoparietal connectivity between age groups

To test the hypothesis of an increased fronto-temporoparietal functional connectivity with age, the Fisher z-scores transformed correlation coefficients were compared with paired t-tests between the time points.

Supplementary Figure 1 shows graphical representations of the differences in the fronto-temporoparietal connections in the HbO_2_ and the HHb maps for comparison. Pairs of functional connections were included in the analysis only if at least half of the sample contributed data to the statistical tests. Supplementary Tables 1-10 show the degrees of freedom of the paired t-tests between the two age points of each connection.

In the HbO_2_ signal, there was a gradual increase of the fronto-temporoparietal connections until 24 months, while the number of connections that increase and decrease between 24 months and older ages is the same. In the HHb signal, the fronto-temporoparietal connections increased up to 30 months and, while there were no differences between 24 and 30 months, these connections increased again between 30 and 36 months. Comparisons between ages showed some similarities in numbers and locations of connections in the two signals. The only exception to this is the 11 months-36 months comparison, where the number of connections that increase is greater than those that decrease in the HbO_2_ signal, while the pattern is the opposite in the HHb signal.

In each comparison, connections that significantly increase or decrease in both the HbO_2_ and the HHb signal are very limited, therefore these results should be interpreted with cautions.

**Supplementary Figure 3.** Graphical representations of the differences in the fronto-temporoparietal in HbO_2_ and HHb between ages. Green lines show functional connections that increase with age and pink lines show functional connections that decrease with age. N indicates the number of included participants in each paired sample t-test.

Here we report the degrees of freedom of the t-tests between the two age points in each connection. Connections highlighted in grey are those excluded from the analysis as only half or less than half of the sample contributed to the statistical test.

|  | **1** | **2** | **3** | **4** | **5** | **6** | **7** | **8** | **9** | **10** | **11** | **12** | **13** | **14** | **15** | **16** | **17** | **18** | **19** | **20** | **21** | **22** | **23** | **24** | **25** | **26** | **27** | **28** | **29** | **30** |
| --- | --- | --- | --- | --- | --- | --- | --- | --- | --- | --- | --- | --- | --- | --- | --- | --- | --- | --- | --- | --- | --- | --- | --- | --- | --- | --- | --- | --- | --- | --- |
| **1** |  | 2 | 2 | 2 | 3 | 2 | 3 | 3 | 3 | 3 | 2 | 3 | 2 | 3 | 3 | 3 | 3 | 3 | 3 | 3 | 2 | 3 | 2 | 2 | 3 | 3 | 3 | 2 | 2 | 2 |
| **2** | 2 |  | 2 | 2 | 3 | 2 | 3 | 3 | 3 | 3 | 2 | 3 | 2 | 3 | 3 | 3 | 3 | 3 | 3 | 3 | 2 | 3 | 2 | 2 | 3 | 3 | 3 | 2 | 2 | 2 |
| **3** | 2 | 2 |  | 2 | 3 | 2 | 3 | 3 | 3 | 3 | 2 | 3 | 2 | 3 | 3 | 3 | 3 | 3 | 3 | 3 | 2 | 3 | 2 | 2 | 3 | 3 | 3 | 2 | 2 | 2 |
| **4** | 2 | 2 | 2 |  | 3 | 2 | 3 | 3 | 3 | 3 | 2 | 3 | 2 | 3 | 3 | 3 | 3 | 3 | 3 | 3 | 2 | 3 | 2 | 2 | 3 | 3 | 3 | 2 | 2 | 2 |
| **5** | 3 | 3 | 3 | 3 |  | 3 | 3 | 3 | 3 | 3 | 2 | 3 | 2 | 3 | 3 | 3 | 3 | 3 | 3 | 3 | 2 | 3 | 2 | 2 | 3 | 3 | 3 | 2 | 3 | 2 |
| **6** | 2 | 2 | 2 | 2 | 3 |  | 3 | 3 | 3 | 3 | 2 | 3 | 2 | 3 | 3 | 3 | 3 | 3 | 3 | 3 | 2 | 3 | 2 | 2 | 3 | 3 | 3 | 2 | 2 | 2 |
| **7** | 3 | 3 | 3 | 3 | 3 | 3 |  | 3 | 3 | 3 | 2 | 3 | 2 | 3 | 3 | 3 | 3 | 3 | 3 | 3 | 2 | 3 | 2 | 2 | 3 | 3 | 3 | 2 | 3 | 2 |
| **8** | 3 | 3 | 3 | 3 | 3 | 3 | 3 |  | 3 | 3 | 2 | 3 | 2 | 3 | 3 | 3 | 3 | 3 | 3 | 3 | 2 | 3 | 2 | 2 | 3 | 3 | 3 | 2 | 3 | 2 |
| **9** | 3 | 3 | 3 | 3 | 3 | 3 | 3 | 3 |  | 3 | 2 | 3 | 2 | 3 | 3 | 3 | 3 | 3 | 3 | 3 | 2 | 3 | 2 | 2 | 3 | 3 | 3 | 2 | 3 | 2 |
| **10** | 3 | 3 | 3 | 3 | 3 | 3 | 3 | 3 | 3 |  | 2 | 3 | 2 | 3 | 3 | 3 | 3 | 3 | 3 | 3 | 2 | 3 | 2 | 2 | 3 | 3 | 3 | 2 | 3 | 2 |
| **11** | 2 | 2 | 2 | 2 | 2 | 2 | 2 | 2 | 2 | 2 |  | 2 | 2 | 2 | 2 | 2 | 2 | 2 | 2 | 2 | 2 | 2 | 2 | 2 | 2 | 2 | 2 | 2 | 2 | 2 |
| **12** | 3 | 3 | 3 | 3 | 3 | 3 | 3 | 3 | 3 | 3 | 2 |  | 2 | 3 | 3 | 3 | 3 | 3 | 3 | 3 | 2 | 3 | 2 | 2 | 3 | 3 | 3 | 2 | 3 | 2 |
| **13** | 2 | 2 | 2 | 2 | 2 | 2 | 2 | 2 | 2 | 2 | 2 | 2 |  | 2 | 2 | 2 | 2 | 2 | 2 | 2 | 2 | 2 | 2 | 2 | 2 | 2 | 2 | 2 | 2 | 2 |
| **14** | 3 | 3 | 3 | 3 | 3 | 3 | 3 | 3 | 3 | 3 | 2 | 3 | 2 |  | 3 | 3 | 3 | 3 | 3 | 3 | 2 | 3 | 2 | 2 | 3 | 3 | 3 | 2 | 3 | 2 |
| **15** | 3 | 3 | 3 | 3 | 3 | 3 | 3 | 3 | 3 | 3 | 2 | 3 | 2 | 3 |  | 3 | 3 | 3 | 3 | 3 | 2 | 3 | 2 | 2 | 3 | 3 | 3 | 2 | 3 | 2 |
| **16** | 3 | 3 | 3 | 3 | 3 | 3 | 3 | 3 | 3 | 3 | 2 | 3 | 2 | 3 | 3 |  | 3 | 3 | 3 | 3 | 2 | 3 | 2 | 2 | 3 | 3 | 3 | 2 | 3 | 2 |
| **17** | 3 | 3 | 3 | 3 | 3 | 3 | 3 | 3 | 3 | 3 | 2 | 3 | 2 | 3 | 3 | 3 |  | 3 | 3 | 3 | 2 | 3 | 2 | 2 | 3 | 3 | 3 | 2 | 3 | 2 |
| **18** | 3 | 3 | 3 | 3 | 3 | 3 | 3 | 3 | 3 | 3 | 2 | 3 | 2 | 3 | 3 | 3 | 3 |  | 3 | 3 | 2 | 3 | 2 | 2 | 3 | 3 | 3 | 2 | 3 | 2 |
| **19** | 3 | 3 | 3 | 3 | 3 | 3 | 3 | 3 | 3 | 3 | 2 | 3 | 2 | 3 | 3 | 3 | 3 | 3 |  | 3 | 2 | 3 | 2 | 2 | 3 | 3 | 3 | 2 | 3 | 2 |
| **20** | 3 | 3 | 3 | 3 | 3 | 3 | 3 | 3 | 3 | 3 | 2 | 3 | 2 | 3 | 3 | 3 | 3 | 3 | 3 |  | 2 | 3 | 2 | 2 | 3 | 3 | 3 | 2 | 3 | 2 |
| **21** | 2 | 2 | 2 | 2 | 2 | 2 | 2 | 2 | 2 | 2 | 2 | 2 | 2 | 2 | 2 | 2 | 2 | 2 | 2 | 2 |  | 2 | 2 | 2 | 2 | 2 | 2 | 2 | 2 | 2 |
| **22** | 3 | 3 | 3 | 3 | 3 | 3 | 3 | 3 | 3 | 3 | 2 | 3 | 2 | 3 | 3 | 3 | 3 | 3 | 3 | 3 | 2 |  | 2 | 2 | 3 | 3 | 3 | 2 | 3 | 2 |
| **23** | 2 | 2 | 2 | 2 | 2 | 2 | 2 | 2 | 2 | 2 | 2 | 2 | 2 | 2 | 2 | 2 | 2 | 2 | 2 | 2 | 2 | 2 | 2 | 2 | 2 | 2 | 2 | 2 | 2 | 2 |
| **24** | 2 | 2 | 2 | 2 | 2 | 2 | 2 | 2 | 2 | 2 | 2 | 2 | 2 | 2 | 2 | 2 | 2 | 2 | 2 | 2 | 2 | 2 | 2 |  | 2 | 2 | 2 | 2 | 2 | 2 |
| **25** | 3 | 3 | 3 | 3 | 3 | 3 | 3 | 3 | 3 | 3 | 2 | 3 | 2 | 3 | 3 | 3 | 3 | 3 | 3 | 3 | 2 | 3 | 2 | 2 |  | 3 | 3 | 2 | 3 | 2 |
| **26** | 3 | 3 | 3 | 3 | 3 | 3 | 3 | 3 | 3 | 3 | 2 | 3 | 2 | 3 | 3 | 3 | 3 | 3 | 3 | 3 | 2 | 3 | 2 | 2 | 3 |  | 3 | 2 | 3 | 2 |
| **27** | 3 | 3 | 3 | 3 | 3 | 3 | 3 | 3 | 3 | 3 | 2 | 3 | 2 | 3 | 3 | 3 | 3 | 3 | 3 | 3 | 2 | 3 | 2 | 2 | 3 | 3 |  | 2 | 3 | 2 |
| **28** | 2 | 2 | 2 | 2 | 2 | 2 | 2 | 2 | 2 | 2 | 2 | 2 | 2 | 2 | 2 | 2 | 2 | 2 | 2 | 2 | 2 | 2 | 2 | 2 | 2 | 2 | 2 |  | 2 | 2 |
| **29** | 2 | 2 | 2 | 2 | 3 | 2 | 3 | 3 | 3 | 3 | 2 | 3 | 2 | 3 | 3 | 3 | 3 | 3 | 3 | 3 | 2 | 3 | 2 | 2 | 3 | 3 | 3 | 2 |  | 2 |
| **30** | 2 | 2 | 2 | 2 | 2 | 2 | 2 | 2 | 2 | 2 | 2 | 2 | 2 | 2 | 2 | 2 | 2 | 2 | 2 | 2 | 2 | 2 | 2 | 2 | 2 | 2 | 2 | 2 | 2 |  |

channels

channels

**Supplementary Table 2** Degrees of freedom of the paired t-tests between 11 months and 18 months in each connection.

|  | **1** | **2** | **3** | **4** | **5** | **6** | **7** | **8** | **9** | **10** | **11** | **12** | **13** | **14** | **15** | **16** | **17** | **18** | **19** | **20** | **21** | **22** | **23** | **24** | **25** | **26** | **27** | **28** | **29** | **30** |
| --- | --- | --- | --- | --- | --- | --- | --- | --- | --- | --- | --- | --- | --- | --- | --- | --- | --- | --- | --- | --- | --- | --- | --- | --- | --- | --- | --- | --- | --- | --- |
| **1** |  | 4 | 3 | 2 | 3 | 3 | 3 | 3 | 4 | 3 | 3 | 4 | 3 | 4 | 4 | 4 | 4 | 4 | 4 | 4 | 4 | 4 | 3 | 4 | 3 | 3 | 4 | 4 | 4 | 4 |
| **2** | 4 |  | 4 | 2 | 3 | 4 | 4 | 4 | 5 | 4 | 3 | 5 | 3 | 5 | 5 | 5 | 5 | 5 | 5 | 5 | 4 | 5 | 3 | 4 | 4 | 4 | 5 | 5 | 5 | 5 |
| **3** | 3 | 4 |  | 2 | 2 | 4 | 4 | 4 | 4 | 4 | 3 | 4 | 3 | 4 | 4 | 4 | 4 | 4 | 4 | 4 | 3 | 4 | 2 | 3 | 3 | 3 | 4 | 4 | 4 | 4 |
| **4** | 2 | 2 | 2 |  | 2 | 2 | 2 | 2 | 2 | 2 | 2 | 2 | 2 | 2 | 2 | 2 | 2 | 2 | 2 | 2 | 2 | 2 | 1 | 2 | 1 | 1 | 2 | 2 | 2 | 2 |
| **5** | 3 | 3 | 2 | 2 |  | 2 | 2 | 2 | 3 | 2 | 2 | 3 | 2 | 3 | 3 | 3 | 3 | 3 | 3 | 3 | 3 | 3 | 2 | 3 | 2 | 2 | 3 | 3 | 3 | 3 |
| **6** | 3 | 4 | 4 | 2 | 2 |  | 4 | 4 | 4 | 4 | 3 | 4 | 3 | 4 | 4 | 4 | 4 | 4 | 4 | 4 | 3 | 4 | 2 | 3 | 3 | 3 | 4 | 4 | 4 | 4 |
| **7** | 3 | 4 | 4 | 2 | 2 | 4 |  | 4 | 4 | 4 | 3 | 4 | 3 | 4 | 4 | 4 | 4 | 4 | 4 | 4 | 3 | 4 | 2 | 3 | 3 | 3 | 4 | 4 | 4 | 4 |
| **8** | 3 | 4 | 4 | 2 | 2 | 4 | 4 |  | 4 | 4 | 3 | 4 | 3 | 4 | 4 | 4 | 4 | 4 | 4 | 4 | 3 | 4 | 2 | 3 | 3 | 3 | 4 | 4 | 4 | 4 |
| **9** | 4 | 5 | 4 | 2 | 3 | 4 | 4 | 4 |  | 4 | 3 | 5 | 3 | 5 | 5 | 5 | 5 | 5 | 5 | 5 | 4 | 5 | 3 | 4 | 4 | 4 | 5 | 5 | 5 | 5 |
| **10** | 3 | 4 | 4 | 2 | 2 | 4 | 4 | 4 | 4 |  | 3 | 4 | 3 | 4 | 4 | 4 | 4 | 4 | 4 | 4 | 3 | 4 | 2 | 3 | 3 | 3 | 4 | 4 | 4 | 4 |
| **11** | 3 | 3 | 3 | 2 | 2 | 3 | 3 | 3 | 3 | 3 |  | 3 | 3 | 3 | 3 | 3 | 3 | 3 | 3 | 3 | 3 | 3 | 2 | 3 | 2 | 2 | 3 | 3 | 3 | 3 |
| **12** | 4 | 5 | 4 | 2 | 3 | 4 | 4 | 4 | 5 | 4 | 3 |  | 3 | 5 | 5 | 5 | 5 | 5 | 5 | 5 | 4 | 5 | 3 | 4 | 4 | 4 | 5 | 5 | 5 | 5 |
| **13** | 3 | 3 | 3 | 2 | 2 | 3 | 3 | 3 | 3 | 3 | 3 | 3 |  | 3 | 3 | 3 | 3 | 3 | 3 | 3 | 3 | 3 | 2 | 3 | 2 | 2 | 3 | 3 | 3 | 3 |
| **14** | 4 | 5 | 4 | 2 | 3 | 4 | 4 | 4 | 5 | 4 | 3 | 5 | 3 |  | 5 | 5 | 5 | 5 | 5 | 5 | 4 | 5 | 3 | 4 | 4 | 4 | 5 | 5 | 5 | 5 |
| **15** | 4 | 5 | 4 | 2 | 3 | 4 | 4 | 4 | 5 | 4 | 3 | 5 | 3 | 5 |  | 5 | 5 | 5 | 5 | 5 | 4 | 5 | 3 | 4 | 4 | 4 | 5 | 5 | 5 | 5 |
| **16** | 4 | 5 | 4 | 2 | 3 | 4 | 4 | 4 | 5 | 4 | 3 | 5 | 3 | 5 | 5 |  | 5 | 5 | 5 | 5 | 4 | 5 | 3 | 4 | 4 | 4 | 5 | 5 | 5 | 5 |
| **17** | 4 | 5 | 4 | 2 | 3 | 4 | 4 | 4 | 5 | 4 | 3 | 5 | 3 | 5 | 5 | 5 |  | 5 | 5 | 5 | 4 | 5 | 3 | 4 | 4 | 4 | 5 | 5 | 5 | 5 |
| **18** | 4 | 5 | 4 | 2 | 3 | 4 | 4 | 4 | 5 | 4 | 3 | 5 | 3 | 5 | 5 | 5 | 5 |  | 5 | 5 | 4 | 5 | 3 | 4 | 4 | 4 | 5 | 5 | 5 | 5 |
| **19** | 4 | 5 | 4 | 2 | 3 | 4 | 4 | 4 | 5 | 4 | 3 | 5 | 3 | 5 | 5 | 5 | 5 | 5 |  | 5 | 4 | 5 | 3 | 4 | 4 | 4 | 5 | 5 | 5 | 5 |
| **20** | 4 | 5 | 4 | 2 | 3 | 4 | 4 | 4 | 5 | 4 | 3 | 5 | 3 | 5 | 5 | 5 | 5 | 5 | 5 |  | 4 | 5 | 3 | 4 | 4 | 4 | 5 | 5 | 5 | 5 |
| **21** | 4 | 4 | 3 | 2 | 3 | 3 | 3 | 3 | 4 | 3 | 3 | 4 | 3 | 4 | 4 | 4 | 4 | 4 | 4 | 4 |  | 4 | 3 | 4 | 3 | 3 | 4 | 4 | 4 | 4 |
| **22** | 4 | 5 | 4 | 2 | 3 | 4 | 4 | 4 | 5 | 4 | 3 | 5 | 3 | 5 | 5 | 5 | 5 | 5 | 5 | 5 | 4 |  | 3 | 4 | 4 | 4 | 5 | 5 | 5 | 5 |
| **23** | 3 | 3 | 2 | 1 | 2 | 2 | 2 | 2 | 3 | 2 | 2 | 3 | 2 | 3 | 3 | 3 | 3 | 3 | 3 | 3 | 3 | 3 |  | 3 | 2 | 2 | 3 | 3 | 3 | 3 |
| **24** | 4 | 4 | 3 | 2 | 3 | 3 | 3 | 3 | 4 | 3 | 3 | 4 | 3 | 4 | 4 | 4 | 4 | 4 | 4 | 4 | 4 | 4 | 3 |  | 3 | 3 | 4 | 4 | 4 | 4 |
| **25** | 3 | 4 | 3 | 1 | 2 | 3 | 3 | 3 | 4 | 3 | 2 | 4 | 2 | 4 | 4 | 4 | 4 | 4 | 4 | 4 | 3 | 4 | 2 | 3 |  | 4 | 4 | 4 | 4 | 4 |
| **26** | 3 | 4 | 3 | 1 | 2 | 3 | 3 | 3 | 4 | 3 | 2 | 4 | 2 | 4 | 4 | 4 | 4 | 4 | 4 | 4 | 3 | 4 | 2 | 3 | 4 |  | 4 | 4 | 4 | 4 |
| **27** | 4 | 5 | 4 | 2 | 3 | 4 | 4 | 4 | 5 | 4 | 3 | 5 | 3 | 5 | 5 | 5 | 5 | 5 | 5 | 5 | 4 | 5 | 3 | 4 | 4 | 4 |  | 4 | 5 | 5 |
| **28** | 4 | 5 | 4 | 2 | 3 | 4 | 4 | 4 | 5 | 4 | 3 | 5 | 3 | 5 | 5 | 5 | 5 | 5 | 5 | 5 | 4 | 5 | 3 | 4 | 4 | 4 | 4 |  | 5 | 5 |
| **29** | 4 | 5 | 4 | 2 | 3 | 4 | 4 | 4 | 5 | 4 | 3 | 5 | 3 | 5 | 5 | 5 | 5 | 5 | 5 | 5 | 4 | 5 | 3 | 4 | 4 | 4 | 5 | 5 |  | 5 |
| **3** | 4 | 5 | 4 | 2 | 3 | 4 | 4 | 4 | 5 | 4 | 3 | 5 | 3 | 5 | 5 | 5 | 5 | 5 | 5 | 5 | 4 | 5 | 3 | 4 | 4 | 4 | 5 | 5 | 5 | 0 |

channels

channels

**Supplementary Table 3** Degrees of freedom of the paired t-tests between 11 months and 24 months in each connection.

|  | **1** | **2** | **3** | **4** | **5** | **6** | **7** | **8** | **9** | **10** | **11** | **12** | **13** | **14** | **15** | **16** | **17** | **18** | **19** | **20** | **21** | **22** | **23** | **24** | **25** | **26** | **27** | **28** | **29** | **30** |
| --- | --- | --- | --- | --- | --- | --- | --- | --- | --- | --- | --- | --- | --- | --- | --- | --- | --- | --- | --- | --- | --- | --- | --- | --- | --- | --- | --- | --- | --- | --- |
| **1** |  | 2 | 1 | 1 | 5 | 1 | 4 | 4 | 5 | 5 | 4 | 2 | 3 | 5 | 5 | 5 | 5 | 5 | 5 | 5 | 4 | 5 | 3 | 4 | 4 | 4 | 5 | 5 | 1 | 1 |
| **2** | 2 |  | 2 | 2 | 6 | 1 | 5 | 4 | 5 | 5 | 4 | 3 | 3 | 6 | 6 | 6 | 6 | 6 | 5 | 6 | 4 | 6 | 3 | 4 | 4 | 4 | 6 | 6 | 2 | 2 |
| **3** | 1 | 2 |  | 2 | 5 | 1 | 5 | 4 | 4 | 4 | 3 | 3 | 3 | 5 | 5 | 5 | 5 | 5 | 4 | 5 | 3 | 5 | 2 | 3 | 3 | 3 | 5 | 5 | 1 | 1 |
| **4** | 1 | 2 | 2 |  | 5 | 1 | 5 | 4 | 4 | 4 | 3 | 3 | 3 | 5 | 5 | 5 | 5 | 5 | 4 | 5 | 3 | 5 | 2 | 3 | 3 | 3 | 5 | 5 | 1 | 1 |
| **5** | 5 | 6 | 5 | 5 |  | 4 | 5 | 4 | 5 | 5 | 4 | 3 | 3 | 6 | 6 | 6 | 6 | 6 | 5 | 6 | 4 | 6 | 3 | 4 | 4 | 4 | 6 | 6 | 5 | 5 |
| **6** | 1 | 1 | 1 | 1 | 4 |  | 4 | 4 | 4 | 4 | 3 | 2 | 3 | 4 | 4 | 4 | 4 | 4 | 4 | 4 | 3 | 4 | 2 | 3 | 3 | 3 | 4 | 4 | 0 | 0 |
| **7** | 4 | 5 | 5 | 5 | 5 | 4 |  | 4 | 4 | 4 | 3 | 3 | 3 | 5 | 5 | 5 | 5 | 5 | 4 | 5 | 3 | 5 | 2 | 3 | 3 | 3 | 5 | 5 | 4 | 4 |
| **8** | 4 | 4 | 4 | 4 | 4 | 4 | 4 |  | 4 | 4 | 3 | 2 | 3 | 4 | 4 | 4 | 4 | 4 | 4 | 4 | 3 | 4 | 2 | 3 | 3 | 3 | 4 | 4 | 3 | 3 |
| **9** | 5 | 5 | 4 | 4 | 5 | 4 | 4 | 4 |  | 5 | 4 | 2 | 3 | 5 | 5 | 5 | 5 | 5 | 5 | 5 | 4 | 5 | 3 | 4 | 4 | 4 | 5 | 5 | 4 | 4 |
| **10** | 5 | 5 | 4 | 4 | 5 | 4 | 4 | 4 | 5 |  | 4 | 2 | 3 | 5 | 5 | 5 | 5 | 5 | 5 | 5 | 4 | 5 | 3 | 4 | 4 | 4 | 5 | 5 | 4 | 4 |
| **11** | 4 | 4 | 3 | 3 | 4 | 3 | 3 | 3 | 4 | 4 |  | 1 | 3 | 4 | 4 | 4 | 4 | 4 | 4 | 4 | 4 | 4 | 3 | 4 | 3 | 3 | 4 | 4 | 4 | 4 |
| **12** | 2 | 3 | 3 | 3 | 3 | 2 | 3 | 2 | 2 | 2 | 1 |  | 1 | 3 | 3 | 3 | 3 | 3 | 2 | 3 | 1 | 3 | 1 | 1 | 1 | 1 | 3 | 3 | 2 | 2 |
| **13** | 3 | 3 | 3 | 3 | 3 | 3 | 3 | 3 | 3 | 3 | 3 | 1 |  | 3 | 3 | 3 | 3 | 3 | 3 | 3 | 3 | 3 | 2 | 3 | 2 | 2 | 3 | 3 | 3 | 3 |
| **14** | 5 | 6 | 5 | 5 | 6 | 4 | 5 | 4 | 5 | 5 | 4 | 3 | 3 |  | 6 | 6 | 6 | 6 | 5 | 6 | 4 | 6 | 3 | 4 | 4 | 4 | 6 | 6 | 5 | 5 |
| **15** | 5 | 6 | 5 | 5 | 6 | 4 | 5 | 4 | 5 | 5 | 4 | 3 | 3 | 6 |  | 6 | 6 | 6 | 5 | 6 | 4 | 6 | 3 | 4 | 4 | 4 | 6 | 6 | 5 | 5 |
| **16** | 5 | 6 | 5 | 5 | 6 | 4 | 5 | 4 | 5 | 5 | 4 | 3 | 3 | 6 | 6 |  | 6 | 6 | 5 | 6 | 4 | 6 | 3 | 4 | 4 | 4 | 6 | 6 | 5 | 5 |
| **17** | 5 | 6 | 5 | 5 | 6 | 4 | 5 | 4 | 5 | 5 | 4 | 3 | 3 | 6 | 6 | 6 |  | 6 | 5 | 6 | 4 | 6 | 3 | 4 | 4 | 4 | 6 | 6 | 5 | 5 |
| **18** | 5 | 6 | 5 | 5 | 6 | 4 | 5 | 4 | 5 | 5 | 4 | 3 | 3 | 6 | 6 | 6 | 6 |  | 5 | 6 | 4 | 6 | 3 | 4 | 4 | 4 | 6 | 6 | 5 | 5 |
| **19** | 5 | 5 | 4 | 4 | 5 | 4 | 4 | 4 | 5 | 5 | 4 | 2 | 3 | 5 | 5 | 5 | 5 | 5 |  | 5 | 4 | 5 | 3 | 4 | 4 | 4 | 5 | 5 | 4 | 4 |
| **2** | 5 | 6 | 5 | 5 | 6 | 4 | 5 | 4 | 5 | 5 | 4 | 3 | 3 | 6 | 6 | 6 | 6 | 6 | 5 |  | 4 | 6 | 3 | 4 | 4 | 4 | 6 | 6 | 5 | 5 |
| **21** | 4 | 4 | 3 | 3 | 4 | 3 | 3 | 3 | 4 | 4 | 4 | 1 | 3 | 4 | 4 | 4 | 4 | 4 | 4 | 4 |  | 4 | 3 | 4 | 3 | 3 | 4 | 4 | 4 | 4 |
| **22** | 5 | 6 | 5 | 5 | 6 | 4 | 5 | 4 | 5 | 5 | 4 | 3 | 3 | 6 | 6 | 6 | 6 | 6 | 5 | 6 | 4 |  | 3 | 4 | 4 | 4 | 6 | 6 | 5 | 5 |
| **23** | 3 | 3 | 2 | 2 | 3 | 2 | 2 | 2 | 3 | 3 | 3 | 1 | 2 | 3 | 3 | 3 | 3 | 3 | 3 | 3 | 3 | 3 |  | 3 | 2 | 2 | 3 | 3 | 3 | 3 |
| **24** | 4 | 4 | 3 | 3 | 4 | 3 | 3 | 3 | 4 | 4 | 4 | 1 | 3 | 4 | 4 | 4 | 4 | 4 | 4 | 4 | 4 | 4 | 3 |  | 3 | 3 | 4 | 4 | 4 | 4 |
| **25** | 4 | 4 | 3 | 3 | 4 | 3 | 3 | 3 | 4 | 4 | 3 | 1 | 2 | 4 | 4 | 4 | 4 | 4 | 4 | 4 | 3 | 4 | 2 | 3 |  | 4 | 4 | 4 | 3 | 3 |
| **26** | 4 | 4 | 3 | 3 | 4 | 3 | 3 | 3 | 4 | 4 | 3 | 1 | 2 | 4 | 4 | 4 | 4 | 4 | 4 | 4 | 3 | 4 | 2 | 3 | 4 |  | 4 | 4 | 3 | 3 |
| **27** | 5 | 6 | 5 | 5 | 6 | 4 | 5 | 4 | 5 | 5 | 4 | 3 | 3 | 6 | 6 | 6 | 6 | 6 | 5 | 6 | 4 | 6 | 3 | 4 | 4 | 4 |  | 6 | 5 | 5 |
| **28** | 5 | 6 | 5 | 5 | 6 | 4 | 5 | 4 | 5 | 5 | 4 | 3 | 3 | 6 | 6 | 6 | 6 | 6 | 5 | 6 | 4 | 6 | 3 | 4 | 4 | 4 | 6 |  | 5 | 5 |
| **29** | 1 | 2 | 1 | 1 | 5 | 0 | 4 | 3 | 4 | 4 | 4 | 2 | 3 | 5 | 5 | 5 | 5 | 5 | 4 | 5 | 4 | 5 | 3 | 4 | 3 | 3 | 5 | 5 |  | 2 |
| **30** | 1 | 2 | 1 | 1 | 5 | 0 | 4 | 3 | 4 | 4 | 4 | 2 | 3 | 5 | 5 | 5 | 5 | 5 | 4 | 5 | 4 | 5 | 3 | 4 | 3 | 3 | 5 | 5 | 2 |  |

channels

channels

**Supplementary Table 4** Degrees of freedom of the paired t-tests between 11 months and 30 months in each connection.

|  | **1** | **2** | **3** | **4** | **5** | **6** | **7** | **8** | **9** | **10** | **11** | **12** | **13** | **14** | **15** | **16** | **17** | **18** | **19** | **20** | **21** | **22** | **23** | **24** | **25** | **26** | **27** | **28** | **29** | **30** |
| --- | --- | --- | --- | --- | --- | --- | --- | --- | --- | --- | --- | --- | --- | --- | --- | --- | --- | --- | --- | --- | --- | --- | --- | --- | --- | --- | --- | --- | --- | --- |
| **1** |  | 3 | 2 | 2 | 6 | 2 | 5 | 5 | 6 | 6 | 5 | 6 | 5 | 6 | 6 | 6 | 6 | 6 | 6 | 6 | 5 | 6 | 4 | 5 | 5 | 5 | 6 | 6 | 3 | 3 |
| **2** | 3 |  | 3 | 3 | 7 | 2 | 6 | 5 | 7 | 6 | 5 | 7 | 5 | 7 | 7 | 7 | 7 | 7 | 7 | 7 | 5 | 7 | 4 | 5 | 5 | 5 | 7 | 7 | 4 | 3 |
| **3** | 2 | 3 |  | 3 | 6 | 2 | 6 | 5 | 6 | 5 | 4 | 6 | 4 | 6 | 6 | 6 | 6 | 6 | 6 | 6 | 4 | 6 | 3 | 4 | 4 | 4 | 6 | 6 | 3 | 2 |
| **4** | 2 | 3 | 3 |  | 6 | 2 | 6 | 5 | 6 | 5 | 4 | 6 | 4 | 6 | 6 | 6 | 6 | 6 | 6 | 6 | 4 | 6 | 3 | 4 | 4 | 4 | 6 | 6 | 3 | 2 |
| **5** | 6 | 7 | 6 | 6 |  | 5 | 6 | 5 | 7 | 6 | 5 | 7 | 5 | 7 | 7 | 7 | 7 | 7 | 7 | 7 | 5 | 7 | 4 | 5 | 5 | 5 | 7 | 7 | 7 | 6 |
| **6** | 2 | 2 | 2 | 2 | 5 |  | 5 | 5 | 5 | 5 | 4 | 5 | 4 | 5 | 5 | 5 | 5 | 5 | 5 | 5 | 4 | 5 | 3 | 4 | 4 | 4 | 5 | 5 | 2 | 2 |
| **7** | 5 | 6 | 6 | 6 | 6 | 5 |  | 5 | 6 | 5 | 4 | 6 | 4 | 6 | 6 | 6 | 6 | 6 | 6 | 6 | 4 | 6 | 3 | 4 | 4 | 4 | 6 | 6 | 6 | 5 |
| **8** | 5 | 5 | 5 | 5 | 5 | 5 | 5 |  | 5 | 5 | 4 | 5 | 4 | 5 | 5 | 5 | 5 | 5 | 5 | 5 | 4 | 5 | 3 | 4 | 4 | 4 | 5 | 5 | 5 | 5 |
| **9** | 6 | 7 | 6 | 6 | 7 | 5 | 6 | 5 |  | 6 | 5 | 7 | 5 | 7 | 7 | 7 | 7 | 7 | 7 | 7 | 5 | 7 | 4 | 5 | 5 | 5 | 7 | 7 | 7 | 6 |
| **10** | 6 | 6 | 5 | 5 | 6 | 5 | 5 | 5 | 6 |  | 5 | 6 | 5 | 6 | 6 | 6 | 6 | 6 | 6 | 6 | 5 | 6 | 4 | 5 | 5 | 5 | 6 | 6 | 6 | 6 |
| **11** | 5 | 5 | 4 | 4 | 5 | 4 | 4 | 4 | 5 | 5 |  | 5 | 5 | 5 | 5 | 5 | 5 | 5 | 5 | 5 | 5 | 5 | 4 | 5 | 4 | 4 | 5 | 5 | 5 | 5 |
| **12** | 6 | 7 | 6 | 6 | 7 | 5 | 6 | 5 | 7 | 6 | 5 |  | 5 | 7 | 7 | 7 | 7 | 7 | 7 | 7 | 5 | 7 | 4 | 5 | 5 | 5 | 7 | 7 | 7 | 6 |
| **13** | 5 | 5 | 4 | 4 | 5 | 4 | 4 | 4 | 5 | 5 | 5 | 5 |  | 5 | 5 | 5 | 5 | 5 | 5 | 5 | 5 | 5 | 4 | 5 | 4 | 4 | 5 | 5 | 5 | 5 |
| **14** | 6 | 7 | 6 | 6 | 7 | 5 | 6 | 5 | 7 | 6 | 5 | 7 | 5 |  | 7 | 7 | 7 | 7 | 7 | 7 | 5 | 7 | 4 | 5 | 5 | 5 | 7 | 7 | 7 | 6 |
| **15** | 6 | 7 | 6 | 6 | 7 | 5 | 6 | 5 | 7 | 6 | 5 | 7 | 5 | 7 |  | 7 | 7 | 7 | 7 | 7 | 5 | 7 | 4 | 5 | 5 | 5 | 7 | 7 | 7 | 6 |
| **16** | 6 | 7 | 6 | 6 | 7 | 5 | 6 | 5 | 7 | 6 | 5 | 7 | 5 | 7 | 7 |  | 7 | 7 | 7 | 7 | 5 | 7 | 4 | 5 | 5 | 5 | 7 | 7 | 7 | 6 |
| **17** | 6 | 7 | 6 | 6 | 7 | 5 | 6 | 5 | 7 | 6 | 5 | 7 | 5 | 7 | 7 | 7 |  | 7 | 7 | 7 | 5 | 7 | 4 | 5 | 5 | 5 | 7 | 7 | 7 | 6 |
| **18** | 6 | 7 | 6 | 6 | 7 | 5 | 6 | 5 | 7 | 6 | 5 | 7 | 5 | 7 | 7 | 7 | 7 |  | 7 | 7 | 5 | 7 | 4 | 5 | 5 | 5 | 7 | 7 | 7 | 6 |
| **19** | 6 | 7 | 6 | 6 | 7 | 5 | 6 | 5 | 7 | 6 | 5 | 7 | 5 | 7 | 7 | 7 | 7 | 7 |  | 7 | 5 | 7 | 4 | 5 | 5 | 5 | 7 | 7 | 7 | 6 |
| **20** | 6 | 7 | 6 | 6 | 7 | 5 | 6 | 5 | 7 | 6 | 5 | 7 | 5 | 7 | 7 | 7 | 7 | 7 | 7 |  | 5 | 7 | 4 | 5 | 5 | 5 | 7 | 7 | 7 | 6 |
| **21** | 5 | 5 | 4 | 4 | 5 | 4 | 4 | 4 | 5 | 5 | 5 | 5 | 5 | 5 | 5 | 5 | 5 | 5 | 5 | 5 |  | 5 | 4 | 5 | 4 | 4 | 5 | 5 | 5 | 5 |
| **22** | 6 | 7 | 6 | 6 | 7 | 5 | 6 | 5 | 7 | 6 | 5 | 7 | 5 | 7 | 7 | 7 | 7 | 7 | 7 | 7 | 5 |  | 4 | 5 | 5 | 5 | 7 | 7 | 7 | 6 |
| **23** | 4 | 4 | 3 | 3 | 4 | 3 | 3 | 3 | 4 | 4 | 4 | 4 | 4 | 4 | 4 | 4 | 4 | 4 | 4 | 4 | 4 | 4 |  | 4 | 3 | 3 | 4 | 4 | 4 | 4 |
| **24** | 5 | 5 | 4 | 4 | 5 | 4 | 4 | 4 | 5 | 5 | 5 | 5 | 5 | 5 | 5 | 5 | 5 | 5 | 5 | 5 | 5 | 5 | 4 |  | 4 | 4 | 5 | 5 | 5 | 5 |
| **25** | 5 | 5 | 4 | 4 | 5 | 4 | 4 | 4 | 5 | 5 | 4 | 5 | 4 | 5 | 5 | 5 | 5 | 5 | 5 | 5 | 4 | 5 | 3 | 4 |  | 5 | 5 | 5 | 5 | 5 |
| **26** | 5 | 5 | 4 | 4 | 5 | 4 | 4 | 4 | 5 | 5 | 4 | 5 | 4 | 5 | 5 | 5 | 5 | 5 | 5 | 5 | 4 | 5 | 3 | 4 | 5 |  | 5 | 5 | 5 | 5 |
| **27** | 6 | 7 | 6 | 6 | 7 | 5 | 6 | 5 | 7 | 6 | 5 | 7 | 5 | 7 | 7 | 7 | 7 | 7 | 7 | 7 | 5 | 7 | 4 | 5 | 5 | 5 |  | 7 | 7 | 6 |
| **28** | 6 | 7 | 6 | 6 | 7 | 5 | 6 | 5 | 7 | 6 | 5 | 7 | 5 | 7 | 7 | 7 | 7 | 7 | 7 | 7 | 5 | 7 | 4 | 5 | 5 | 5 | 7 |  | 7 | 6 |
| **29** | 3 | 4 | 3 | 3 | 7 | 2 | 6 | 5 | 7 | 6 | 5 | 7 | 5 | 7 | 7 | 7 | 7 | 7 | 7 | 7 | 5 | 7 | 4 | 5 | 5 | 5 | 7 | 7 |  | 3 |
| **30** | 3 | 3 | 2 | 2 | 6 | 2 | 5 | 5 | 6 | 6 | 5 | 6 | 5 | 6 | 6 | 6 | 6 | 6 | 6 | 6 | 5 | 6 | 4 | 5 | 5 | 5 | 6 | 6 | 3 |  |

channels

channels

**Supplementary Table 5** Degrees of freedom of the paired t-tests between 11 months and 36 months in each connection.

channels

channels

**Supplementary Table 6** Degrees of freedom of the paired t-tests between 18 months and 24 months in each connection.

|  | **1** | **2** | **3** | **4** | **5** | **6** | **7** | **8** | **9** | **10** | **11** | **12** | **13** | **14** | **15** | **16** | **17** | **18** | **19** | **20** | **21** | **22** | **23** | **24** | **25** | **26** | **27** | **28** | **29** | **30** |
| --- | --- | --- | --- | --- | --- | --- | --- | --- | --- | --- | --- | --- | --- | --- | --- | --- | --- | --- | --- | --- | --- | --- | --- | --- | --- | --- | --- | --- | --- | --- |
| **1** |  | 6 | 6 | 5 | 5 | 6 | 6 | 6 | 6 | 6 | 6 | 5 | 5 | 6 | 6 | 6 | 6 | 6 | 6 | 6 | 6 | 6 | 6 | 5 | 6 | 5 | 5 | 6 | 6 | 8 |
| **2** | 6 |  | 10 | 5 | 5 | 10 | 9 | 10 | 10 | 10 | 9 | 9 | 9 | 10 | 10 | 10 | 10 | 10 | 10 | 10 | 10 | 10 | 10 | 9 | 10 | 9 | 9 | 9 | 10 | 7 |
| **3** | 6 | 10 |  | 5 | 5 | 10 | 9 | 10 | 10 | 10 | 9 | 9 | 9 | 10 | 10 | 10 | 10 | 10 | 10 | 10 | 10 | 10 | 10 | 9 | 10 | 9 | 9 | 9 | 10 | 7 |
| **4** | 5 | 5 | 5 |  | 5 | 5 | 5 | 5 | 5 | 5 | 5 | 8 | 8 | 5 | 5 | 5 | 5 | 5 | 5 | 5 | 5 | 5 | 5 | 8 | 5 | 8 | 8 | 5 | 5 | 7 |
| **5** | 5 | 5 | 5 | 5 |  | 5 | 5 | 5 | 5 | 5 | 5 | 8 | 8 | 5 | 5 | 5 | 5 | 5 | 5 | 5 | 5 | 5 | 5 | 8 | 5 | 8 | 8 | 5 | 5 | 7 |
| **6** | 6 | 10 | 10 | 5 | 5 |  | 9 | 10 | 10 | 10 | 9 | 9 | 9 | 10 | 10 | 10 | 10 | 10 | 10 | 10 | 10 | 10 | 10 | 9 | 10 | 9 | 9 | 9 | 10 | 7 |
| **7** | 6 | 9 | 9 | 5 | 5 | 9 |  | 9 | 9 | 9 | 9 | 8 | 8 | 9 | 9 | 9 | 9 | 9 | 9 | 9 | 9 | 9 | 9 | 8 | 9 | 8 | 8 | 8 | 9 | 6 |
| **8** | 6 | 10 | 10 | 5 | 5 | 10 | 9 |  | 10 | 10 | 9 | 9 | 9 | 10 | 10 | 10 | 10 | 10 | 10 | 10 | 10 | 10 | 10 | 9 | 10 | 9 | 9 | 9 | 10 | 7 |
| **9** | 6 | 10 | 10 | 5 | 5 | 10 | 9 | 10 |  | 10 | 9 | 9 | 9 | 10 | 10 | 10 | 10 | 10 | 10 | 10 | 10 | 10 | 10 | 9 | 10 | 9 | 9 | 9 | 10 | 7 |
| **10** | 6 | 10 | 10 | 5 | 5 | 10 | 9 | 10 | 10 |  | 9 | 9 | 9 | 10 | 10 | 10 | 10 | 10 | 10 | 10 | 10 | 10 | 10 | 9 | 10 | 9 | 9 | 9 | 10 | 7 |
| **11** | 6 | 9 | 9 | 5 | 5 | 9 | 9 | 9 | 9 | 9 |  | 8 | 8 | 9 | 9 | 9 | 9 | 9 | 9 | 9 | 9 | 9 | 9 | 8 | 9 | 8 | 8 | 8 | 9 | 6 |
| **12** | 5 | 9 | 9 | 8 | 8 | 9 | 8 | 9 | 9 | 9 | 8 |  | 9 | 9 | 9 | 9 | 9 | 9 | 9 | 9 | 9 | 9 | 9 | 8 | 9 | 8 | 8 | 8 | 9 | 6 |
| **13** | 5 | 9 | 9 | 8 | 8 | 9 | 8 | 9 | 9 | 9 | 8 | 9 |  | 9 | 9 | 9 | 9 | 9 | 9 | 9 | 9 | 9 | 9 | 8 | 9 | 8 | 8 | 8 | 9 | 6 |
| **14** | 6 | 10 | 10 | 5 | 5 | 10 | 9 | 10 | 10 | 10 | 9 | 9 | 9 |  | 10 | 10 | 10 | 10 | 10 | 10 | 10 | 10 | 10 | 9 | 10 | 9 | 9 | 9 | 10 | 7 |
| **15** | 6 | 10 | 10 | 5 | 5 | 10 | 9 | 10 | 10 | 10 | 9 | 9 | 9 | 10 |  | 10 | 10 | 10 | 10 | 10 | 10 | 10 | 10 | 9 | 10 | 9 | 9 | 9 | 10 | 7 |
| **16** | 6 | 10 | 10 | 5 | 5 | 10 | 9 | 10 | 10 | 10 | 9 | 9 | 9 | 10 | 10 |  | 10 | 10 | 10 | 10 | 10 | 10 | 10 | 9 | 10 | 9 | 9 | 9 | 10 | 7 |
| **17** | 6 | 10 | 10 | 5 | 5 | 10 | 9 | 10 | 10 | 10 | 9 | 9 | 9 | 10 | 10 | 10 |  | 10 | 10 | 10 | 10 | 10 | 10 | 9 | 10 | 9 | 9 | 9 | 10 | 7 |
| **18** | 6 | 10 | 10 | 5 | 5 | 10 | 9 | 10 | 10 | 10 | 9 | 9 | 9 | 10 | 10 | 10 | 10 |  | 10 | 10 | 10 | 10 | 10 | 9 | 10 | 9 | 9 | 9 | 10 | 7 |
| **19** | 6 | 10 | 10 | 5 | 5 | 10 | 9 | 10 | 10 | 10 | 9 | 9 | 9 | 10 | 10 | 10 | 10 | 10 |  | 10 | 10 | 10 | 10 | 9 | 10 | 9 | 9 | 9 | 10 | 7 |
| **20** | 6 | 10 | 10 | 5 | 5 | 10 | 9 | 10 | 10 | 10 | 9 | 9 | 9 | 10 | 10 | 10 | 10 | 10 | 10 |  | 10 | 10 | 10 | 9 | 10 | 9 | 9 | 9 | 10 | 7 |
| **21** | 6 | 10 | 10 | 5 | 5 | 10 | 9 | 10 | 10 | 10 | 9 | 9 | 9 | 10 | 10 | 10 | 10 | 10 | 10 | 10 |  | 10 | 10 | 9 | 10 | 9 | 9 | 9 | 10 | 7 |
| **22** | 6 | 10 | 10 | 5 | 5 | 10 | 9 | 10 | 10 | 10 | 9 | 9 | 9 | 10 | 10 | 10 | 10 | 10 | 10 | 10 | 10 |  | 10 | 9 | 10 | 9 | 9 | 9 | 10 | 7 |
| **23** | 6 | 10 | 10 | 5 | 5 | 10 | 9 | 10 | 10 | 10 | 9 | 9 | 9 | 10 | 10 | 10 | 10 | 10 | 10 | 10 | 10 | 10 |  | 9 | 10 | 9 | 9 | 9 | 10 | 7 |
| **24** | 5 | 9 | 9 | 8 | 8 | 9 | 8 | 9 | 9 | 9 | 8 | 8 | 8 | 9 | 9 | 9 | 9 | 9 | 9 | 9 | 9 | 9 | 9 |  | 9 | 9 | 9 | 8 | 9 | 7 |
| **25** | 6 | 10 | 10 | 5 | 5 | 10 | 9 | 10 | 10 | 10 | 9 | 9 | 9 | 10 | 10 | 10 | 10 | 10 | 10 | 10 | 10 | 10 | 10 | 9 |  | 9 | 9 | 9 | 10 | 7 |
| **26** | 5 | 9 | 9 | 8 | 8 | 9 | 8 | 9 | 9 | 9 | 8 | 8 | 8 | 9 | 9 | 9 | 9 | 9 | 9 | 9 | 9 | 9 | 9 | 9 | 9 |  | 9 | 8 | 9 | 7 |
| **27** | 5 | 9 | 9 | 8 | 8 | 9 | 8 | 9 | 9 | 9 | 8 | 8 | 8 | 9 | 9 | 9 | 9 | 9 | 9 | 9 | 9 | 9 | 9 | 9 | 9 | 9 |  | 6 | 9 | 7 |
| **28** | 6 | 9 | 9 | 5 | 5 | 9 | 8 | 9 | 9 | 9 | 8 | 8 | 8 | 9 | 9 | 9 | 9 | 9 | 9 | 9 | 9 | 9 | 9 | 8 | 9 | 8 | 6 |  | 9 | 7 |
| **29** | 6 | 10 | 10 | 5 | 5 | 10 | 9 | 10 | 10 | 10 | 9 | 9 | 9 | 10 | 10 | 10 | 10 | 10 | 10 | 10 | 10 | 10 | 10 | 9 | 10 | 9 | 9 | 9 |  | 7 |
| **30** | 8 | 7 | 7 | 7 | 3 | 7 | 6 | 7 | 7 | 7 | 6 | 6 | 6 | 7 | 7 | 7 | 7 | 7 | 7 | 7 | 7 | 7 | 7 | 7 | 7 | 7 | 7 | 7 | 7 |  |

|  | **1** | **2** | **3** | **4** | **5** | **6** | **7** | **8** | **9** | **10** | **11** | **12** | **13** | **14** | **15** | **16** | **17** | **18** | **19** | **20** | **21** | **22** | **23** | **24** | **25** | **26** | **27** | **28** | **29** | **30** |
| --- | --- | --- | --- | --- | --- | --- | --- | --- | --- | --- | --- | --- | --- | --- | --- | --- | --- | --- | --- | --- | --- | --- | --- | --- | --- | --- | --- | --- | --- | --- |
| **1** |  | 9 | 9 | 9 | 9 | 9 | 9 | 9 | 9 | 9 | 9 | 6 | 7 | 9 | 9 | 9 | 9 | 9 | 9 | 9 | 9 | 9 | 9 | 8 | 9 | 8 | 9 | 8 | 8 | 7 |
| **2** | 9 |  | 9 | 9 | 9 | 9 | 9 | 9 | 9 | 9 | 9 | 6 | 7 | 9 | 9 | 9 | 9 | 9 | 9 | 9 | 9 | 9 | 9 | 8 | 9 | 8 | 9 | 8 | 8 | 7 |
| **3** | 9 | 9 |  | 9 | 9 | 9 | 9 | 9 | 9 | 9 | 9 | 6 | 7 | 9 | 9 | 9 | 9 | 9 | 9 | 9 | 9 | 9 | 9 | 8 | 9 | 8 | 9 | 8 | 8 | 7 |
| **4** | 9 | 9 | 9 |  | 9 | 9 | 9 | 9 | 9 | 9 | 9 | 6 | 7 | 9 | 9 | 9 | 9 | 9 | 9 | 9 | 9 | 9 | 9 | 8 | 9 | 8 | 9 | 8 | 8 | 7 |
| **5** | 9 | 9 | 9 | 9 |  | 9 | 9 | 9 | 9 | 9 | 9 | 6 | 7 | 9 | 9 | 9 | 9 | 9 | 9 | 9 | 9 | 9 | 9 | 8 | 9 | 8 | 9 | 8 | 8 | 7 |
| **6** | 9 | 9 | 9 | 9 | 9 |  | 9 | 9 | 9 | 9 | 9 | 6 | 7 | 9 | 9 | 9 | 9 | 9 | 9 | 9 | 9 | 9 | 9 | 8 | 9 | 8 | 9 | 8 | 8 | 7 |
| **7** | 9 | 9 | 9 | 9 | 9 | 9 |  | 9 | 9 | 9 | 9 | 6 | 7 | 9 | 9 | 9 | 9 | 9 | 9 | 9 | 9 | 9 | 9 | 8 | 9 | 8 | 9 | 8 | 8 | 7 |
| **8** | 9 | 9 | 9 | 9 | 9 | 9 | 9 |  | 9 | 9 | 9 | 6 | 7 | 9 | 9 | 9 | 9 | 9 | 9 | 9 | 9 | 9 | 9 | 8 | 9 | 8 | 9 | 8 | 8 | 7 |
| **9** | 9 | 9 | 9 | 9 | 9 | 9 | 9 | 9 |  | 9 | 9 | 6 | 7 | 9 | 9 | 9 | 9 | 9 | 9 | 9 | 9 | 9 | 9 | 8 | 9 | 8 | 9 | 8 | 8 | 7 |
| **10** | 9 | 9 | 9 | 9 | 9 | 9 | 9 | 9 | 9 |  | 9 | 6 | 7 | 9 | 9 | 9 | 9 | 9 | 9 | 9 | 9 | 9 | 9 | 8 | 9 | 8 | 9 | 8 | 8 | 7 |
| **11** | 9 | 9 | 9 | 9 | 9 | 9 | 9 | 9 | 9 | 9 |  | 6 | 7 | 9 | 9 | 9 | 9 | 9 | 9 | 9 | 9 | 9 | 9 | 8 | 9 | 8 | 9 | 8 | 8 | 7 |
| **12** | 6 | 6 | 6 | 6 | 6 | 6 | 6 | 6 | 6 | 6 | 6 |  | 5 | 6 | 6 | 6 | 6 | 6 | 6 | 6 | 6 | 6 | 6 | 5 | 6 | 5 | 6 | 5 | 5 | 5 |
| **13** | 7 | 7 | 7 | 7 | 7 | 7 | 7 | 7 | 7 | 7 | 7 | 5 |  | 7 | 7 | 7 | 7 | 7 | 7 | 7 | 7 | 7 | 7 | 6 | 7 | 6 | 7 | 6 | 6 | 5 |
| **14** | 9 | 9 | 9 | 9 | 9 | 9 | 9 | 9 | 9 | 9 | 9 | 6 | 7 |  | 9 | 9 | 9 | 9 | 9 | 9 | 9 | 9 | 9 | 8 | 9 | 8 | 9 | 8 | 8 | 7 |
| **15** | 9 | 9 | 9 | 9 | 9 | 9 | 9 | 9 | 9 | 9 | 9 | 6 | 7 | 9 |  | 9 | 9 | 9 | 9 | 9 | 9 | 9 | 9 | 8 | 9 | 8 | 9 | 8 | 8 | 7 |
| **16** | 9 | 9 | 9 | 9 | 9 | 9 | 9 | 9 | 9 | 9 | 9 | 6 | 7 | 9 | 9 |  | 9 | 9 | 9 | 9 | 9 | 9 | 9 | 8 | 9 | 8 | 9 | 8 | 8 | 7 |
| **17** | 9 | 9 | 9 | 9 | 9 | 9 | 9 | 9 | 9 | 9 | 9 | 6 | 7 | 9 | 9 | 9 |  | 9 | 9 | 9 | 9 | 9 | 9 | 8 | 9 | 8 | 9 | 8 | 8 | 7 |
| **18** | 9 | 9 | 9 | 9 | 9 | 9 | 9 | 9 | 9 | 9 | 9 | 6 | 7 | 9 | 9 | 9 | 9 |  | 9 | 9 | 9 | 9 | 9 | 8 | 9 | 8 | 9 | 8 | 8 | 7 |
| **19** | 9 | 9 | 9 | 9 | 9 | 9 | 9 | 9 | 9 | 9 | 9 | 6 | 7 | 9 | 9 | 9 | 9 | 9 |  | 9 | 9 | 9 | 9 | 8 | 9 | 8 | 9 | 8 | 8 | 7 |
| **20** | 9 | 9 | 9 | 9 | 9 | 9 | 9 | 9 | 9 | 9 | 9 | 6 | 7 | 9 | 9 | 9 | 9 | 9 | 9 |  | 9 | 9 | 9 | 8 | 9 | 8 | 9 | 8 | 8 | 7 |
| **21** | 9 | 9 | 9 | 9 | 9 | 9 | 9 | 9 | 9 | 9 | 9 | 6 | 7 | 9 | 9 | 9 | 9 | 9 | 9 | 9 |  | 9 | 9 | 8 | 9 | 8 | 9 | 8 | 8 | 7 |
| **22** | 9 | 9 | 9 | 9 | 9 | 9 | 9 | 9 | 9 | 9 | 9 | 6 | 7 | 9 | 9 | 9 | 9 | 9 | 9 | 9 | 9 |  | 9 | 8 | 9 | 8 | 9 | 8 | 8 | 7 |
| **23** | 9 | 9 | 9 | 9 | 9 | 9 | 9 | 9 | 9 | 9 | 9 | 6 | 7 | 9 | 9 | 9 | 9 | 9 | 9 | 9 | 9 | 9 |  | 8 | 9 | 8 | 9 | 8 | 8 | 7 |
| **24** | 8 | 8 | 8 | 8 | 8 | 8 | 8 | 8 | 8 | 8 | 8 | 5 | 6 | 8 | 8 | 8 | 8 | 8 | 8 | 8 | 8 | 8 | 8 |  | 8 | 8 | 8 | 7 | 7 | 6 |
| **25** | 9 | 9 | 9 | 9 | 9 | 9 | 9 | 9 | 9 | 9 | 9 | 6 | 7 | 9 | 9 | 9 | 9 | 9 | 9 | 9 | 9 | 9 | 9 | 8 |  | 8 | 9 | 8 | 8 | 7 |
| **26** | 8 | 8 | 8 | 8 | 8 | 8 | 8 | 8 | 8 | 8 | 8 | 5 | 6 | 8 | 8 | 8 | 8 | 8 | 8 | 8 | 8 | 8 | 8 | 8 | 8 |  | 8 | 7 | 7 | 6 |
| **27** | 9 | 9 | 9 | 9 | 9 | 9 | 9 | 9 | 9 | 9 | 9 | 6 | 7 | 9 | 9 | 9 | 9 | 9 | 9 | 9 | 9 | 9 | 9 | 8 | 9 | 8 |  | 8 | 8 | 7 |
| **28** | 8 | 8 | 8 | 8 | 8 | 8 | 8 | 8 | 8 | 8 | 8 | 5 | 6 | 8 | 8 | 8 | 8 | 8 | 8 | 8 | 8 | 8 | 8 | 7 | 8 | 7 | 8 |  | 8 | 7 |
| **29** | 8 | 8 | 8 | 8 | 8 | 8 | 8 | 8 | 8 | 8 | 8 | 5 | 6 | 8 | 8 | 8 | 8 | 8 | 8 | 8 | 8 | 8 | 8 | 7 | 8 | 7 | 8 | 8 |  | 7 |
| **30** | 7 | 7 | 7 | 7 | 7 | 7 | 7 | 7 | 7 | 7 | 7 | 5 | 5 | 7 | 7 | 7 | 7 | 7 | 7 | 7 | 7 | 7 | 7 | 6 | 7 | 6 | 7 | 7 | 7 |  |

channels

channels

**Supplementary Table 7** Degrees of freedom of the paired t-tests between 18 months and 30 months in each connection.

|  | **1** | **2** | **3** | **4** | **5** | **6** | **7** | **8** | **9** | **10** | **11** | **12** | **13** | **14** | **15** | **16** | **17** | **18** | **19** | **20** | **21** | **22** | **23** | **24** | **25** | **26** | **27** | **28** | **29** | **30** |
| --- | --- | --- | --- | --- | --- | --- | --- | --- | --- | --- | --- | --- | --- | --- | --- | --- | --- | --- | --- | --- | --- | --- | --- | --- | --- | --- | --- | --- | --- | --- |
| **1** |  | 14 | 14 | 14 | 14 | 14 | 13 | 13 | 14 | 12 | 14 | 13 | 13 | 14 | 14 | 14 | 14 | 14 | 13 | 13 | 13 | 12 | 13 | 12 | 13 | 11 | 13 | 13 | 14 | 12 |
| **2** | 14 |  | 14 | 14 | 14 | 14 | 13 | 13 | 14 | 12 | 14 | 13 | 13 | 14 | 14 | 14 | 14 | 14 | 13 | 13 | 13 | 12 | 13 | 12 | 13 | 11 | 13 | 13 | 14 | 12 |
| **3** | 14 | 14 |  | 14 | 14 | 14 | 13 | 13 | 14 | 12 | 14 | 13 | 13 | 14 | 14 | 14 | 14 | 14 | 13 | 13 | 13 | 12 | 13 | 12 | 13 | 11 | 13 | 13 | 14 | 12 |
| **4** | 14 | 14 | 14 |  | 14 | 14 | 13 | 13 | 14 | 12 | 14 | 13 | 13 | 14 | 14 | 14 | 14 | 14 | 13 | 13 | 13 | 12 | 13 | 12 | 13 | 11 | 13 | 13 | 14 | 12 |
| **5** | 14 | 14 | 14 | 14 |  | 14 | 13 | 13 | 14 | 12 | 14 | 13 | 13 | 14 | 14 | 14 | 14 | 14 | 13 | 13 | 13 | 12 | 13 | 12 | 13 | 11 | 13 | 13 | 14 | 12 |
| **6** | 14 | 14 | 14 | 14 | 14 |  | 13 | 13 | 14 | 12 | 14 | 13 | 13 | 14 | 14 | 14 | 14 | 14 | 13 | 13 | 13 | 12 | 13 | 12 | 13 | 11 | 13 | 13 | 14 | 12 |
| **7** | 13 | 13 | 13 | 13 | 13 | 13 |  | 13 | 13 | 12 | 13 | 13 | 13 | 13 | 13 | 13 | 13 | 13 | 13 | 13 | 13 | 12 | 13 | 12 | 13 | 11 | 12 | 12 | 13 | 11 |
| **8** | 13 | 13 | 13 | 13 | 13 | 13 | 13 |  | 13 | 12 | 13 | 13 | 13 | 13 | 13 | 13 | 13 | 13 | 13 | 13 | 13 | 12 | 13 | 12 | 13 | 11 | 12 | 12 | 13 | 11 |
| **9** | 14 | 14 | 14 | 14 | 14 | 14 | 13 | 13 |  | 12 | 14 | 13 | 13 | 14 | 14 | 14 | 14 | 14 | 13 | 13 | 13 | 12 | 13 | 12 | 13 | 11 | 13 | 13 | 14 | 12 |
| **10** | 12 | 12 | 12 | 12 | 12 | 12 | 12 | 12 | 12 |  | 12 | 12 | 12 | 12 | 12 | 12 | 12 | 12 | 12 | 12 | 12 | 11 | 12 | 12 | 12 | 11 | 12 | 11 | 12 | 11 |
| **11** | 14 | 14 | 14 | 14 | 14 | 14 | 13 | 13 | 14 | 12 |  | 13 | 13 | 14 | 14 | 14 | 14 | 14 | 13 | 13 | 13 | 12 | 13 | 12 | 13 | 11 | 13 | 13 | 14 | 12 |
| **12** | 13 | 13 | 13 | 13 | 13 | 13 | 13 | 13 | 13 | 12 | 13 |  | 13 | 13 | 13 | 13 | 13 | 13 | 13 | 13 | 13 | 12 | 13 | 12 | 13 | 11 | 12 | 12 | 13 | 11 |
| **13** | 13 | 13 | 13 | 13 | 13 | 13 | 13 | 13 | 13 | 12 | 13 | 13 |  | 13 | 13 | 13 | 13 | 13 | 13 | 13 | 13 | 12 | 13 | 12 | 13 | 11 | 12 | 12 | 13 | 11 |
| **14** | 14 | 14 | 14 | 14 | 14 | 14 | 13 | 13 | 14 | 12 | 14 | 13 | 13 |  | 14 | 14 | 14 | 14 | 13 | 13 | 13 | 12 | 13 | 12 | 13 | 11 | 13 | 13 | 14 | 12 |
| **15** | 14 | 14 | 14 | 14 | 14 | 14 | 13 | 13 | 14 | 12 | 14 | 13 | 13 | 14 |  | 14 | 14 | 14 | 13 | 13 | 13 | 12 | 13 | 12 | 13 | 11 | 13 | 13 | 14 | 12 |
| **16** | 14 | 14 | 14 | 14 | 14 | 14 | 13 | 13 | 14 | 12 | 14 | 13 | 13 | 14 | 14 |  | 14 | 14 | 13 | 13 | 13 | 12 | 13 | 12 | 13 | 11 | 13 | 13 | 14 | 12 |
| **17** | 14 | 14 | 14 | 14 | 14 | 14 | 13 | 13 | 14 | 12 | 14 | 13 | 13 | 14 | 14 | 14 |  | 14 | 13 | 13 | 13 | 12 | 13 | 12 | 13 | 11 | 13 | 13 | 14 | 12 |
| **18** | 14 | 14 | 14 | 14 | 14 | 14 | 13 | 13 | 14 | 12 | 14 | 13 | 13 | 14 | 14 | 14 | 14 |  | 13 | 13 | 13 | 12 | 13 | 12 | 13 | 11 | 13 | 13 | 14 | 12 |
| **19** | 13 | 13 | 13 | 13 | 13 | 13 | 13 | 13 | 13 | 12 | 13 | 13 | 13 | 13 | 13 | 13 | 13 | 13 |  | 13 | 13 | 12 | 13 | 12 | 13 | 11 | 12 | 12 | 13 | 11 |
| **20** | 13 | 13 | 13 | 13 | 13 | 13 | 13 | 13 | 13 | 12 | 13 | 13 | 13 | 13 | 13 | 13 | 13 | 13 | 13 |  | 13 | 12 | 13 | 12 | 13 | 11 | 12 | 12 | 13 | 11 |
| **21** | 13 | 13 | 13 | 13 | 13 | 13 | 13 | 13 | 13 | 12 | 13 | 13 | 13 | 13 | 13 | 13 | 13 | 13 | 13 | 13 |  | 12 | 13 | 12 | 13 | 11 | 12 | 12 | 13 | 11 |
| **22** | 12 | 12 | 12 | 12 | 12 | 12 | 12 | 12 | 12 | 11 | 12 | 12 | 12 | 12 | 12 | 12 | 12 | 12 | 12 | 12 | 12 |  | 12 | 11 | 12 | 10 | 11 | 11 | 12 | 10 |
| **23** | 13 | 13 | 13 | 13 | 13 | 13 | 13 | 13 | 13 | 12 | 13 | 13 | 13 | 13 | 13 | 13 | 13 | 13 | 13 | 13 | 13 | 12 |  | 12 | 13 | 11 | 12 | 12 | 13 | 11 |
| **24** | 12 | 12 | 12 | 12 | 12 | 12 | 12 | 12 | 12 | 12 | 12 | 12 | 12 | 12 | 12 | 12 | 12 | 12 | 12 | 12 | 12 | 11 | 12 |  | 12 | 11 | 12 | 11 | 12 | 11 |
| **25** | 13 | 13 | 13 | 13 | 13 | 13 | 13 | 13 | 13 | 12 | 13 | 13 | 13 | 13 | 13 | 13 | 13 | 13 | 13 | 13 | 13 | 12 | 13 | 12 |  | 11 | 12 | 12 | 13 | 11 |
| **26** | 11 | 11 | 11 | 11 | 11 | 11 | 11 | 11 | 11 | 11 | 11 | 11 | 11 | 11 | 11 | 11 | 11 | 11 | 11 | 11 | 11 | 10 | 11 | 11 | 11 |  | 11 | 10 | 11 | 10 |
| **27** | 13 | 13 | 13 | 13 | 13 | 13 | 12 | 12 | 13 | 12 | 13 | 12 | 12 | 13 | 13 | 13 | 13 | 13 | 12 | 12 | 12 | 11 | 12 | 12 | 12 | 11 |  | 11 | 13 | 12 |
| **28** | 13 | 13 | 13 | 13 | 13 | 13 | 12 | 12 | 13 | 11 | 13 | 12 | 12 | 13 | 13 | 13 | 13 | 13 | 12 | 12 | 12 | 11 | 12 | 11 | 12 | 10 | 11 |  | 13 | 12 |
| **29** | 14 | 14 | 14 | 14 | 14 | 14 | 13 | 13 | 14 | 12 | 14 | 13 | 13 | 14 | 14 | 14 | 14 | 14 | 13 | 13 | 13 | 12 | 13 | 12 | 13 | 11 | 13 | 13 |  | 12 |
| **30** | 0 | 14 | 14 | 14 | 14 | 14 | 13 | 13 | 14 | 12 | 14 | 13 | 13 | 14 | 14 | 14 | 14 | 14 | 13 | 13 | 13 | 12 | 13 | 12 | 13 | 11 | 13 | 13 | 14 |  |

channels

channels

**Supplementary Table 8** Degrees of freedom of the paired t-tests between 18 months and 36 months in each connection.

|  | **1** | **2** | **3** | **4** | **5** | **6** | **7** | **8** | **9** | **10** | **11** | **12** | **13** | **14** | **15** | **16** | **17** | **18** | **19** | **20** | **21** | **22** | **23** | **24** | **25** | **26** | **27** | **28** | **29** | **30** | **31** | **32** | **33** | **34** | **35** | **36** | **37** | **38** | **39** | **40** | **41** | **42** | **43** | **44** |
| --- | --- | --- | --- | --- | --- | --- | --- | --- | --- | --- | --- | --- | --- | --- | --- | --- | --- | --- | --- | --- | --- | --- | --- | --- | --- | --- | --- | --- | --- | --- | --- | --- | --- | --- | --- | --- | --- | --- | --- | --- | --- | --- | --- | --- |
| **1** |  | 9 | 9 | 8 | 8 | 9 | 9 | 9 | 9 | 7 | 9 | 7 | 9 | 9 | 9 | 9 | 8 | 8 | 8 | 9 | 8 | 9 | 9 | 9 | 9 | 9 | 9 | 9 | 9 | 8 | 9 | 9 | 9 | 9 | 9 | 9 | 8 | 9 | 9 | 9 | 9 | 9 | 8 | 8 |
| **2** | 9 |  | 14 | 8 | 8 | 14 | 13 | 13 | 14 | 12 | 10 | 12 | 10 | 14 | 14 | 14 | 13 | 13 | 12 | 14 | 13 | 14 | 14 | 14 | 14 | 14 | 14 | 14 | 13 | 12 | 14 | 13 | 14 | 13 | 13 | 12 | 12 | 11 | 11 | 13 | 13 | 14 | 11 | 12 |
| **3** | 9 | 14 |  | 8 | 8 | 14 | 13 | 13 | 14 | 12 | 10 | 12 | 10 | 14 | 14 | 14 | 13 | 13 | 12 | 14 | 13 | 14 | 14 | 14 | 14 | 14 | 14 | 14 | 13 | 12 | 14 | 13 | 14 | 13 | 13 | 12 | 12 | 11 | 11 | 13 | 13 | 14 | 11 | 12 |
| **4** | 8 | 8 | 8 |  | 8 | 8 | 8 | 8 | 8 | 7 | 9 | 9 | 9 | 8 | 8 | 8 | 10 | 10 | 10 | 8 | 10 | 8 | 8 | 8 | 8 | 8 | 8 | 8 | 8 | 10 | 8 | 8 | 8 | 8 | 8 | 8 | 10 | 9 | 9 | 8 | 8 | 8 | 10 | 10 |
| **5** | 8 | 8 | 8 | 8 |  | 8 | 8 | 8 | 8 | 10 | 9 | 9 | 9 | 8 | 8 | 8 | 10 | 10 | 10 | 8 | 10 | 8 | 8 | 8 | 8 | 8 | 8 | 8 | 8 | 10 | 8 | 8 | 8 | 8 | 8 | 8 | 10 | 9 | 9 | 8 | 8 | 8 | 10 | 10 |
| **6** | 9 | 14 | 14 | 8 | 8 |  | 13 | 13 | 14 | 12 | 10 | 12 | 10 | 14 | 14 | 14 | 13 | 13 | 12 | 14 | 13 | 14 | 14 | 14 | 14 | 14 | 14 | 14 | 13 | 12 | 14 | 13 | 14 | 13 | 13 | 12 | 12 | 11 | 11 | 13 | 13 | 14 | 11 | 12 |
| **7** | 9 | 13 | 13 | 8 | 8 | 13 |  | 12 | 13 | 11 | 10 | 11 | 10 | 13 | 13 | 13 | 12 | 12 | 11 | 13 | 12 | 13 | 13 | 13 | 13 | 13 | 13 | 13 | 12 | 11 | 13 | 12 | 13 | 13 | 13 | 12 | 11 | 10 | 10 | 12 | 12 | 13 | 10 | 11 |
| **8** | 9 | 13 | 13 | 8 | 8 | 13 | 12 |  | 13 | 11 | 9 | 11 | 9 | 13 | 13 | 13 | 12 | 12 | 12 | 13 | 12 | 13 | 13 | 13 | 13 | 13 | 13 | 13 | 12 | 11 | 13 | 13 | 13 | 12 | 12 | 11 | 11 | 10 | 10 | 13 | 13 | 13 | 11 | 12 |
| **9** | 9 | 14 | 14 | 8 | 8 | 14 | 13 | 13 |  | 12 | 10 | 12 | 10 | 14 | 14 | 14 | 13 | 13 | 12 | 14 | 13 | 14 | 14 | 14 | 14 | 14 | 14 | 14 | 13 | 12 | 14 | 13 | 14 | 13 | 13 | 12 | 12 | 11 | 11 | 13 | 13 | 14 | 11 | 12 |
| **10** | 10 | 12 | 12 | 10 | 10 | 12 | 11 | 11 | 12 |  | 10 | 11 | 10 | 12 | 12 | 12 | 11 | 11 | 10 | 12 | 11 | 12 | 12 | 12 | 12 | 12 | 12 | 12 | 11 | 10 | 12 | 11 | 12 | 11 | 11 | 10 | 11 | 10 | 10 | 11 | 11 | 12 | 10 | 11 |
| **11** | 9 | 10 | 10 | 9 | 9 | 10 | 10 | 9 | 10 | 10 |  | 9 | 10 | 10 | 10 | 10 | 10 | 10 | 9 | 10 | 10 | 10 | 10 | 10 | 10 | 10 | 10 | 10 | 9 | 9 | 10 | 9 | 10 | 10 | 10 | 9 | 9 | 9 | 9 | 9 | 9 | 10 | 8 | 9 |
| **12** | 10 | 12 | 12 | 9 | 9 | 12 | 11 | 11 | 12 | 11 | 9 |  | 9 | 12 | 12 | 12 | 11 | 11 | 10 | 12 | 11 | 12 | 12 | 12 | 12 | 12 | 12 | 12 | 11 | 10 | 12 | 11 | 12 | 11 | 11 | 10 | 11 | 9 | 9 | 11 | 11 | 12 | 10 | 11 |
| **13** | 9 | 10 | 10 | 9 | 9 | 10 | 10 | 9 | 10 | 10 | 10 | 9 | 0 | 10 | 10 | 10 | 10 | 10 | 9 | 10 | 10 | 10 | 10 | 10 | 10 | 10 | 10 | 10 | 9 | 9 | 10 | 9 | 10 | 10 | 10 | 9 | 9 | 9 | 9 | 9 | 9 | 10 | 8 | 9 |
| **14** | 9 | 14 | 14 | 8 | 8 | 14 | 13 | 13 | 14 | 12 | 10 | 12 | 10 |  | 14 | 14 | 13 | 13 | 12 | 14 | 13 | 14 | 14 | 14 | 14 | 14 | 14 | 14 | 13 | 12 | 14 | 13 | 14 | 13 | 13 | 12 | 12 | 11 | 11 | 13 | 13 | 14 | 11 | 12 |
| **15** | 9 | 14 | 14 | 8 | 8 | 14 | 13 | 13 | 14 | 12 | 10 | 12 | 10 | 14 |  | 14 | 13 | 13 | 12 | 14 | 13 | 14 | 14 | 14 | 14 | 14 | 14 | 14 | 13 | 12 | 14 | 13 | 14 | 13 | 13 | 12 | 12 | 11 | 11 | 13 | 13 | 14 | 11 | 12 |
| **16** | 9 | 14 | 14 | 8 | 8 | 14 | 13 | 13 | 14 | 12 | 10 | 12 | 10 | 14 | 14 |  | 13 | 13 | 12 | 14 | 13 | 14 | 14 | 14 | 14 | 14 | 14 | 14 | 13 | 12 | 14 | 13 | 14 | 13 | 13 | 12 | 12 | 11 | 11 | 13 | 13 | 14 | 11 | 12 |
| **17** | 8 | 13 | 13 | 10 | 10 | 13 | 12 | 12 | 13 | 11 | 10 | 11 | 10 | 13 | 13 | 13 |  | 13 | 12 | 13 | 13 | 13 | 13 | 13 | 13 | 13 | 13 | 13 | 12 | 12 | 13 | 12 | 13 | 12 | 12 | 11 | 11 | 11 | 11 | 12 | 12 | 13 | 10 | 11 |
| **18** | 8 | 13 | 13 | 10 | 10 | 13 | 12 | 12 | 13 | 11 | 10 | 11 | 10 | 13 | 13 | 13 | 13 |  | 12 | 13 | 13 | 13 | 13 | 13 | 13 | 13 | 13 | 13 | 12 | 12 | 13 | 12 | 13 | 12 | 12 | 11 | 11 | 11 | 11 | 12 | 12 | 13 | 10 | 11 |
| **19** | 8 | 12 | 12 | 10 | 10 | 12 | 11 | 12 | 12 | 10 | 9 | 10 | 9 | 12 | 12 | 12 | 12 | 12 |  | 12 | 12 | 12 | 12 | 12 | 12 | 12 | 12 | 12 | 11 | 11 | 12 | 12 | 12 | 11 | 11 | 10 | 10 | 10 | 10 | 12 | 12 | 12 | 10 | 11 |
| **20** | 9 | 14 | 14 | 8 | 8 | 14 | 13 | 13 | 14 | 12 | 10 | 12 | 10 | 14 | 14 | 14 | 13 | 13 | 12 |  | 13 | 14 | 14 | 14 | 14 | 14 | 14 | 14 | 13 | 12 | 14 | 13 | 14 | 13 | 13 | 12 | 12 | 11 | 11 | 13 | 13 | 14 | 11 | 12 |
| **21** | 8 | 13 | 13 | 10 | 10 | 13 | 12 | 12 | 13 | 11 | 10 | 11 | 10 | 13 | 13 | 13 | 13 | 13 | 12 | 13 |  | 13 | 13 | 13 | 13 | 13 | 13 | 13 | 12 | 12 | 13 | 12 | 13 | 12 | 12 | 11 | 11 | 11 | 11 | 12 | 12 | 13 | 10 | 11 |
| **22** | 9 | 14 | 14 | 8 | 8 | 14 | 13 | 13 | 14 | 12 | 10 | 12 | 10 | 14 | 14 | 14 | 13 | 13 | 12 | 14 | 13 |  | 14 | 14 | 14 | 14 | 14 | 14 | 13 | 12 | 14 | 13 | 14 | 13 | 13 | 12 | 12 | 11 | 11 | 13 | 13 | 14 | 11 | 12 |
| **23** | 9 | 14 | 14 | 8 | 8 | 14 | 13 | 13 | 14 | 12 | 10 | 12 | 10 | 14 | 14 | 14 | 13 | 13 | 12 | 14 | 13 | 14 |  | 14 | 14 | 14 | 14 | 14 | 13 | 12 | 14 | 13 | 14 | 13 | 13 | 12 | 12 | 11 | 11 | 13 | 13 | 14 | 11 | 12 |
| **24** | 9 | 14 | 14 | 8 | 8 | 14 | 13 | 13 | 14 | 12 | 10 | 12 | 10 | 14 | 14 | 14 | 13 | 13 | 12 | 14 | 13 | 14 | 14 |  | 14 | 14 | 14 | 14 | 13 | 12 | 14 | 13 | 14 | 13 | 13 | 12 | 12 | 11 | 11 | 13 | 13 | 14 | 11 | 12 |
| **25** | 9 | 14 | 14 | 8 | 8 | 14 | 13 | 13 | 14 | 12 | 10 | 12 | 10 | 14 | 14 | 14 | 13 | 13 | 12 | 14 | 13 | 14 | 14 | 14 |  | 14 | 14 | 14 | 13 | 12 | 14 | 13 | 14 | 13 | 13 | 12 | 12 | 11 | 11 | 13 | 13 | 14 | 11 | 12 |
| **29** | 9 | 14 | 14 | 8 | 8 | 14 | 13 | 13 | 14 | 12 | 10 | 12 | 10 | 14 | 14 | 14 | 13 | 13 | 12 | 14 | 13 | 14 | 14 | 14 | 14 |  | 14 | 14 | 13 | 12 | 14 | 13 | 14 | 13 | 13 | 12 | 12 | 11 | 11 | 13 | 13 | 14 | 11 | 12 |
| **27** | 9 | 14 | 14 | 8 | 8 | 14 | 13 | 13 | 14 | 12 | 10 | 12 | 10 | 14 | 14 | 14 | 13 | 13 | 12 | 14 | 13 | 14 | 14 | 14 | 14 | 14 |  | 12 | 13 | 12 | 14 | 13 | 14 | 13 | 13 | 12 | 12 | 11 | 11 | 13 | 13 | 14 | 11 | 12 |
| **28** | 9 | 14 | 14 | 8 | 8 | 14 | 13 | 13 | 14 | 12 | 10 | 12 | 10 | 14 | 14 | 14 | 13 | 13 | 12 | 14 | 13 | 14 | 14 | 14 | 14 | 14 | 12 |  | 13 | 12 | 14 | 13 | 14 | 13 | 13 | 12 | 12 | 11 | 11 | 13 | 13 | 14 | 11 | 12 |
| **29** | 9 | 13 | 13 | 8 | 8 | 13 | 12 | 12 | 13 | 11 | 9 | 11 | 9 | 13 | 13 | 13 | 12 | 12 | 11 | 13 | 12 | 13 | 13 | 13 | 13 | 13 | 13 | 13 |  | 12 | 13 | 12 | 13 | 12 | 12 | 11 | 11 | 10 | 10 | 12 | 12 | 13 | 11 | 11 |
| **30** | 8 | 12 | 12 | 10 | 10 | 12 | 11 | 11 | 12 | 10 | 9 | 10 | 9 | 12 | 12 | 12 | 12 | 12 | 11 | 12 | 12 | 12 | 12 | 12 | 12 | 12 | 12 | 12 | 12 | 0 | 12 | 11 | 12 | 11 | 11 | 10 | 10 | 10 | 10 | 11 | 11 | 12 | 10 | 10 |
| **31** | 9 | 14 | 14 | 8 | 8 | 14 | 13 | 13 | 14 | 12 | 10 | 12 | 10 | 14 | 14 | 14 | 13 | 13 | 12 | 14 | 13 | 14 | 14 | 14 | 14 | 14 | 14 | 14 | 13 | 12 |  | 13 | 14 | 13 | 13 | 12 | 12 | 11 | 11 | 13 | 13 | 14 | 11 | 12 |
| **32** | 9 | 13 | 13 | 8 | 8 | 13 | 12 | 13 | 13 | 11 | 9 | 11 | 9 | 13 | 13 | 13 | 12 | 12 | 12 | 13 | 12 | 13 | 13 | 13 | 13 | 13 | 13 | 13 | 12 | 11 | 13 |  | 13 | 12 | 12 | 11 | 11 | 10 | 10 | 13 | 13 | 13 | 11 | 12 |
| **33** | 9 | 14 | 14 | 8 | 8 | 14 | 13 | 13 | 14 | 12 | 10 | 12 | 10 | 14 | 14 | 14 | 13 | 13 | 12 | 14 | 13 | 14 | 14 | 14 | 14 | 14 | 14 | 14 | 13 | 12 | 14 | 13 |  | 13 | 13 | 12 | 12 | 11 | 11 | 13 | 13 | 14 | 11 | 12 |
| **34** | 9 | 13 | 13 | 8 | 8 | 13 | 13 | 12 | 13 | 11 | 10 | 11 | 10 | 13 | 13 | 13 | 12 | 12 | 11 | 13 | 12 | 13 | 13 | 13 | 13 | 13 | 13 | 13 | 12 | 11 | 13 | 12 | 13 |  | 13 | 12 | 11 | 10 | 10 | 12 | 12 | 13 | 10 | 11 |
| **35** | 9 | 13 | 13 | 8 | 8 | 13 | 13 | 12 | 13 | 11 | 10 | 11 | 10 | 13 | 13 | 13 | 12 | 12 | 11 | 13 | 12 | 13 | 13 | 13 | 13 | 13 | 13 | 13 | 12 | 11 | 13 | 12 | 13 | 13 |  | 12 | 11 | 10 | 10 | 12 | 12 | 13 | 10 | 11 |
| **36** | 9 | 12 | 12 | 8 | 8 | 12 | 12 | 11 | 12 | 10 | 9 | 10 | 9 | 12 | 12 | 12 | 11 | 11 | 10 | 12 | 11 | 12 | 12 | 12 | 12 | 12 | 12 | 12 | 11 | 10 | 12 | 11 | 12 | 12 | 12 |  | 11 | 9 | 9 | 11 | 11 | 12 | 9 | 10 |
| **37** | 8 | 12 | 12 | 10 | 10 | 12 | 11 | 11 | 12 | 11 | 9 | 11 | 9 | 12 | 12 | 12 | 11 | 11 | 10 | 12 | 11 | 12 | 12 | 12 | 12 | 12 | 12 | 12 | 11 | 10 | 12 | 11 | 12 | 11 | 11 | 11 |  | 9 | 9 | 11 | 11 | 12 | 10 | 11 |
| **38** | 9 | 11 | 11 | 9 | 9 | 11 | 10 | 10 | 11 | 10 | 9 | 9 | 9 | 11 | 11 | 11 | 11 | 11 | 10 | 11 | 11 | 11 | 11 | 11 | 11 | 11 | 11 | 11 | 10 | 10 | 11 | 10 | 11 | 10 | 10 | 9 | 9 |  | 11 | 10 | 10 | 11 | 8 | 9 |
| **39** | 9 | 11 | 11 | 9 | 9 | 11 | 10 | 10 | 11 | 10 | 9 | 9 | 9 | 11 | 11 | 11 | 11 | 11 | 10 | 11 | 11 | 11 | 11 | 11 | 11 | 11 | 11 | 11 | 10 | 10 | 11 | 10 | 11 | 10 | 10 | 9 | 9 | 11 |  | 10 | 10 | 11 | 8 | 9 |
| **40** | 9 | 13 | 13 | 8 | 8 | 13 | 12 | 13 | 13 | 11 | 9 | 11 | 9 | 13 | 13 | 13 | 12 | 12 | 12 | 13 | 12 | 13 | 13 | 13 | 13 | 13 | 13 | 13 | 12 | 11 | 13 | 13 | 13 | 12 | 12 | 11 | 11 | 10 | 10 |  | 13 | 13 | 11 | 12 |
| **41** | 9 | 13 | 13 | 8 | 8 | 13 | 12 | 13 | 13 | 11 | 9 | 11 | 9 | 13 | 13 | 13 | 12 | 12 | 12 | 13 | 12 | 13 | 13 | 13 | 13 | 13 | 13 | 13 | 12 | 11 | 13 | 13 | 13 | 12 | 12 | 11 | 11 | 10 | 10 | 13 |  | 13 | 11 | 12 |
| **42** | 9 | 14 | 14 | 8 | 8 | 14 | 13 | 13 | 14 | 12 | 10 | 12 | 10 | 14 | 14 | 14 | 13 | 13 | 12 | 14 | 13 | 14 | 14 | 14 | 14 | 14 | 14 | 14 | 13 | 12 | 14 | 13 | 14 | 13 | 13 | 12 | 12 | 11 | 11 | 13 | 13 |  | 11 | 12 |
| **43** | 8 | 11 | 11 | 10 | 10 | 11 | 10 | 11 | 11 | 10 | 8 | 10 | 8 | 11 | 11 | 11 | 10 | 10 | 10 | 11 | 10 | 11 | 11 | 11 | 11 | 11 | 11 | 11 | 11 | 10 | 11 | 11 | 11 | 10 | 10 | 9 | 10 | 8 | 8 | 11 | 11 | 11 |  | 11 |
| **44** | 8 | 12 | 12 | 10 | 10 | 12 | 11 | 12 | 12 | 11 | 9 | 11 | 9 | 12 | 12 | 12 | 11 | 11 | 11 | 12 | 11 | 12 | 12 | 12 | 12 | 12 | 12 | 12 | 11 | 10 | 12 | 12 | 12 | 11 | 11 | 10 | 11 | 9 | 9 | 12 | 12 | 12 | 11 |  |

channels

channels

channels

**Supplementary Table 9** Degrees of freedom of the paired t-tests between 24 months and 30 months in each connection.

|  | **1** | **2** | **3** | **4** | **5** | **6** | **7** | **8** | **9** | **10** | **11** | **12** | **13** | **14** | **15** | **16** | **17** | **18** | **19** | **20** | **21** | **22** | **23** | **24** | **25** | **26** | **27** | **28** | **29** | **30** | **31** | **32** | **33** | **34** | **35** | **36** | **37** | **38** | **39** | **40** | **41** | **42** | **43** | **44** |
| --- | --- | --- | --- | --- | --- | --- | --- | --- | --- | --- | --- | --- | --- | --- | --- | --- | --- | --- | --- | --- | --- | --- | --- | --- | --- | --- | --- | --- | --- | --- | --- | --- | --- | --- | --- | --- | --- | --- | --- | --- | --- | --- | --- | --- |
| **1** |  | 13 | 13 | 11 | 11 | 13 | 13 | 12 | 12 | 10 | 10 | 13 | 11 | 13 | 13 | 13 | 12 | 13 | 11 | 12 | 13 | 13 | 13 | 13 | 13 | 13 | 13 | 13 | 13 | 12 | 13 | 13 | 13 | 13 | 12 | 13 | 13 | 9 | 9 | 12 | 10 | 12 | 11 | 13 |
| **2** | 13 |  | 17 | 11 | 11 | 16 | 16 | 15 | 15 | 14 | 14 | 17 | 15 | 17 | 17 | 16 | 15 | 17 | 14 | 16 | 17 | 17 | 17 | 17 | 17 | 17 | 17 | 17 | 16 | 15 | 17 | 16 | 16 | 16 | 16 | 16 | 16 | 13 | 13 | 15 | 13 | 16 | 13 | 17 |
| **3** | 13 | 17 |  | 11 | 11 | 16 | 16 | 15 | 15 | 14 | 14 | 17 | 15 | 17 | 17 | 16 | 15 | 17 | 14 | 16 | 17 | 17 | 17 | 17 | 17 | 17 | 17 | 17 | 16 | 15 | 17 | 16 | 16 | 16 | 16 | 16 | 16 | 13 | 13 | 15 | 13 | 16 | 13 | 17 |
| **4** | 11 | 11 | 11 |  | 11 | 11 | 11 | 10 | 10 | 9 | 9 | 11 | 10 | 11 | 11 | 11 | 10 | 11 | 9 | 10 | 11 | 11 | 11 | 11 | 11 | 11 | 11 | 11 | 11 | 10 | 11 | 11 | 11 | 11 | 10 | 11 | 11 | 11 | 11 | 10 | 11 | 10 | 9 | 11 |
| **5** | 11 | 11 | 11 | 11 |  | 11 | 11 | 10 | 10 | 9 | 9 | 11 | 10 | 11 | 11 | 11 | 10 | 11 | 9 | 10 | 11 | 11 | 11 | 11 | 11 | 11 | 11 | 11 | 11 | 10 | 11 | 11 | 11 | 11 | 10 | 11 | 11 | 11 | 11 | 10 | 11 | 10 | 9 | 11 |
| **6** | 13 | 16 | 16 | 11 | 11 |  | 16 | 15 | 15 | 13 | 13 | 16 | 14 | 16 | 16 | 16 | 15 | 16 | 14 | 15 | 16 | 16 | 16 | 16 | 16 | 16 | 16 | 16 | 16 | 15 | 16 | 16 | 16 | 16 | 15 | 15 | 15 | 12 | 12 | 15 | 13 | 15 | 13 | 16 |
| **7** | 13 | 16 | 16 | 11 | 11 | 16 |  | 15 | 15 | 13 | 13 | 16 | 14 | 16 | 16 | 16 | 15 | 16 | 14 | 15 | 16 | 16 | 16 | 16 | 16 | 16 | 16 | 16 | 16 | 15 | 16 | 16 | 16 | 16 | 15 | 15 | 15 | 12 | 12 | 15 | 13 | 15 | 13 | 16 |
| **8** | 12 | 15 | 15 | 10 | 10 | 15 | 15 |  | 14 | 12 | 13 | 15 | 13 | 15 | 15 | 15 | 15 | 15 | 14 | 14 | 15 | 15 | 15 | 15 | 15 | 15 | 15 | 15 | 15 | 14 | 15 | 15 | 15 | 15 | 14 | 14 | 14 | 12 | 12 | 14 | 12 | 14 | 12 | 15 |
| **9** | 12 | 15 | 15 | 10 | 10 | 15 | 15 | 14 |  | 13 | 13 | 15 | 14 | 15 | 15 | 15 | 14 | 15 | 13 | 14 | 15 | 15 | 15 | 15 | 15 | 15 | 15 | 15 | 15 | 14 | 15 | 15 | 15 | 15 | 15 | 14 | 14 | 11 | 11 | 14 | 12 | 14 | 13 | 15 |
| **10** | 10 | 14 | 14 | 9 | 9 | 13 | 13 | 12 | 13 |  | 13 | 14 | 14 | 14 | 14 | 13 | 12 | 14 | 11 | 13 | 14 | 14 | 14 | 14 | 14 | 14 | 14 | 14 | 13 | 12 | 14 | 13 | 13 | 13 | 14 | 13 | 13 | 11 | 11 | 12 | 11 | 14 | 12 | 14 |
| **11** | 10 | 14 | 14 | 9 | 9 | 13 | 13 | 13 | 13 | 13 |  | 14 | 14 | 14 | 14 | 13 | 13 | 14 | 12 | 13 | 14 | 14 | 14 | 14 | 14 | 14 | 14 | 14 | 13 | 12 | 14 | 13 | 13 | 13 | 14 | 13 | 13 | 12 | 12 | 12 | 10 | 13 | 11 | 14 |
| **12** | 13 | 17 | 17 | 11 | 11 | 16 | 16 | 15 | 15 | 14 | 14 |  | 15 | 17 | 17 | 16 | 15 | 17 | 14 | 16 | 17 | 17 | 17 | 17 | 17 | 17 | 17 | 17 | 16 | 15 | 17 | 16 | 16 | 16 | 16 | 16 | 16 | 13 | 13 | 15 | 13 | 16 | 13 | 17 |
| **13** | 11 | 15 | 15 | 10 | 10 | 14 | 14 | 13 | 14 | 14 | 14 | 15 |  | 15 | 15 | 14 | 13 | 15 | 12 | 14 | 15 | 15 | 15 | 15 | 15 | 15 | 15 | 15 | 14 | 13 | 15 | 14 | 14 | 14 | 15 | 14 | 14 | 12 | 12 | 13 | 11 | 14 | 12 | 15 |
| **14** | 13 | 17 | 17 | 11 | 11 | 16 | 16 | 15 | 15 | 14 | 14 | 17 | 15 |  | 17 | 16 | 15 | 17 | 14 | 16 | 17 | 17 | 17 | 17 | 17 | 17 | 17 | 17 | 16 | 15 | 17 | 16 | 16 | 16 | 16 | 16 | 16 | 13 | 13 | 15 | 13 | 16 | 13 | 17 |
| **15** | 13 | 17 | 17 | 11 | 11 | 16 | 16 | 15 | 15 | 14 | 14 | 17 | 15 | 17 |  | 16 | 15 | 17 | 14 | 16 | 17 | 17 | 17 | 17 | 17 | 17 | 17 | 17 | 16 | 15 | 17 | 16 | 16 | 16 | 16 | 16 | 16 | 13 | 13 | 15 | 13 | 16 | 13 | 17 |
| **16** | 13 | 16 | 16 | 11 | 11 | 16 | 16 | 15 | 15 | 13 | 13 | 16 | 14 | 16 | 16 |  | 15 | 16 | 14 | 15 | 16 | 16 | 16 | 16 | 16 | 16 | 16 | 16 | 16 | 15 | 16 | 16 | 16 | 16 | 15 | 15 | 15 | 12 | 12 | 15 | 13 | 15 | 13 | 16 |
| **17** | 12 | 15 | 15 | 10 | 10 | 15 | 15 | 15 | 14 | 12 | 13 | 15 | 13 | 15 | 15 | 15 |  | 15 | 14 | 14 | 15 | 15 | 15 | 15 | 15 | 15 | 15 | 15 | 15 | 14 | 15 | 15 | 15 | 15 | 14 | 14 | 14 | 12 | 12 | 14 | 12 | 14 | 12 | 15 |
| **18** | 13 | 17 | 17 | 11 | 11 | 16 | 16 | 15 | 15 | 14 | 14 | 17 | 15 | 17 | 17 | 16 | 15 |  | 14 | 16 | 17 | 17 | 17 | 17 | 17 | 17 | 17 | 17 | 16 | 15 | 17 | 16 | 16 | 16 | 16 | 16 | 16 | 13 | 13 | 15 | 13 | 16 | 13 | 17 |
| **19** | 11 | 14 | 14 | 9 | 9 | 14 | 14 | 14 | 13 | 11 | 12 | 14 | 12 | 14 | 14 | 14 | 14 | 14 |  | 14 | 14 | 14 | 14 | 14 | 14 | 14 | 14 | 14 | 14 | 13 | 14 | 14 | 14 | 14 | 13 | 13 | 13 | 12 | 12 | 14 | 12 | 13 | 11 | 14 |
| **20** | 12 | 16 | 16 | 10 | 10 | 15 | 15 | 14 | 14 | 13 | 13 | 16 | 14 | 16 | 16 | 15 | 14 | 16 | 14 |  | 16 | 16 | 16 | 16 | 16 | 16 | 16 | 16 | 15 | 14 | 16 | 15 | 15 | 15 | 15 | 15 | 15 | 13 | 13 | 15 | 13 | 15 | 12 | 16 |
| **21**  channels | 13 | 17 | 17 | 11 | 11 | 16 | 16 | 15 | 15 | 14 | 14 | 17 | 15 | 17 | 17 | 16 | 15 | 17 | 14 | 16 |  | 17 | 17 | 17 | 17 | 17 | 17 | 17 | 16 | 15 | 17 | 16 | 16 | 16 | 16 | 16 | 16 | 13 | 13 | 15 | 13 | 16 | 13 | 17 |
| **22** | 13 | 17 | 17 | 11 | 11 | 16 | 16 | 15 | 15 | 14 | 14 | 17 | 15 | 17 | 17 | 16 | 15 | 17 | 14 | 16 | 17 |  | 17 | 17 | 17 | 17 | 17 | 17 | 16 | 15 | 17 | 16 | 16 | 16 | 16 | 16 | 16 | 13 | 13 | 15 | 13 | 16 | 13 | 17 |
| **23** | 13 | 17 | 17 | 11 | 11 | 16 | 16 | 15 | 15 | 14 | 14 | 17 | 15 | 17 | 17 | 16 | 15 | 17 | 14 | 16 | 17 | 17 |  | 17 | 17 | 17 | 17 | 17 | 16 | 15 | 17 | 16 | 16 | 16 | 16 | 16 | 16 | 13 | 13 | 15 | 13 | 16 | 13 | 17 |
| **24** | 13 | 17 | 17 | 11 | 11 | 16 | 16 | 15 | 15 | 14 | 14 | 17 | 15 | 17 | 17 | 16 | 15 | 17 | 14 | 16 | 17 | 17 | 17 |  | 17 | 17 | 17 | 17 | 16 | 15 | 17 | 16 | 16 | 16 | 16 | 16 | 16 | 13 | 13 | 15 | 13 | 16 | 13 | 17 |
| **25** | 13 | 17 | 17 | 11 | 11 | 16 | 16 | 15 | 15 | 14 | 14 | 17 | 15 | 17 | 17 | 16 | 15 | 17 | 14 | 16 | 17 | 17 | 17 | 17 |  | 17 | 17 | 17 | 16 | 15 | 17 | 16 | 16 | 16 | 16 | 16 | 16 | 13 | 13 | 15 | 13 | 16 | 13 | 17 |
| **26** | 13 | 17 | 17 | 11 | 11 | 16 | 16 | 15 | 15 | 14 | 14 | 17 | 15 | 17 | 17 | 16 | 15 | 17 | 14 | 16 | 17 | 17 | 17 | 17 | 17 |  | 17 | 17 | 16 | 15 | 17 | 16 | 16 | 16 | 16 | 16 | 16 | 13 | 13 | 15 | 13 | 16 | 13 | 17 |
| **27** | 13 | 17 | 17 | 11 | 11 | 16 | 16 | 15 | 15 | 14 | 14 | 17 | 15 | 17 | 17 | 16 | 15 | 17 | 14 | 16 | 17 | 17 | 17 | 17 | 17 | 17 |  | 15 | 16 | 15 | 17 | 16 | 16 | 16 | 16 | 16 | 16 | 13 | 13 | 15 | 13 | 16 | 13 | 17 |
| **28** | 13 | 17 | 17 | 11 | 11 | 16 | 16 | 15 | 15 | 14 | 14 | 17 | 15 | 17 | 17 | 16 | 15 | 17 | 14 | 16 | 17 | 17 | 17 | 17 | 17 | 17 | 15 |  | 16 | 15 | 17 | 16 | 16 | 16 | 16 | 16 | 16 | 13 | 13 | 15 | 13 | 16 | 13 | 17 |
| **29** | 13 | 16 | 16 | 11 | 11 | 16 | 16 | 15 | 15 | 13 | 13 | 16 | 14 | 16 | 16 | 16 | 15 | 16 | 14 | 15 | 16 | 16 | 16 | 16 | 16 | 16 | 16 | 16 |  | 15 | 16 | 16 | 16 | 16 | 15 | 15 | 15 | 12 | 12 | 15 | 13 | 15 | 13 | 16 |
| **30** | 12 | 15 | 15 | 10 | 10 | 15 | 15 | 14 | 14 | 12 | 12 | 15 | 13 | 15 | 15 | 15 | 14 | 15 | 13 | 14 | 15 | 15 | 15 | 15 | 15 | 15 | 15 | 15 | 15 |  | 15 | 15 | 15 | 15 | 14 | 14 | 14 | 11 | 11 | 14 | 13 | 14 | 12 | 15 |
| **31** | 13 | 17 | 17 | 11 | 11 | 16 | 16 | 15 | 15 | 14 | 14 | 17 | 15 | 17 | 17 | 16 | 15 | 17 | 14 | 16 | 17 | 17 | 17 | 17 | 17 | 17 | 17 | 17 | 16 | 15 |  | 16 | 16 | 16 | 16 | 16 | 16 | 13 | 13 | 15 | 13 | 16 | 13 | 17 |
| **32** | 13 | 16 | 16 | 11 | 11 | 16 | 16 | 15 | 15 | 13 | 13 | 16 | 14 | 16 | 16 | 16 | 15 | 16 | 14 | 15 | 16 | 16 | 16 | 16 | 16 | 16 | 16 | 16 | 16 | 15 | 16 |  | 16 | 16 | 15 | 15 | 15 | 12 | 12 | 15 | 13 | 15 | 13 | 16 |
| **33** | 13 | 16 | 16 | 11 | 11 | 16 | 16 | 15 | 15 | 13 | 13 | 16 | 14 | 16 | 16 | 16 | 15 | 16 | 14 | 15 | 16 | 16 | 16 | 16 | 16 | 16 | 16 | 16 | 16 | 15 | 16 | 16 |  | 16 | 15 | 15 | 15 | 12 | 12 | 15 | 13 | 15 | 13 | 16 |
| **34** | 13 | 16 | 16 | 11 | 11 | 16 | 16 | 15 | 15 | 13 | 13 | 16 | 14 | 16 | 16 | 16 | 15 | 16 | 14 | 15 | 16 | 16 | 16 | 16 | 16 | 16 | 16 | 16 | 16 | 15 | 16 | 16 | 16 |  | 15 | 15 | 15 | 12 | 12 | 15 | 13 | 15 | 13 | 16 |
| **35** | 12 | 16 | 16 | 10 | 10 | 15 | 15 | 14 | 15 | 14 | 14 | 16 | 15 | 16 | 16 | 15 | 14 | 16 | 13 | 15 | 16 | 16 | 16 | 16 | 16 | 16 | 16 | 16 | 15 | 14 | 16 | 15 | 15 | 15 |  | 15 | 15 | 12 | 12 | 14 | 12 | 15 | 13 | 16 |
| **36** | 13 | 16 | 16 | 11 | 11 | 15 | 15 | 14 | 14 | 13 | 13 | 16 | 14 | 16 | 16 | 15 | 14 | 16 | 13 | 15 | 16 | 16 | 16 | 16 | 16 | 16 | 16 | 16 | 15 | 14 | 16 | 15 | 15 | 15 | 15 |  | 16 | 12 | 12 | 14 | 12 | 15 | 12 | 16 |
| **37** | 13 | 16 | 16 | 11 | 11 | 15 | 15 | 14 | 14 | 13 | 13 | 16 | 14 | 16 | 16 | 15 | 14 | 16 | 13 | 15 | 16 | 16 | 16 | 16 | 16 | 16 | 16 | 16 | 15 | 14 | 16 | 15 | 15 | 15 | 15 | 16 |  | 12 | 12 | 14 | 12 | 15 | 12 | 16 |
| **38** | 9 | 13 | 13 | 11 | 11 | 12 | 12 | 12 | 11 | 11 | 12 | 13 | 12 | 13 | 13 | 12 | 12 | 13 | 12 | 13 | 13 | 13 | 13 | 13 | 13 | 13 | 13 | 13 | 12 | 11 | 13 | 12 | 12 | 12 | 12 | 12 | 12 |  | 13 | 12 | 10 | 12 | 9 | 13 |
| **39** | 9 | 13 | 13 | 11 | 11 | 12 | 12 | 12 | 11 | 11 | 12 | 13 | 12 | 13 | 13 | 12 | 12 | 13 | 12 | 13 | 13 | 13 | 13 | 13 | 13 | 13 | 13 | 13 | 12 | 11 | 13 | 12 | 12 | 12 | 12 | 12 | 12 | 13 |  | 12 | 10 | 12 | 9 | 13 |
| **40** | 12 | 15 | 15 | 10 | 10 | 15 | 15 | 14 | 14 | 12 | 12 | 15 | 13 | 15 | 15 | 15 | 14 | 15 | 14 | 15 | 15 | 15 | 15 | 15 | 15 | 15 | 15 | 15 | 15 | 14 | 15 | 15 | 15 | 15 | 14 | 14 | 14 | 12 | 12 |  | 13 | 14 | 12 | 15 |
| **41** | 10 | 13 | 13 | 11 | 11 | 13 | 13 | 12 | 12 | 11 | 10 | 13 | 11 | 13 | 13 | 13 | 12 | 13 | 12 | 13 | 13 | 13 | 13 | 13 | 13 | 13 | 13 | 13 | 13 | 13 | 13 | 13 | 13 | 13 | 12 | 12 | 12 | 10 | 10 | 13 |  | 13 | 11 | 13 |
| **42** | 12 | 16 | 16 | 10 | 10 | 15 | 15 | 14 | 14 | 14 | 13 | 16 | 14 | 16 | 16 | 15 | 14 | 16 | 13 | 15 | 16 | 16 | 16 | 16 | 16 | 16 | 16 | 16 | 15 | 14 | 16 | 15 | 15 | 15 | 15 | 15 | 15 | 12 | 12 | 14 | 13 |  | 13 | 16 |
| **43** | 11 | 13 | 13 | 9 | 9 | 13 | 13 | 12 | 13 | 12 | 11 | 13 | 12 | 13 | 13 | 13 | 12 | 13 | 11 | 12 | 13 | 13 | 13 | 13 | 13 | 13 | 13 | 13 | 13 | 12 | 13 | 13 | 13 | 13 | 13 | 12 | 12 | 9 | 9 | 12 | 11 | 13 |  | 13 |
| **44** | 13 | 17 | 17 | 11 | 11 | 16 | 16 | 15 | 15 | 14 | 14 | 17 | 15 | 17 | 17 | 16 | 15 | 17 | 14 | 16 | 17 | 17 | 17 | 17 | 17 | 17 | 17 | 17 | 16 | 15 | 17 | 16 | 16 | 16 | 16 | 16 | 16 | 13 | 13 | 15 | 13 | 16 | 13 |  |

channels

**Supplementary Table 10** Degrees of freedom of the paired t-tests between 24 months and 36 months in each connection.

channels


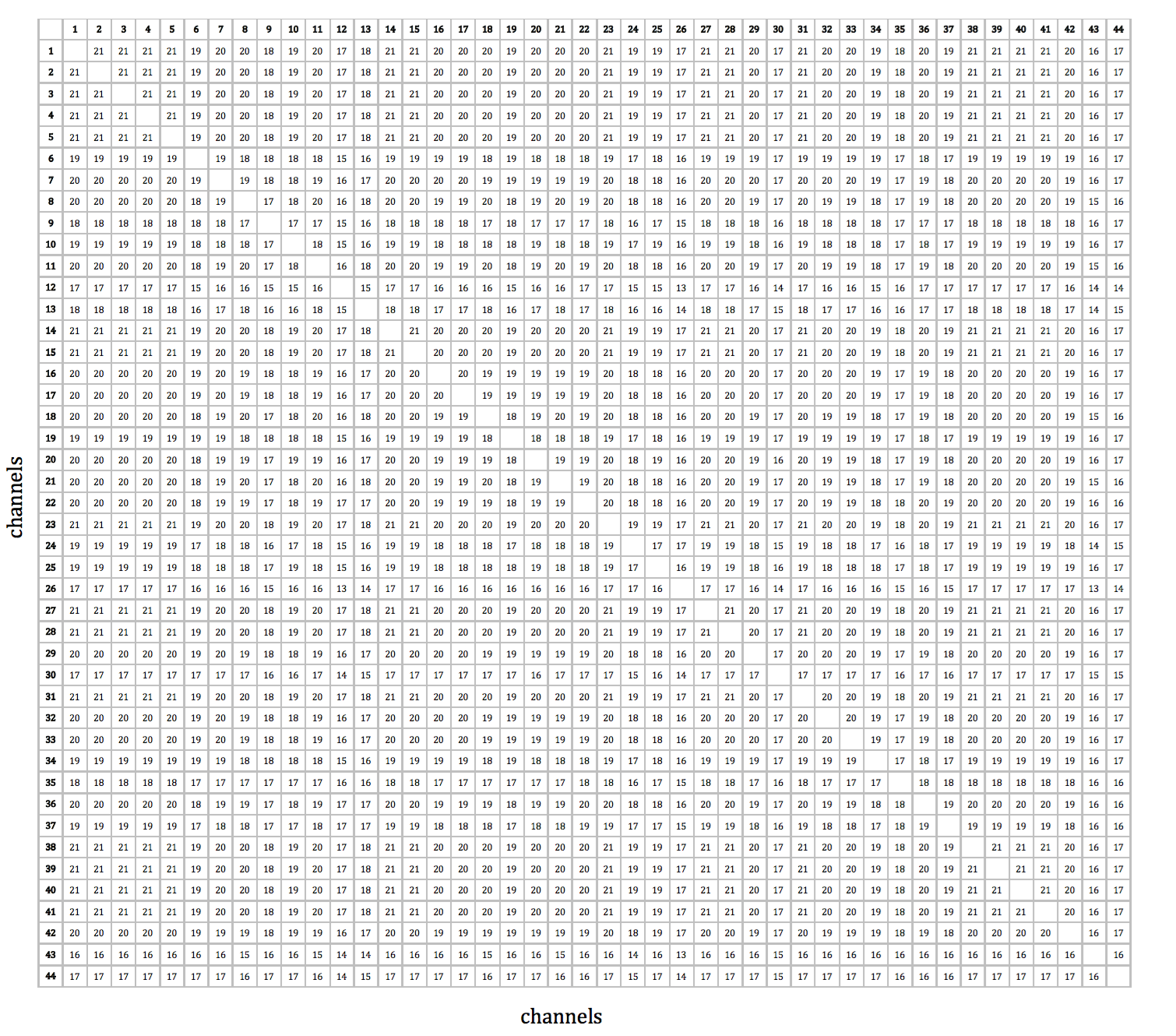


**Supplementary Table 11** Degrees of freedom of the paired t-tests between 30 months and 36 months in each connection.
